# Validity, reliability, responsiveness, and clinically meaningful change threshold estimates of the National Comprehensive Cancer Network-Functional Assessment of Cancer Therapy-Breast Cancer Symptom Index (NFBSI-16)

**DOI:** 10.1186/s41687-024-00776-y

**Published:** 2024-08-15

**Authors:** Nathan A. Clarke, Brendon Wong, Rachael Lawrance, Anders Ingelgård, Ingolf Griebsch, David Cella, Andrew Trigg

**Affiliations:** 1 Adelphi Values, Adelphi Mill, Bollington, Cheshire, SK10 5JB UK; 2 Boehringer Ingelheim International GmbH, Ingelheim am Rhein, Germany; 3 Northwestern University, Chicago, IL USA

**Keywords:** Advanced breast cancer, NFBSI-16, Psychometric, Meaningful change, Minimal important difference, Patient-reported outcomes, Patient experience data

## Abstract

**Background:**

Breast cancer is one of the most common cancers in women. Patient-reported outcome measures are used to evaluate patients’ health-related quality of life in clinical breast cancer studies. This study evaluated the structure, validity, reliability, and responsiveness of the National Comprehensive Cancer Network-Functional Assessment of Cancer Therapy-Breast Cancer Symptom Index (NFBSI-16) subscales in a clinical trial featuring patients with advanced/metastatic breast cancer (aBC), and estimated NFBSI-16 meaningful change thresholds.

**Methods:**

Data from 101 patients with aBC enrolled in a phase II trial (Xenera-1) were included for psychometric evaluation of the NFBSI-16. Subscale structure was evaluated by assessing inter-item correlations, item-total correlations, and internal consistency (cycles 2 and 5). Validity was assessed using scale-level convergent validity (cycles 2 and 5) and known-groups (Baseline). Reliability was analysed via test-retest at cycles 3–4, and responsiveness to improvement and worsening was evaluated at cycles 5, 7, and 9. Meaningful change thresholds were estimated using anchor-based methods (supported by distribution-based methods) at cycles 5, 7, and 9.

**Results:**

NFBSI-16 internal consistency was acceptable, but item-total correlations suggested that its subscales and the GP5 item (side-effect of treatment) scores may be preferred over a total score. Convergent and known-groups evidence supported NFBSI-16 validity. Test-retest reliability was good to excellent for Total and DRS-P (disease-related symptoms: physical) scales, and moderate for the GP5 item. Responsiveness to worsening was generally demonstrated, but responsiveness to improvement could not be demonstrated due to limited observed improvement. Anchor-based meaningful change thresholds were estimated for DRS-P and Total scores.

**Conclusion:**

This study provides evidence that the NFBSI-16 has desirable psychometric properties for use in clinical studies in aBC. It also provides estimates of group- and individual-level meaningful change thresholds to facilitate score interpretation in future aBC research.

**Supplementary Information:**

The online version contains supplementary material available at 10.1186/s41687-024-00776-y.

## Background

Breast cancer is one of the most common cancers in women. More than 180,000 deaths due to breast cancer are expected annually in the United States and Europe combined, and more than 680,000 globally [1]. While early-stage disease is curable, median overall survival for advanced breast cancer (aBC) patients remains poor.

Patient-reported outcomes (PROs) are typically used to provide insight into a patient’s experience of living with cancer. Disease-specific PROs attempt to capture aspects that are most relevant to a condition, such as disease symptoms, treatment side effects, and daily functioning. One such measure for breast cancer is the National Comprehensive Cancer Network-Functional Assessment of Cancer Therapy-Breast Cancer Symptom Index (NFBSI-16) [2, 3].

The NFBSI-16 was developed using methods consistent with recent regulatory guidance [4–6] on PROs as endpoints in clinical trials, with emphasis on patient input during the development process. Conceptual relevance and content validity of the NFBSI-16 has been supported with cognitive debriefing interviews [2, 3]. However, to date, the psychometric properties of the NFBSI-16 have not been investigated.

The aim of this study was to undertake the first comprehensive psychometric evaluation of the NFBSI-16 in patients with aBC using clinical trial data. This included assessing the subscale structure, validity, reliability, and responsiveness of the NFBSI-16, as well as estimating thresholds for interpreting clinically meaningful change on the NFBSI-16 in patients with aBC.

## Methods

### Data source

Analyses were conducted using data from the phase II Xenera-1 clinical trial (NCT03659136). Xenera-1 was a multi-centre, double-blind, randomised trial assessing the efficacy of xentuzumab, a humanized IgG1 insulin-like growth factor monoclonal antibody, in combination with everolimus and exemestane compared to everolimus and exemestane alone in post-menopausal female patients with histologically confirmed hormone receptor positive (HR+)/human epidermal growth factor receptor 2 negative (HER2−) locally advanced or aBC and non-visceral disease. Results of the clinical trial outcomes were published in June 2023 [7].

Data were collected in 11 countries (Belgium, France, Germany, Greece, Italy, Portugal, Spain, UK, Canada, US, Australia) between January 2019 and September 2021. Xenera-1 was carried out in accordance with the principles of the Declaration of Helsinki, in accordance with the International Conference for Harmonisation clinical practice guidelines, in accordance with applicable regulatory requirements, and in compliance with the trial protocol. Informed consent was obtained from each study participant.

### Study measures

PRO questionnaires administered in Xenera-1 included the NFBSI-16 (which includes the single GP5 item), Brief-Pain Inventory-Short Form (BPI-SF) [8], Euroqol 5-Dimension Health Status Self-Assessment (EQ-5D-5L) [9] and eight items from the Patient-Reported Outcomes version of the Common Terminology Criteria for Adverse Events (PRO-CTCAE) library (difficulty swallowing, skin cracking at corners of the mouth, decreased appetite, vomiting, heartburn, diarrhoea, pain in the abdomen, rash) [10, 11]. Questionnaires were administered at Baseline, day 1 from cycles 2 to 20 (every 28 days), then day one of every second cycle (every 56 days) until end of treatment. Patients were treated in 28-day cycles until the end of cycle 20 (week 80) and in 56-day cycles from cycle 21 until they had progressive disease occurrence of intolerable AEs or withdrew consent. The following NFBSI-16 language versions were used for the 11 countries: Dutch, French, German, Greek, Italian, Portuguese, Spanish, English. Responses from the NFSBI-16 (including GP5 item) and EQ-5D-5L questionnaires were used in this psychometric analysis.

In addition to data from PRO questionnaires, the Eastern Cooperative Oncology Group (ECOG) performance status (PS) collected at clinical visits was used as an anchor measure. The Analgesic Quantification Algorithm (AQA) was completed by investigators to assess analgesic use [11].

### Analysis set

The analysis sample for the Psychometric Evaluation Population consisted of patients in Xenera-1 who completed at least one item on either the NFBSI-16 or BPI-SF at any timepoint.

### NFBSI-16

The NFBSI-16 is a 16-item assessment of disease related symptoms, treatment side effects, and general function and well-being. The instrument has four subscales: Disease-Related Symptom—Physical (DRS-P, 8 items; score range: 0–32), Disease-Related Symptoms—Emotional (DRS-E, 1 item; score range: 0–4); Treatment Side-Effect (TSE 4 items; score range: 0–16) (not analysed in this study); and Functional Well-Being (FWB, 3 items; score range: 0–12). A total score can be obtained for the NFBSI-16 (16 items; score range: 0–64). The GP5 item measures bother from side effects [12]. All items have a seven-day recall period and a five-point verbal descriptive response scale (0 = “not at all”, 1 = “a little bit”, 2 = “somewhat”, 3 = “quite a bit”, and 4 = “very much”). Reverse scoring is applied to negatively worded items, so that higher scores are better than lower ones, indicating less symptomatology and better functioning and health-related quality of life.

### Analyses

Psychometric evaluation of the NFBSI-16 was performed in accordance with best practices, and consistent with European Medicine Agency (EMA) and US Food and Drug Administration (FDA) guidance relating to PROs [4, 13].

Scoring and missing item-level PRO data were managed in accordance with developer scoring documentation Version 2_0 [14]. Selection of timepoints was related to the intent of each analysis and expected timeframes for improvement/declines based on expected clinical responses for patients with aBC in the clinical trial, with timepoints selected from baseline and up to cycle 12 (week 44 post randomization) being considered for psychometric analysis.

#### Item responses

The completion rate and distribution of item responses, including evaluation of potential floor and ceiling effects were explored at baseline and each cycle up to cycle 9 using descriptive summaries.

#### Subscale structure of the NFBSI-16

Data at cycle 2 (week 4) and cycle 5 (week 16) were selected as supportive timepoints for assessment of subscale structure. It was expected the number of patients on treatment would still be high, but that patients would have experienced changes in disease-related symptoms and side effects due to treatment.

##### Inter-item correlation

Inter-item correlations for NFBSI-16 were assessed at cycle 2 and repeated at cycle 5 to evaluate potential item redundancy (i.e., items with a correlation > 0.9) [15].

##### Item-total correlation

For assessing item-total correlations in terms of item-level convergent and discriminant validity, multitrait analysis was used [16]. The analysis was completed at Cycle 2 and repeated at Cycle 5. All ordinal NFBSI-16 item responses were assessed using polyserial correlation coefficients with multi-item subscales scores (Total score, DRS-P and FWB, all assumed continuous); subscales were corrected for overlap (i.e., the subscale score was calculated with the removal of the item with which it was correlated). Items with a scaling success ≥ 0.4 were considered correlated [16], and problematic if the highest correlation was with a different subscale.

##### Internal consistency

Internal consistency was examined by calculating Cronbach’s alpha coefficient at Cycle 2 and repeated at Cycle 5 for NFBSI-16 Total, DRS-P, and FWB subscales. Internal consistency was considered acceptable if the alpha coefficient was ≥ 0.70 [15]. The impact of item removal on internal consistency reliability was also examined.

#### Validity

##### Scale-level convergent validity

A priori hypothesised relationships between NFBSI-16 scores and items of EQ-5D-5L were defined based on consideration of the concepts of the item; the EQ-5D-5L VAS, as a general measure of HRQoL was hypothesized to show convergent validity with all NFBSI-16 subscales. Polyserial correlations were estimated; correlation coefficients ≥|0.3|were considered supportive of convergent validity [17].

##### Known-groups comparisons (construct validity)

Known-groups based on clinical characteristics of patients were selected a priori. The baseline characteristics of ECOG PS (1 vs 0) was selected to identify clinically distinct groups of patients, where ECOG PS status 0 represents fully active patients and ECOG PS 1 represents some level of physical impairment. Additional known-groups were selected based on key study entry criteria of ‘bone metastases at screening’, ‘measurable disease at Baseline’, and ‘number of previous lines of therapy’.

For known-groups, score differences were tested using one-way analysis of variance (ANOVA), with *p* < 0.05 (not adjusted for multiple comparisons). The magnitude of differences was assessed using between-group effect size (ES; Cohen’s d) estimates, using the pooled standard deviation as the denominator. Between-group ES estimates were classified using conventional benchmarks of “small” (0.2), “medium” (0.5), and “large” (0.8) [17].

#### Reliability—test retest

Test-retest reliability of the NFBSI-16 was examined in subsets that were considered stable according to various anchor measures. Cycle 3 (week 8) and Cycle 4 (Week 12) were selected as timepoints most likely to reflect stable PRO outcomes (i.e., after initial response to treatment and prior to deterioration). The anchor measures selected were performance status (ECOG PS), and the widely used general HRQoL PRO EQ-5D-5L. Groups were defined as stable based on no change in response level (for ECOG PS and EQ-5D-5L items) or a change in EQ-5D-5L VAS score of < 7 points, aligning with a clinically meaningful change in VAS score [18] (see Supplementary Table 1 for overview of anchors). Test-retest reliability was investigated by calculating the intraclass correlation coefficient ICC_[2,1]_ [19]. The following threshold values were employed to interpret ICC values: values less than 0.5 are indicative of poor reliability, values between 0.5 and 0.75 indicate moderate reliability, values between 0.75 and 0.90 indicate good reliability, and values greater than 0.90 indicate excellent reliability [20].

#### Responsiveness—improvement/worsening

Change from baseline to cycle 5, cycle 7, and cycle 9 (weeks 16, 24, and 32) were selected as timepoints where improvement or deterioration may be expected, but before a high proportion of patients experienced disease progression. ECOG PS and EQ-5D-5L were defined as anchor measures for evaluation of responsiveness as both are well established measures known to show responsiveness to change in oncology patients. For ECOG PS changes of 1 level were considered as improvement/worsening. For EQ-5D-5L items changes in response of 1 level were considered as improvement/worsening. For EQ-5D-5L VAS a change in score ≥ 7 points was improvement and £ 7 points as worsening [18].

For each subscale, and the single GP5 item, mean change scores were assessed using within-group ES estimates [21] (mean change score divided by the SD_Baseline)_ and interpreted descriptively using typical benchmarks of “small” (0.2), “medium” (0.5), and “large” (0.8) [17]. One-way ANOVA F-tests were used to evaluate the statistical significance (*p* ≤ 0.05) of differences in change scores between groups.

#### Meaningful change

Timepoints for meaningful change analyses were selected to reflect longitudinal data for patients up to 32 weeks from randomisation. PRO assessments were collected at later timepoints, but it was expected that data would be missing over time as patients stopped treatment due to disease progression/death. Meaningful change in scores for the NFBSI-16 Total and subscales were evaluated at both the group- and individual-level. Anchor-based and distribution-based methods were used.

Three types of meaningful change threshold were considered. First, a clinically important change (CIC): a change over time within a group considered clinically relevant. Second, a clinically important difference (CID) between treatment groups considered clinically relevant [22]. The CID was defined as the difference between mean change scores for the Stable and Improved/Stable and Worsened groups. Third, a clinically important responder (CIR) (meaningful within-patient change): the amount of change a patient would have to report to indicate that a relevant treatment benefit has been experienced [22]. These thresholds were not necessarily minimal in nature (i.e., reflecting the smallest change that patients perceive as important) [22, 23], due to merging minimal and moderate anchor categories.

Anchor and NFBSI-16 score pairings exhibiting polyserial (NFBSI-16 Total, DRS-P, and FWB scores) and polychoric (NFBSI-16 DRS-E and GP5 item) correlation ≥|0.3| [24] for at least two of the three assessment timepoints were taken forward for further score analyses [25, 26].

Mixed models for repeated measures (MMRMs) were used to evaluate CIC and CID thresholds across multiple timepoints, all of which were incorporated into a single longitudinal [27] model, enabling an increased number of observations to contribute to estimates. Least squares (LS) mean estimates for the Minimal-to-Moderate Improved and Minimal-to-Moderate Worsened groups corresponded to estimates of the CIC for improvement/worsening. The CID was estimated as the difference in LS means between the Minimal-to-Moderate improvement/worsening and Stable groups.

Receiver operating characteristic (ROC) curve analysis was used to inform CIR estimates. Potential CIR estimates (i.e., all possible change scores) were evaluated by finding an optimal cut-point between stable and improved or worsened groups (collapsing Minimal/Moderate/Major improvement/worsened groups) according to ROC curves using the sum of squares method (i.e., Min[(1 − sensitivity)^2^ + (1 − specificity)^2^]) [28].

Supportive empirical cumulative distribution function (eCDF) plots were also produced to evaluate the performance of proposed CIR thresholds, as per US FDA guidance [29].

A lower and upper standard error of measurement (SEM) was calculated, using two ICCs from test-retest reliability analyses as reliability coefficients (i.e., the lower SEM was calculated using the smallest ICC, the upper ICC was calculated using the largest). While the SEM was not intended as an estimate of importance thresholds [30], score changes beyond this threshold are more likely than not to be free from measurement error.

For triangulation, where multiple CIC, CID, or CIR threshold estimates were obtained, the largest of the estimated thresholds was selected to ensure that results were clinically meaningful. Additionally, the SEM of NFBSI-16 scores was taken into consideration to inform CIR estimates that exceeded measurement error.

## Results

### Baseline characteristics, item responses and completion rate

There were 101 patients included in the Psychometric Evaluation Population. The mean age of study patients was 60.56 years (SD = 10.59), with ages ranging from 29 to 84. Table 1 provides further detail regarding the demographic and clinical characteristics for the Psychometric Evaluation Population. The mean scores for NFBSI-16 subscales, EQ-5D-VAS, and proportion with GP5 item responses at baseline are presented in Table 2.

**Table 1:** Demographic and clinical characteristics at baseline

Demographic	Psychometric evaluation population (N = 101)
Age	
£ 65 years	62 (61.4%)
> 65 years	39 (38.6%)
Race/ethnicity	
Asian	1 (1.0%)
Non-Asian	86 (85.1%)
Missing	14
Region	
Europe	69 (68.3%)
North America	28 (27.7%)
other	4 (4.0%)
Measurable disease at baseline	
Yes	50 (49.5%)
No	51 (50.5%)
Prior treatment with fulvestrant	
Yes	44 (43.6%)
No	57 (56.4%)
Prior treatment with WHO-DD ATC	
Yes	49 (48.5%)
No	52 (51.5%)
Prior treatment with adjuvant chemotherapy	
Yes	70 (69.3%)
No	31 (30.7%)
Baseline ECOG performance status	
0	66 (65.3%)
1	35 (34.7%)
Number of previous lines of therapy	
1	56 (55.4%)
>1	31 (30.7%)
Missing	14
Endocrine resistance	
Primary	26 (25.7%)
Secondary	75 (74.3%)

*Abbreviations WHODD* world health organisation drug dictionary (WHO-DD), anatomical therapeutic chemical (ATC) Classification level 3 of ‘Drugs affecting bone structure and mineralization’, *ECOG* Eastern cooperative oncology group

**Table 2:** Baseline NFBSI-16 and EQ-5D-5L VAS scores

PRO instrument/scale	Baseline score
NFBSI-16 total	
N	97
Mean (SD)	43.3 (10.19)
NFBSI-16 DRS-P	
N	97
Mean (SD)	20.7 (6.63)
NFBSI-16 DRS-E	
N	96
Mean (SD)	1.5 (1.35)
NFBSI-16 FWB	
N	97
Mean (SD)	6.9 (3.22)
NFBSI-16 GP5 Item (n, %)	
0	64 (63.4%)
1	15 (14.9%)
2	6 (5.9%)
3	2 (2.0%)
4	2 (2.0%)
Missing	12 (11.9%)
EQ-5D-5L VAS	
N	97
Mean (SD)	67.4 (20.82)

For patients remaining on treatment from baseline to cycle 12, completion for the NFBSI-16 was high across items, with missing data rates for all items less than 8.2% across all visits except Item B5 (‘I am bothered by hair loss’) at Cycle 12 (missing = 15%). For the key timepoints of interest in this study (up to cycle 5) the available data included over 62 patients (out of 101 patients in the analysis Psychometric Evaluation Population). NFBSI-16 Total: Baseline *n* = 97 (96%), cycle 2 *n* = 93 (92%), cycle 3 *n* = 78 (77%) and cycle 5 *n* = 62 (61%). Across Baseline to Cycle 12 only a single individual had more than two NFBSI-16 items missing; 10 individuals (10.3%) were missing 2 items at baseline, with less than 3 individuals at all other timepoints.

At baseline, some patients reported responses of “Not at all” i.e., no level of problems at baseline and therefore the responses of these patients could not improve which could lead to a ceiling effect being observed in analysis at later timepoints: Items GP3 (‘I have trouble meeting family needs’, B1 (‘I have been short of breath’), BP1 (‘I have bone pain’), GP6 (‘I feel ill’), and HI7 (‘I feel fatigued’) within the DRS-P scale all showed patterns of having potential ceiling effects. Also, for GP5 (‘I am bothered by side effects of treatment’) 63% of patients reported “Not at all” at baseline.

### Subscale structure of the NFBSI-16

#### Inter-item-correlations

No items were deemed redundant in terms of inter-item correlations (all correlations < 0.9).

#### Item-total correlation

Item-total correlations (corrected for overlap) at Cycle 2 for NFBSI-16 Total score were > 0.4, except for items B1 (‘I have been short of breath’; *r* = 0.36), GF5 (‘I am sleeping well’; *r* = 0.34), GE6 (‘I worry condition will get worse; *r* = 0.33), N6 (‘I have mouth sores’; *r* = 0.23), and B5 (‘I am bothered by hair loss’; *r* = 0.27). Regarding the DRS-P scale, all item-total correlations were > 0.4 except for item GF5 (‘I am sleeping well’; *r* = 0.32). Some items exhibited higher correlations with other subscales than their corrected subscale, including item GF5 (‘I am sleeping well’) correlating better with FWB (*r* = 0.42) than NFBSI-16 DRS-P (*r* = 0.32). Similar patterns were also seen at Cycle 5 (Supplementary Tables 2.1 and 2.2).

#### Internal consistency

At cycles 2 and 5, internal consistency was high (i.e., > 0.8) for all scales. At cycle 2, alpha for the NFBSI-16 Total was 0.844; for DRS-P it was 0.832. Following individual item deletion, changes to alpha values were negligible (NFBSI-16 Total range: 0.820–0.855; DRS-P range: 0.788–0.852).

### Validity

#### Scale-level convergent validity

Scale-level convergent correlations for NFBSI-16 subscales were larger than the hypothesized convergent criteria (i.e., ≥ 0.3) (Table 3). This pattern was also seen at cycle 5 (data not presented).

**Table 3:** Scale-level convergent validity of NFBSI-16 with EQ-5D-5L hypothesized measures

Cycle 2	EQ-5D-5L				
NFBSI-16	Pain	Depression /anxiety	Mobility	Usual activities	VAS
NFBSI-16 total					*(≥0.3)* **0.635**
NFBSI-16 DRS-P	*(≥0.3)* **−0.635**		*(≥0.3)* **−0.544**	*(≥0.3)* **−0.752**	*(≥0.3)* **0.648**
NFBSI-16 DRS-E		*(≥0.3)* **−0.362**			*(≥0.3)* 0.290
NFBSI-16 FWB		*(≥0.3)* **−0.315**		*(≥0.3)* **−0.508**	*(≥0.3)* **0.525**
GP5					*(≥0.3)* **0.341**

Results for Cycle 2; NFBSI scores (except DRS-E) use polyserial correlations (except EQ-5D-5L VAS, which is Spearman). Polychoric correlations used for GP5 Item and DRS-E (except with EQ-5D-5L VAS, where polyserial correlations used)

Values are bold where hypothesised correlation thresholds were met. Hypothesised correlations are shown in brackets: convergent criteria (≥0.3)

#### Known-groups comparisons

For NFBSI-16 Total score (Table 4), NFBSI-16 DRS-P, and NFBSI-16 FWB there were statistically significant differences in scores for each of the baseline ECOG performance groups, with lowest mean NFBSI-16 scores being seen in the EOCG PS 0 status patients. The between group differences were large (i.e., effect sizes ≥ 0.80) and statistically significant (*p* < 0.001) for NFBSI-16 Total score and NFBSI-16 DRS-P and moderate for NFBSI-16 FWB. Other baseline characteristics of ‘bone metastases at screening’, ‘measurable disease at baseline’ and ‘number of previous lines of therapy were also considered as potentially identifying clinically distinct patients, although no strong evidence for differences in NFBSI-16 scores were noted for these groups.

**Table 4:** Known groups at baseline: ECOG PS vs NFBSI-16

Baseline NFBSI-16 subscale	Baseline ECOG PS 0 (*N* = 64)	Baseline ECOG PS 1 (*N* = 33)	Between groups effect size (Hedges’s G)	Pairwise test *p*-value
NFBSI-16 total score (Mean (SD))	46.07 (9.38)	37.81 (9.60)	0.87	<0.01
NFBSI-16 DRS-P (Mean (SD))	22.38 (6.01)	17.39 (6.60)	0.80	<0.01
NFBSI-16 FWB (Mean (SD)	7.58 (3.29)	5.55 (2.62)	0.66	0.03

*Abbreviations SD* standard deviation, *ECOG* Eastern cooperative oncology group; Between-group effect size is Hedge’s g compared to the reference group. Hedge’s g is calculated as the difference in means divided by the pooled standard deviation. F-test of one-way ANOVAs used to calculate statistical significance of differences in scores between groups

### Reliability

Test-retest reliability was assessed in stable patients between cycle 3 (Week 9) and cycle 4 (Week 12), resulting in subsets of between 37 to 54 patients. The ICCs for the anchors for each NFBSI-16 subscale (Table 5) were moderate to excellent across all scales and anchors (ICC > 0.6).

**Table 5:** NFBSI-16 test-retest reliability analysis between cycle 3 (week 8) and cycle 4 (week 12)

NFBSI-16 subscale score/anchor	n	Intraclass correlation coefficient	Lower 95% CI	Upper 95% CI
NFBSI-16 total				
EQ-5D VAS	38	0.901	0.818	0.947
ECOG PS	54	0.842	0.743	0.905
NFBSI-16 DRS-P				
EQ-5D VAS	38	0.906	0.827	0.95
ECOG PS	54	0.830	0.723	0.898
NFBSI-16 DRS-E				
EQ-5D depression/anxiety	49	0.695	0.516	0.816
EQ-5D VAS	37	0.635	0.396	0.794
NFBSI-16 FWB				
EQ-5D depression/anxiety	50	0.724	0.560	0.834
EQ-5D VAS	38	0.701	0.494	0.833
GP5: I am bothered by treatment side effects				
EQ-5D VAS	37	0.614	0.369	0.78

Test-retest population defined from cycle 3 to cycle 4 as subjects with (A) < 7-point change in EQ-5D-5L VAS (in either direction); (B) no change in ECOG performance status; (C) no change in EQ-5D depression/anxiety response

### Responsiveness

Responsiveness to worsening was demonstrated with large within-group effect sizes between Baseline and Cycle 5 for the NFBSI-16 Total score (ES = −1.05; *p* = 0.043), DRS-P (ES = −0.53; *p* = 0.081), and GP5 item (ES = −2.81, *p* = 0.018). Responsiveness to worsening was also demonstrated for other NFBSI-16 scales (Data not presented).

### Meaningful change

NFBSI-16 Total score, DRS-P, and DRS-E scales had anchors (EQ-5D-5L VAS, pain, usual activities) that reached acceptable thresholds (i.e., anchor correlation of ≥|0.3|for at least two of three timepoints assessed (cycle 5, 7, 9). Table 6 provides an overview of all available anchor-based estimates for these scales. The remaining NFBSI-16 scales were insufficiently correlated with potential anchors (Supplementary Table 3) and could only have meaningful change explored via distribution-based methods.

**Table 6:** Overview of anchor-based CIC, CID, and CIR estimates for NFBSI-16 scales

NFBSI-16	Anchor (EQ-5D-5L)	Improved or worsened	CIC	CID	CIR
Total score	VAS	Improved	0.0 or N/A	N/A	N/A
	VAS	Worsened	−7	−4.8	−3.4 *(Cycle 5)* −7 *(Cycle 9)*
DRS-P	Pain	Improved	1.2	2	
	Usual activities	Improved			6 (Cycle 5)
	Pain	Worsened	−2.6	−1.8	−3 *(Cycle 5)*
	Usual activities	Worsened	−2.9	−2.4	−4 *(Cycle 7)* −2 *(Cycle 9)*
DRS-E	VAS	Improved	0.4	0.1	N/A
	VAS	Worsened	0.1*	0.2*	−1 *(Cycle 5)*

*Abbreviations CIC* clinically important change, *CID* clinically important difference, *CIR* clinically important responder

All CIC and CID estimates obtained from MMRMs (the dependent variable was change from baseline in PRO score of interest, up to and including Cycle 12 (week 44). Fixed effects of categorical timepoint and categorical anchor were incorporated in addition to the interaction between these two variables. A repeated effect of visit was specified to account for correlation between multiple observations of the same patient. A heterogeneous compound symmetry covariance structure was used.)

All CIRs estimates derived from ROC curve analysis where AUC CIs did not include 0.500 (i.e., better than chance classification)

*Ineligible CIC/CID estimate for DRS-E Worsened with EQ-5D-5L VAS because positive score represents improved health state

Improved estimates for DRS-P according to EQ-5D-5L Usual Activities were non-estimable likely due to low numbers of observations within this group over time

For NFBSI-16 Total score (with a range of 0–64 points), a single anchor (EQ-5D-5L VAS) was sufficiently correlated to derive score interpretation threshold estimates. For the improved group, the CIC was unevaluable because a change score of 0.0 was estimated (subsequently invalidating the CID); furthermore, no CIR threshold estimates provided better than chance discrimination (i.e., AUC CIs included 0.500). For the worsened group, estimates indicated that a CIC of −7, and CID of −4.8 were appropriate; as was a CIR threshold of −7, which would surpass the upper estimate of SEM (SEM = 4.1) and correctly classify most worsened patients as evidenced by eCDF plots and ROC curves (Supplementary Fig. 1.1.x.x (eCDFs) and Fig. 2.1.x.x. (ROC Improved) and Fig. 3.1.x.x (ROC Worsening) and Supplementary Table 4.1.x).

For DRS-P (with a range of 0–32 points), two anchors (EQ-5D-5L Pain and EQ-5D-5L Usual Activities) were sufficiently correlated to derive score interpretation threshold estimates. For the improved group, estimates indicated that a CIC of 1.2 and CID of 2 were appropriate; as was a CIR threshold of 6, which would surpass the upper estimate of SEM (SEM = 3.8). For the worsened group, estimates indicated that CICs of −2.6 to −2.9 and CIDs of −1.8 to −2.4 were appropriate, as was a CIR threshold of −4. eCDFs were generally considered supportive of proposed DRS-P thresholds (Supplementary Fig. 1.2.x.x (eCDFs) and Fig. 2.2.x.x (ROC Improved) and Fig. 3.2.x.x (ROC Worsening) and Supplementary Table 4.2.x).

For DRS-E (range 0–4 points), a single anchor (EQ-5D-5L VAS) was sufficiently correlated to derive score interpretation threshold estimates. For the improved group, estimates indicated that a CIC of 0.4, and CID of 0.1 were appropriate; no CIR was estimable because ROC curve AUC CIs included 0.500 (i.e., indicating no better than chance classification). For the worsened group, the CIC and CID were inadmissible because positive scores were estimated (representing an improved health state); for the CIR, an estimate of −1 was indicated, which would surpass the upper estimate of SEM (SEM = 0.812) and supported correctly classifying most worsened patients plots (Supplementary Fig. 1.3.x.x (eCDFs) and Fig. 2.3.x.x (ROC Improved) and Fig. 3.3.x.x (ROC Worsening) and Supplementary Table 4.3.x).

## Discussion

This study evaluated the psychometric properties of the NFBSI-16 and provided estimates of meaningful change thresholds. It is the first study to provide evidence of the psychometric properties of the NFBSI-16 and its score interpretation in an aBC patient population.

Internal consistency was high for NFBSI-16 Total and DRS-P scales (i.e., > 0.8). Although inter-item correlations did not indicate redundancy of any NFBSI-16 items (i.e., all correlations were < 0.9), some items displayed low item-total correlations with their intended scales or larger correlations with other scales than those intended across cycles 2 and 5 (Supplementary Tables 2.1 and 2.2). These included items B1 (‘I have been short of breath’), GF5 (‘I am sleeping well’), GE6 (‘I worry condition will get worse’), N6 (‘I have mouth sores’), and B5 (‘I am bothered by hair loss’) with NFBSI-16 Total; and GF5 (‘I am sleeping well’) with NFBSI-16 DRS-P. These results suggest that scores from the NFBSI-16 subscales, not the Total score, may be the preferred focus in future clinical research. Future research may also explore the performance of the DRS-P scale without the inclusion of item GF5 (‘I am sleeping well’), which fit less-well within this subscale as indicated in the internal consistency reliability and multi-trait analyses. Similar issues concerning the inclusion of an item assessing sleep in a related ovarian cancer measure have been reported [31].

Test-retest reliability evidence was generally adequate, with good to excellent reliability for Total and DRS-P, and moderate reliability for the GP5 item. This provides supportive evidence of reliability of the GP5 item to assess overall treatment tolerability in patients with aBC, adding to work that has investigated its validity in a diverse sample of cancer patients [32].

A summary of proposed score interpretation thresholds for the NFBSI-16 is presented in Table 7, with indicated threshold estimates rounded to the nearest whole number for simplicity. A process of triangulation is generally recommended for multiple meaningful change threshold estimates [24, 33–35]; in this study, when multiple estimates were available, the largest of the available estimates was included as the proposed score. There were no anchor-based estimates for the GP5 Item scores due to a lack of acceptable correlations with anchor measures; therefore, distribution-based estimates (i.e., the SEM) form the basis of these proposed thresholds. SEM estimates also informed proposed thresholds in the absence of eligible estimates for specific improved (NFBSI-16 Total) and worsened (DRS-E).

**Table 7:** Summary of proposed CIC, CID, and CIR thresholds for NFBSI-16 scales

NFBSI-16 score	CIC (improved)	CIC (worsened)	CID	CIR (improvement)	CIR (worsened)
Total	4*	−7	5	4*	−7
DRS-P	2	−3	2	6	−4
DRS-E	1	−1*	1	1*	−1
FWB*	2	−2	2	2	−2
GP5 Item*	1	−1	1	1	−1

*Abbreviations CIC* clinically important change, *CID* clinically important difference, *CIR* clinically important responder

*Score interpretation thresholds based solely on SEM estimates due to unavailability of appropriate anchor (i.e., no anchors correlated ≥ 0.3 with NFBSI-16 score) or invalid estimate arising from anchor estimate (Supplementary Table 5 Distribution based estimates)

It should be noted that there are various issues in the score interpretation literature for which there is no consensus; these include consensus on terminology, optimal estimation methodology, and indices of likely change [36]. Subsequently, the proposed thresholds in this study represent a broader clinically meaningful change compared to a minimal threshold (as they are based on merged minimal and moderately changed groups). Meaningful change thresholds varied depending on the overall score range for the NFBSI-16; i.e., 5–7 points were indicated for the 0 to 64-point Total score, while a 1-point threshold was indicated for the 0 to 4- GP5 item score. From a patient perspective, a 1-point change in NFBSI-16 scores equates to a 1-category shift on an item.

The NFBSI-16 treatment side effect (TSE) scale is not presented in these results. The TSE scale contains three items on nausea, mouth sores, and hair loss, as well as an item reflecting global side effect burden (item GP5); however, the introduction of novel therapies since the original development of the NFBSI-16 means additional toxicities are now relevant to patients with aBC. Although we did not recommend removal of any NFSBI-16 items, this scale is not included in the analysis as it is no longer considered content valid in the current clinical setting. FACIT.org no longer recommends scoring the TSE items as a scale, preferring to focus on item GP5 (http://www.facit.org). Psychometric evidence has supported the validity of the GP5 single item across diverse cancer sites and various countries [12, 37–39]. Use of the GP5 single item to assess overall side effect impact alongside items assessing specific symptomatic adverse events, for example from the PRO-CTCAE library, is also aligned with recent FDA guidance [40].

A limitation of this study is the lack of anchor-derived meaningful change thresholds for every NFBSI-16 subscale. The FWB scale, and GP5 item lacked sufficiently correlated anchors for deriving meaningful change threshold estimates. Similarly, meaningful change estimates for improvement were not valid for the NFBSI-16 Total score (i.e., a score of 0 was estimated); therefore, only thresholds for worsening may be used in future applications. Generally, improved and worsened within-individual thresholds were estimated, as well as distribution-based scores that can be used as interpretative benchmarks (albeit do not directly target important change) [41].

It is noted that this is a relatively small sample size (*n* = 101) for psychometric analysis, particularly as oncology study patients experience disease progression and stop treatment; there were only 62 patients who completed the NFBSI-16 at the cycle 5 timepoint. This may have led to the slight inconsistencies observed for some results. Further studies in larger and broader patient populations should be conducted to gather additional psychometric evidence to confirm the scale structure and meaningful change thresholds of the NFBSI-16 in related populations, such as HR+, HER2− and visceral metastases [42], HER2 positive or triple negative breast cancer.

It would also be interesting to better understand how patients reflect on the PROs used and results of this study by undertaking qualitative interviews. Patient experience data like interviews could confirm and deepen the understanding of the results relating them to patients’ experiences of their disease and their treatment. In turn, this may facilitate further understanding of the results of this study. Moreover, qualitative interviews may provide important information that might enhance construction of hypotheses in future clinical cancer trials. For example, new treatments may delay worsening of disease-related symptoms but perhaps not improve the general burden and experience of the cancer disease which could influence the research question.

In summary, the NFBSI-16 has desirable psychometric properties for use in aBC studies given appropriate consideration of its intended use (i.e., given a focus on subscales in future clinical research, and worsening of breast cancer-related severity as the outcome of interest). Furthermore, psychometric properties of the NFBSI-16 may be improved in future studies by considering removal of items that were shown to fit poorly according to developer-recommended scoring (e.g., item GF5), or those with potential baseline ceiling effects that may limit scale responsiveness (e.g., item N6).

## Conclusion

This study provides evidence that the NFBSI-16 has desirable psychometric properties for use in aBC studies given appropriate consideration of its intended use; the study also provides estimates of group- and individual-level meaningful change thresholds for use in future aBC research.

### Electronic supplementary material

Below is the link to the electronic supplementary material.

Supplementary Material 1

Supplementary Material 2

## Data Availability

To ensure independent interpretation of clinical study results and enable authors to fulfil their role and obligations under the ICMJE criteria, Boehringer Ingelheim grants all external authors access to relevant clinical study data. In adherence with the Boehringer Ingelheim Policy on Transparency and Publication of Clinical Study Data, scientific and medical researchers can request access to clinical study data after publication of the primary manuscript in a peer-reviewed journal, regulatory activities are complete and other criteria are met. Researchers should use the https://vivli.org/ link to request access to study data and visit https://www.mystudywindow.com/msw/datasharing for further information.

## Abbreviations

aBC: Advanced/metastatic breast cancer
AQA: Analgesic quantification algorithm
BPI-SF: Brief pain inventory-short form
eCDF: Empirical cumulative distribution function
CIC: Clinically important change
CID: Clinically important difference
CIR: Clinically important responder
DRS-E: Disease-related symptom-emotional
DRS-P: Disease-related symptoms-physical
ECOG: Eastern cooperative oncology group
EMA: European medicine agency
EQ-5D-5L: Euroqol 5-dimension health status self-assessment
ES: Effect size
FDA: Food and drug administration
FWB: Functional well-being
HER2: Human epidermal growth factor receptor 2
HR: Hormone receptor
ICC: Intraclass correlation coefficient
MMRM: Mixed model for repeated measure
NFBSI-16: National comprehensive cancer network functional assessment of cancer therapy-breast cancer symptom index
PRO: Patient-reported outcome
PRO-CTCAE: Patient-reported outcomes-common terminology criteria for adverse events
ROC: Receiver operating characteristic
SD: Standard deviation
SEM: Standard error measurement
TRT: Test-retest
TSE: Treatment side effects

## References

[1] Sung H, Ferlay J, Siegel RL, Laversanne M, Soerjomataram I, Jemal A, Bray F (2021) Global cancer statistics 2020: GLOBOCAN estimates of incidence and mortality worldwide for 36 cancers in 185 countries. Ca A Cancer J Clin 71(3):209–249 10.3322/caac.21660 33538338

[2] Garcia SF, Rosenbloom SK, Beaumont JL, Merkel D, Von Roenn JH, Rao D, Cella D (2012) Priority symptoms in advanced breast cancer: development and initial validation of the National comprehensive cancer Network-Functional assessment of cancer Therapy-Breast cancer symptom index (NFBSI-16). Value Health 15(1):183–190 22264987 10.1016/j.jval.2011.08.1739

[3] Krohe M, Tang DH, Klooster B, Revicki D, Galipeau N, Cella D (2019) Content validity of the national comprehensive cancer network–functional assessment of cancer therapy–breast cancer symptom index (NFBSI-16) and patient-reported outcomes measurement information system (PROMIS) physical function short form with advanced breast cancer patients. Health Qual Life Outcomes 17(1):1–12 31142325 10.1186/s12955-019-1162-5 PMC6542025

[4] Food and Drug Administration (2018) Methods to identify what is important to patients & select, develop or modify fit-for-purpose clinical outcomes assessments

[5] Food and Drug Administration (2022) Patient-focused drug development: methods to identify what is important to patients

[6] Food and Drug Administration (2020) Patient-focused drug development: collecting comprehensive and representative input

[7] Schmid P, Cortes J, Joaquim A, Jañez NM, Morales S, Díaz-Redondo T, Blau S, Neven P, Lemieux J, García-Sáenz JÁ (2023) XENERA-1: a randomised double-blind phase II trial of xentuzumab in combination with everolimus and exemestane versus everolimus and exemestane in patients with hormone receptor-positive/HER2-negative metastatic breast cancer and non-visceral disease. Breast Cancer Res 25(1):67 10.1186/s13058-023-01649-w PMC10258741 37308971

[8] Cleeland CS (2009) The brief pain inventory user guide. The University of Texas MD Anderson Cancer Center, Houston, TX, pp 1–11

[9] Herdman M, Gudex C, Lloyd A, Janssen M, Kind P, Parkin D, Bonsel G, Badia X (2011) Development and preliminary testing of the new five-level version of EQ-5D (EQ-5D-5L). Qual Life Res 20(10):1727–1736 21479777 10.1007/s11136-011-9903-x PMC3220807

[10] Smith AW, Mitchell SA, De Aguiar CK, Moy C, Riley WT, Wagster MV, Werner EM (2016) News from the NIH: person-centered outcomes measurement: NIH-supported measurement systems to evaluate self-assessed health, functional performance, and symptomatic toxicity. Transl Behav Med 6(3):470–474 27528535 10.1007/s13142-015-0345-9 PMC4987601

[11] Chung KC, Barlev A, Braun AH, Qian Y, Zagari M (2014) Assessing analgesic use in patients with advanced cancer: development of a new scale—the analgesic quantification algorithm. Pain Med 15(2):225–232 24400921 10.1111/pme.12299

[12] Pearman TP, Beaumont JL, Mroczek D, O’Connor M, Cella D (2018) Validity and usefulness of a single-item measure of patient-reported bother from side effects of cancer therapy. Cancer 124(5):991–997 29131323 10.1002/cncr.31133 PMC5892190

[13] Food and Drug Administration (2009) Use in medical product development to support labeling claims

[14] FACIT Group. NFBSI–16. facit.org

[15] Nunnally JC (1994) Psychometric theory 3E. Tata McGraw-Hill Education

[16] Cappelleri JC, Zou KH, Bushmakin AG, Alvir JMJ, Alemayehu D, Symonds T (2013) Patient-reported outcomes: measurement, implementation and interpretation. Crc Press

[17] Cohen J (2013) Statistical power analysis for the behavioral sciences. Routledge

[18] Pickard AS, Neary MP, Cella D (2007) Estimation of minimally important differences in EQ-5D utility and VAS scores in cancer. Health Qual Life Outcomes 5:1–8 18154669 10.1186/1477-7525-5-70 PMC2248572

[19] Shrout PE, Fleiss JL (1979) Intraclass correlations: uses in assessing rater reliability. Psychol Bull 86(2):420 18839484 10.1037/0033-2909.86.2.420

[20] Koo TK, Li MY (2016) A guideline of selecting and reporting intraclass correlation coefficients for reliability research. J Chiropr Med 15(2):155–163 27330520 10.1016/j.jcm.2016.02.012 PMC4913118

[21] Kazis LE, Anderson JJ, Meenan RF (1989) Effect sizes for interpreting changes in health status. Med Care S178–S189 10.1097/00005650-198903001-00015 2646488

[22] Coon CD, Cappelleri JC (2016) Interpreting change in scores on patient-reported outcome instruments. Ther Innov Regul Sci 50(1):22–29 30236016 10.1177/2168479015622667

[23] Devji T, Carrasco-Labra A, Guyatt G (2021) Mind the methods of determining minimal important differences: three critical issues to consider. Evid-Based Ment Health 24(2):77–81 32839275 10.1136/ebmental-2020-300164 PMC10231500

[24] Revicki D, Hays RD, Cella D, Sloan J (2008) Recommended methods for determining responsiveness and minimally important differences for patient-reported outcomes. J Clin Epidemiol 61(2):102–109 18177782 10.1016/j.jclinepi.2007.03.012

[25] Griffiths P, Sims J, Williams A, Williamson N, Cella D, Brohan E, Cocks K (2023) Correction: how strong should my anchor be for estimating group and individual level meaningful change? A simulation study assessing anchor correlation strength and the impact of sample size, distribution of change scores and methodology on establishing a true meaningful change threshold. Qual Life Res 32(5):1265. 10.1007/s11136-023-03356-7 36759380 10.1007/s11136-023-03356-7

[26] Griffiths P, Sims J, Williams A, Williamson N, Cella D, Brohan E, Cocks K (2023) How strong should my anchor be for estimating group and individual level meaningful change? A simulation study assessing anchor correlation strength and the impact of sample size, distribution of change scores and methodology on establishing a true meaningful change threshold. Qual Life Res 32(5):1255–1264. 10.1007/s11136-022-03286-w 36401757 10.1007/s11136-022-03286-w

[27] Mamolo CM, Bushmakin AG, Cappelleri JC (2015) Application of the itch severity score in patients with moderate-to-severe plaque psoriasis: clinically important difference and responder analyses. J Dermatological Treat 26(2):121–123 10.3109/09546634.2014.906033 24716586

[28] Froud R, Abel G (2014) Using ROC curves to choose minimally important change thresholds when sensitivity and specificity are valued equally: the forgotten lesson of pythagoras. theoretical considerations and an example application of change in health status. PLoS ONE 9(12):e114468 25474472 10.1371/journal.pone.0114468 PMC4256421

[29] Food and Drug Administration (2019) Incorporating clinical outcome assessments into endpoints for regulatory decision-making

[30] Terwee CB, Peipert JD, Chapman R, Lai J-S, Terluin B, Cella D, Griffith P, Mokkink LB (2021) Minimal important change (MIC): a conceptual clarification and systematic review of MIC estimates of PROMIS measures. Qual Life Res 30(10):2729–2754 34247326 10.1007/s11136-021-02925-y PMC8481206

[31] Trigg A, Kelly M, Iadeluca L, Chang J, Moreno-Koehler A, Yaworsky A, Krohe M, Rider A, Cappelleri JC, Cella D (2021) Reliability, validity and important difference estimates for the NCCN-FACT ovarian symptom index-18 (NFOSI-18). Future Oncol 17(30):3951–3964 34287020 10.2217/fon-2021-0358

[32] Griffiths P, Peipert JD, Leith A, Rider A, Morgan L, Cella D, Cocks K (2022) Validity of a single-item indicator of treatment side effect bother in a diverse sample of cancer patients. Support Care Cancer 30(4):3613–3623 35031830 10.1007/s00520-022-06802-3 PMC8857159

[33] Coon CD, Cook KF (2018) Moving from significance to real-world meaning: methods for interpreting change in clinical outcome assessment scores. Qual Life Res 27(1):33–40 28620874 10.1007/s11136-017-1616-3

[34] Revicki DA, Cella D, Hays RD, Sloan JA, Lenderking WR, Aaronson NK (2006) Responsiveness and minimal important differences for patient reported outcomes. Health Qual Life Outcomes 4(1):1–5 17005038 10.1186/1477-7525-4-70 PMC1586195

[35] Trigg A, Griffiths P (2021) Triangulation of multiple meaningful change thresholds for patient-reported outcome scores. Qual Life Res 30(10):2755–2764 34319532 10.1007/s11136-021-02957-4

[36] Peipert JD, Hays RD, Cella D (2023) Likely change indexes improve estimates of individual change on patient-reported outcomes. Qual Life Res 32(5):1341–1352. 10.1007/s11136-022-03200-4 35921034 10.1007/s11136-022-03200-4 PMC9994541

[37] O’Connell N, Zhao F, Lee J-W, Hong F, Shen S-E, Ip E, Salem W, Peipert J, Graham N, Smith ML (2021) Low and moderate grade adverse events are important contributors to patient-reported treatment side-effect bother. In: Quality of life research, vol suppl 1. Springer Van Godewijckstraat 30, 3311 GZ Dordrecht, Netherlands, pp S82–S82

[38] Peipert JD, Zhao F, Lee J-W, Hong F, Ip E, Gareen I, Carlos R, Mayer I, Miller K (2020) Partridge A analysis of ECOG-ACRIN clinical trials to advance longitudinal assessment of cancer treatment tolerability. In: Quality of life research, Vol suppl 1. Springer Van Godewijckstraat 30, 3311 GZ Dordrecht, Netherlands, pp S13–S13

[39] Wagner LI, Zhao F, Goss PE, Chapman J-AW, Shepherd LE, Whelan TJ, Mattar BI, Bufill JA, Schultz WC, LaFrancis IE (2018) Patient-reported predictors of early treatment discontinuation: treatment-related symptoms and health-related quality of life among postmenopausal women with primary breast cancer randomized to anastrozole or exemestane on NCIC Clinical Trials Group (CCTG) MA. 27 (E1Z03). Breast Cancer Res Treat 169(3):537–548 29455298 10.1007/s10549-018-4713-2 PMC6092930

[40] Food and Drug Administration (2022) Core patient-reported outcomes in cancer clinical trials: guidance for industry

[41] Norman GR, Sloan JA, Wyrwich KW (2003) Interpretation of changes in health-related quality of life: the remarkable universality of half a standard deviation. Med Care 582–592 10.1097/01.MLR.0000062554.74615.4C 12719681

[42] Robertson JF, Di Leo A, Johnston S, Chia S, Bliss JM, Paridaens RJ, Lichfield J, Bradbury I, Campbell C (2021) Meta-analyses of visceral versus non-visceral metastatic hormone receptor-positive breast cancer treated by endocrine monotherapies. NPJ Breast Cancer 7(1):1–12 33579962 10.1038/s41523-021-00222-y PMC7881093

