# Supplementary material for: Validity, reliability, responsiveness, and clinically meaningful change threshold estimates of the National Comprehensive Cancer Network-Functional Assessment of Cancer Therapy-Breast Cancer Symptom Index (NFBSI-16)
Source: J Patient Rep Outcomes. 2024 Aug 15;8:97. doi: 10.1186/s41687-024-00776-y (PMC11327234; doi:10.1186/s41687-024-00776-y)
Supplement: Supplementary file 1 — Supplementary Material 1 [file 41687_2024_776_MOESM1_ESM.pdf]

Table of Contents

Figure 1.1.1.1	Empirical Cumulative Distribution Function of Change from Baseline to Cycle 5 in NFBSI-16 Total score by EQ-5D-5L VAS: with merged minimal and moderate categories	1
Figure 1.1.2.1	Empirical Cumulative Distribution Function of Change from Baseline to Cycle 7 in NFBSI-16 Total score by EQ-5D-5L VAS: with merged minimal and moderate categories	2
Figure 1.1.3.1	Empirical Cumulative Distribution Function of Change from Baseline to Cycle 9 in NFBSI-16 Total score by EQ-5D-5L VAS: with merged minimal and moderate categories	3
Figure 1.2.1.1	Empirical Cumulative Distribution Function of Change from Baseline to Cycle 5 in NFBSI-16 DRS-P by EQ-5D-5L Pain: with merged minimal and moderate categories	4
Figure 1.2.1.2	Empirical Cumulative Distribution Function of Change from Baseline to Cycle 5 in NFBSI-16 DRS-P by EQ-5D-5L Usual Activities merged minimal and moderate categories	5
Figure 1.2.2.1	Empirical Cumulative Distribution Function of Change from Baseline to Cycle 7 in NFBSI-16 DRS-P by EQ-5D-5L Pain: with merged minimal and moderate categories	6
Figure 1.2.2.2	Empirical Cumulative Distribution Function of Change from Baseline to Cycle 7 in NFBSI-16 DRS-P by EQ-5D-5L Usual Activities	7
Figure 1.2.3.1	Empirical Cumulative Distribution Function of Change from Baseline to Cycle 9 in NFBSI-16 DRS-P by EQ-5D-5L Pain: with merged minimal and moderate categories	8
Figure 1.2.3.2	Empirical Cumulative Distribution Function of Change from Baseline to Cycle 9 in NFBSI-16 DRS-P by EQ-5D-5L Usual Activities with merged minimal and moderate categories	9
Figure 1.3.1.2	Empirical Cumulative Distribution Function of Change from Baseline to Cycle 5 in NFBSI-16 DRS-E by EQ-5D-5L VAS: with merged minimal and moderate categories	10
Figure 1.3.2.2	Empirical Cumulative Distribution Function of Change from Baseline to Cycle 7 in NFBSI-16 DRS-E by EQ-5D-5L VAS: with merged minimal and moderate categorie	11
Figure 1.3.3.2	Empirical Cumulative Distribution Function of Change from Baseline to Cycle 9 in NFBSI-16 DRS-E by EQ-5D-5L VAS: with merged minimal and moderate categories	12
Figure 2.1.1.1	ROC curve for NFBSI-16 Total score, Improved versus Stable, according to EQ-5D-5L VAS from Baseline to Cycle 5	13
Figure 2.1.2.1	ROC curve for NFBSI-16 Total score, Improved versus Stable, according to EQ-5D-5L VAS from Baseline to Cycle 7:	14
Figure 2.1.3.1	ROC curve for NFBSI-16 Total score, Improved versus Stable, according to EQ-5D-5L VAS from Baseline to Cycle 9	15
Figure 2.2.1.1	ROC curve for NFBSI-16 DRS-P, Improved versus Stable, according to EQ-5D-5L Pain from Baseline to Cycle 5	16
Figure 2.2.1.2	ROC curve for NFBSI-16 DRS-P, Improved versus Stable, according to EQ-5D-5L Usual Activities from Baseline to Cycle 5	17
Figure 2.2.2.1	ROC curve for NFBSI-16 DRS-P, Improved versus Stable, according to EQ-5D-5L Pain from Baseline to Cycle 7	18
Figure 2.2.2.2	ROC curve for NFBSI-16 DRS-P, Improved versus Stable, according to EQ-5D-5L Usual Activities from Baseline to Cycle 7	19
Figure 2.2.3.1	ROC curve for NFBSI-16 DRS-P, Improved versus Stable, according to EQ-5D-5L Pain from Baseline to Cycle 9	20
Figure 2.2.3.2	ROC curve for NFBSI-16 DRS-P, Improved versus Stable, according to EQ-5D-5L Usual Activities from Baseline to Cycle 9	21
Figure 2.3.1.2	ROC curve for NFBSI-16 DRS-E, Improved versus Stable, according to EQ-5D-5L VAS from Baseline to Cycle 5	22
Figure 2.3.2.2	ROC curve for NFBSI-16 DRS-E, Improved versus Stable, according to EQ-5D-5L VAS from Baseline to Cycle 7	23
Figure 2.3.3.2	ROC curve for NFBSI-16 DRS-E, Improved versus Stable, according to EQ-5D-5L VAS from Baseline to Cycle 9	24
Figure 3.1.1.1	ROC curve for NFBSI-16 Total score, Worsened versus Stable, according to EQ-5D-5L VAS from Baseline to Cycle 5	25
Figure 3.1.2.1	ROC curve for NFBSI-16 Total score, Worsened versus Stable, according to EQ-5D-5L VAS from Baseline to Cycle 7	26
Figure 3.1.3.1	ROC curve for NFBSI-16 Total score, Worsened versus Stable, according to EQ-5D-5L VAS from Baseline to Cycle 9	27
Figure 3.2.1.1	ROC curve for NFBSI-16 DRS-P, Worsened versus Stable, according to EQ-5D-5L Pain from Baseline to Cycle 5	28
Figure 3.2.1.2	ROC curve for NFBSI-16 DRS-P, Worsened versus Stable, according to EQ-5D-5L Usual Activities from Baseline to Cycle 5	29
Figure 3.2.2.1	ROC curve for NFBSI-16 DRS-P, Worsened versus Stable, according to EQ-5D-5L Pain from Baseline to Cycle 7	30
Figure 3.2.2.2	ROC curve for NFBSI-16 DRS-P, Worsened versus Stable, according to EQ-5D-5L Usual Activities from Baseline to Cycle 7	31
Figure 3.2.3.1	ROC curve for NFBSI-16 DRS-P, Worsened versus Stable, according to EQ-5D-5L Pain from Baseline to Cycle 9	32
Figure 3.2.3.2	ROC curve for NFBSI-16 DRS-P, Worsened versus Stable, according to EQ-5D-5L Usual Activities from Baseline to Cycle 9	33
Figure 3.3.1.2	ROC curve for NFBSI-16 DRS-E, Worsened versus Stable, according to EQ-5D-5L VAS from Baseline to Cycle 5	34
Figure 3.3.2.2	ROC curve for NFBSI-16 DRS-E, Worsened versus Stable, according to EQ-5D-5L VAS from Baseline to Cycle 7	35
Figure 3.3.3.2	ROC curve for NFBSI-16 DRS-E, Worsened versus Stable, according to EQ-5D-5L VAS from Baseline to Cycle 9	36

Figure 1.1.1.1: Empirical Cumulative Distribution Function of Change from Baseline to Cycle 5 in NFBSI-16 Total score by EQ-5D-5L VAS with merged minimal and moderate categories

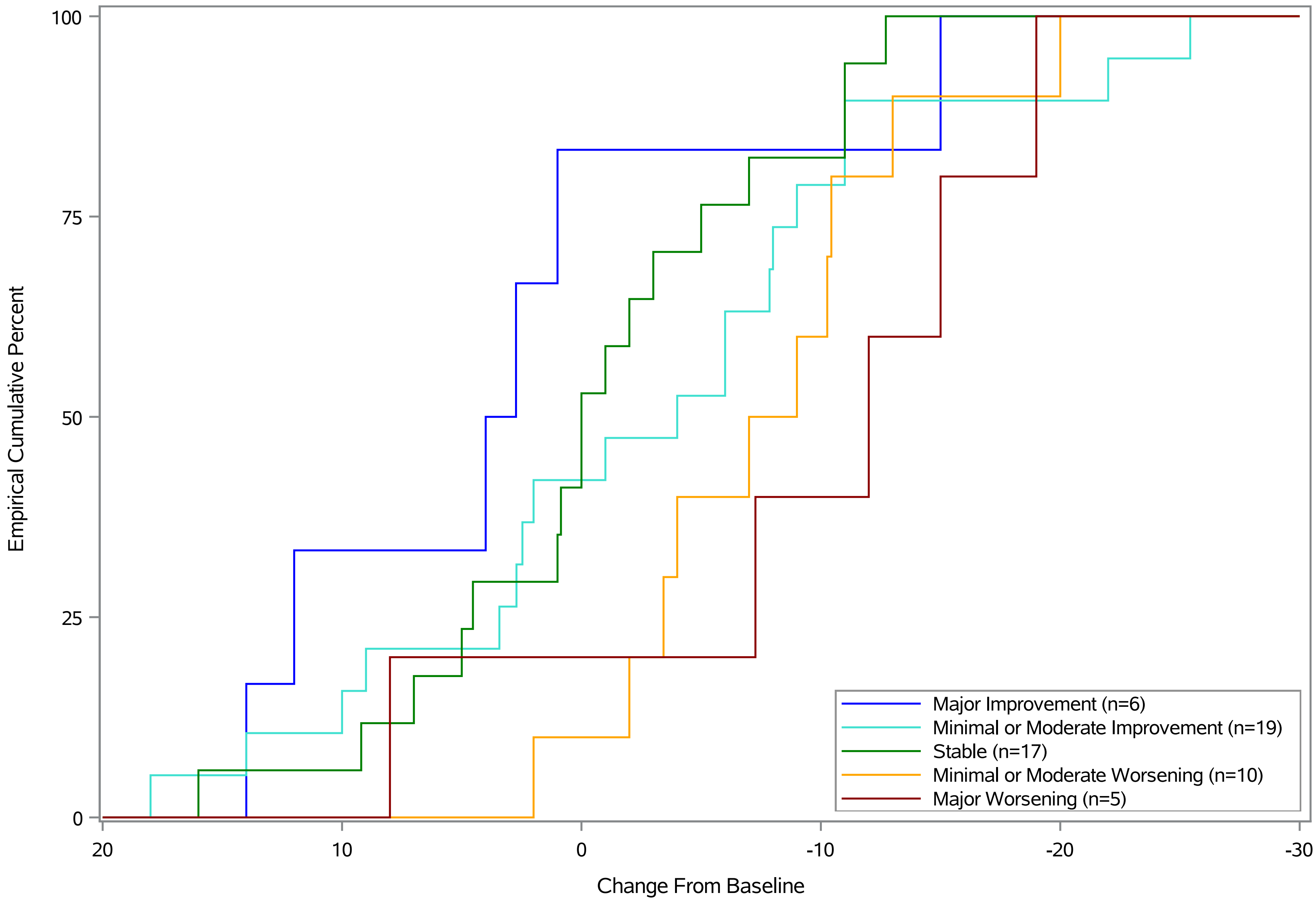

NFBSI: National Comprehensive Cancer Network Functional Assessment Of Cancer Therapy-Breast Cancer Symptom Index; EQ-5D-5L VAS Anchor

Figure 1.1.2.1: Empirical Cumulative Distribution Function of Change from Baseline to Cycle 7 in NFBSI-16 Total score by EQ-5D-5L VAS with merged minimal and moderate categories

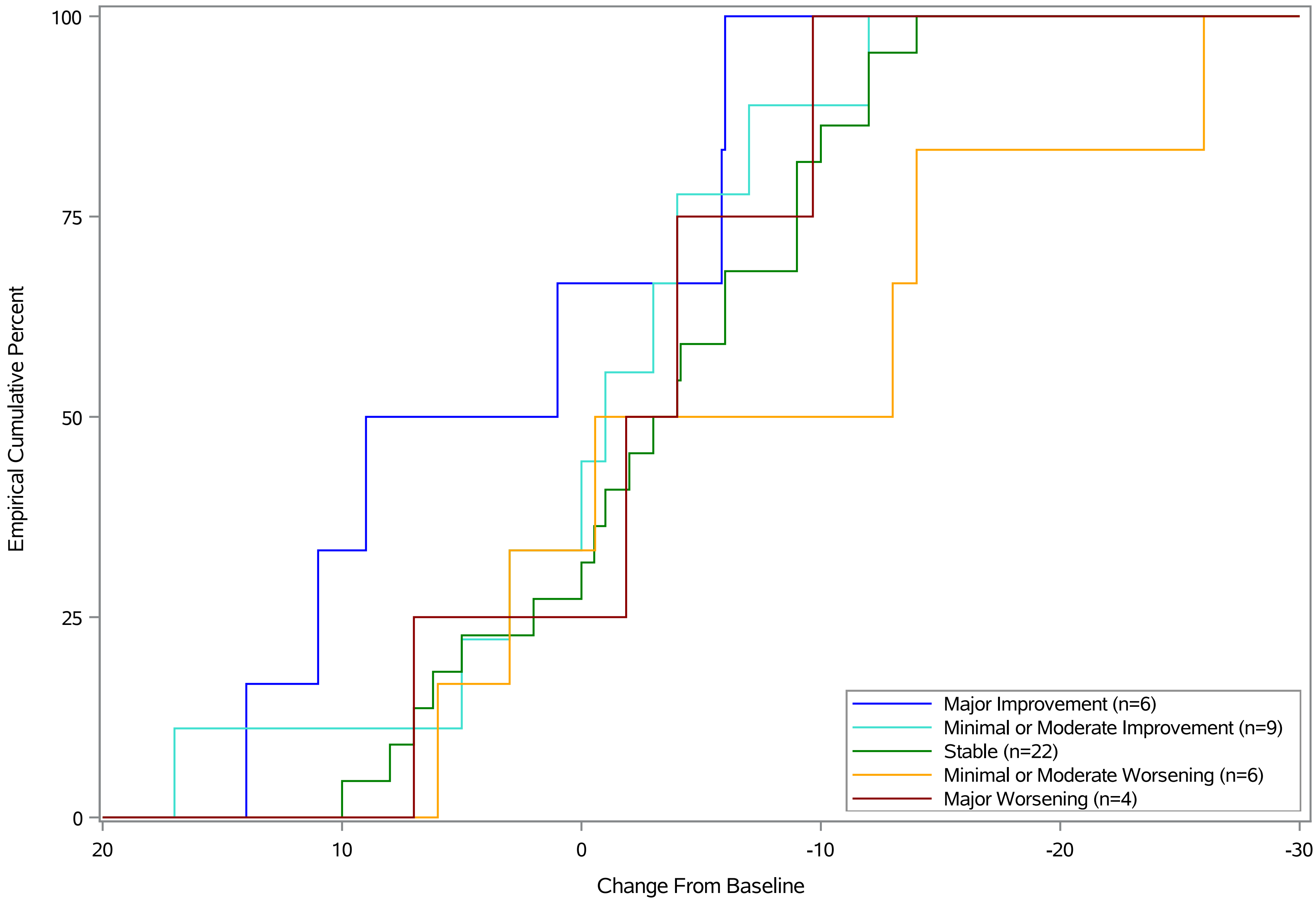

NFBSI: National Comprehensive Cancer Network Functional Assessment Of Cancer Therapy-Breast Cancer Symptom Index; EQ-5D-5L VAS Anchor

Figure 1.1.3.1: Empirical Cumulative Distribution Function of Change from Baseline to Cycle 9 in NFBSI-16 Total score by EQ-5D-5L VAS with merged minimal and moderate categories

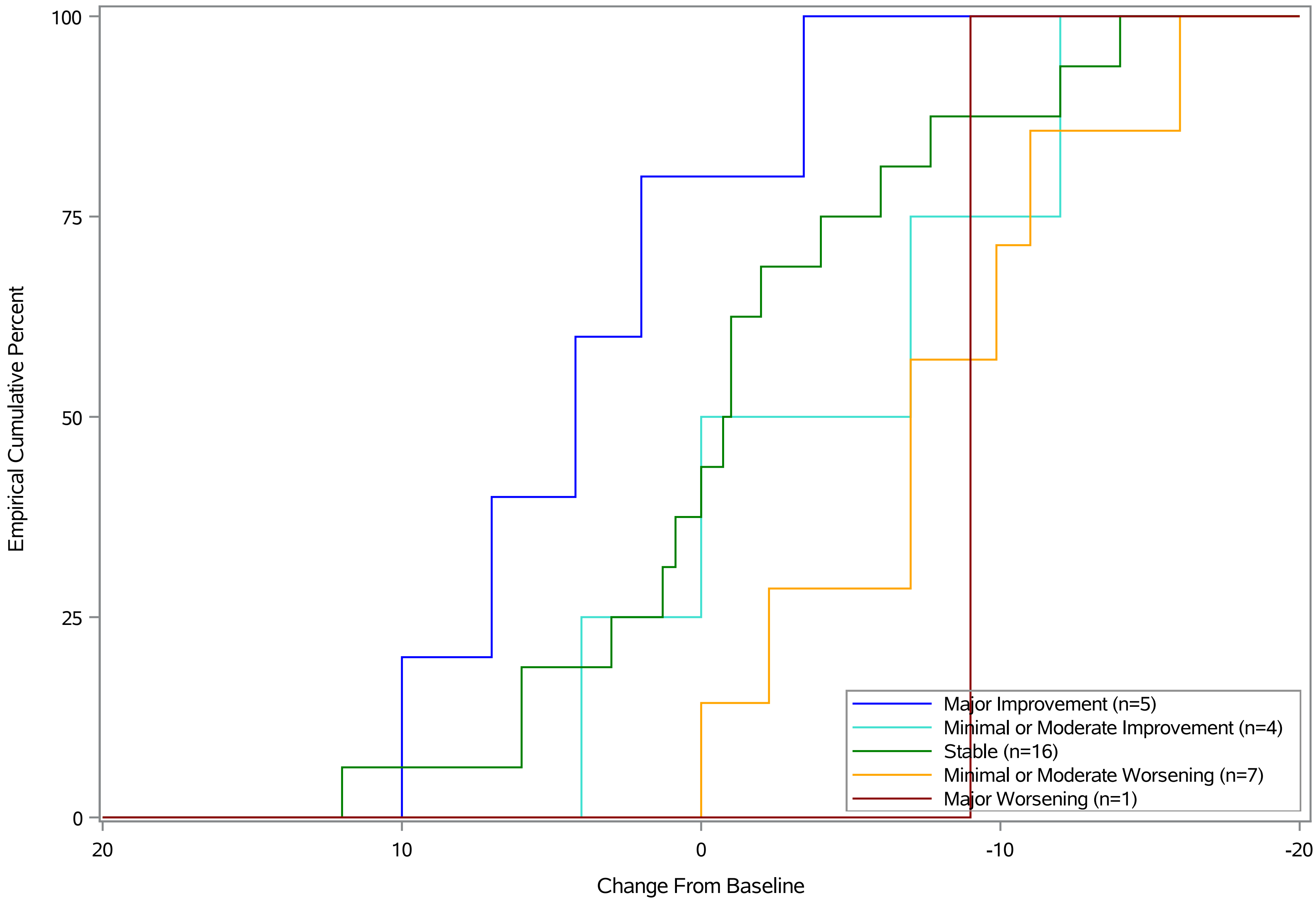

NFBSI: National Comprehensive Cancer Network Functional Assessment Of Cancer Therapy-Breast Cancer Symptom Index; EQ-5D-5L VAS Anchor

Figure 1.2.1.1: Empirical Cumulative Distribution Function of Change from Baseline to Cycle 5 in NFBSI-16 DRS-P by EQ-5D-5L Pain with merged minimal and moderate categories

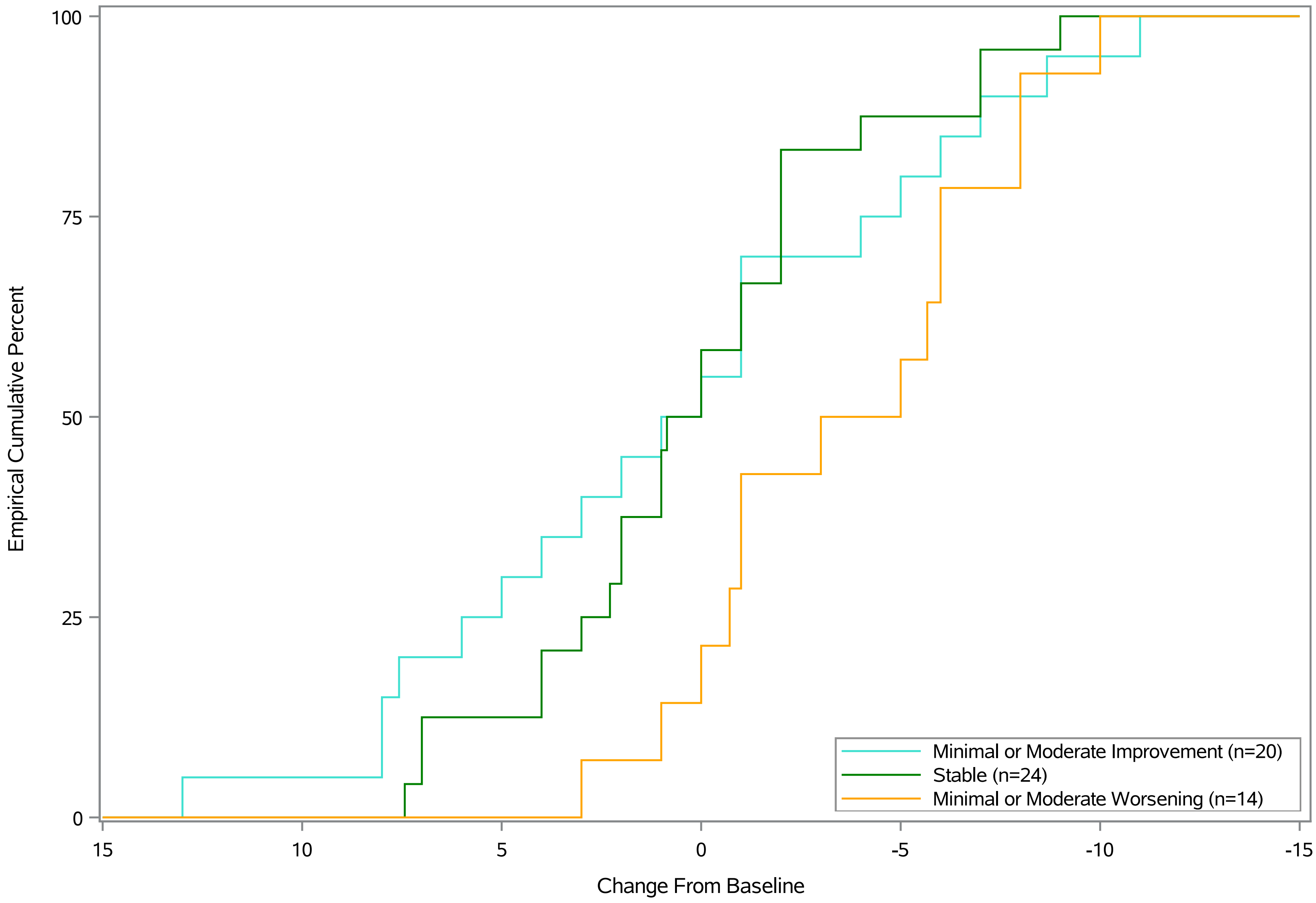

NFBSI: National Comprehensive Cancer Network Functional Assessment Of Cancer Therapy-Breast Cancer Symptom Index; DRSP: Disease-Related Symptoms Physical; EQ-5D-5L Pain Anchor

Figure 1.2.1.2: Empirical Cumulative Distribution Function of Change from Baseline to Cycle 5 in NFBSI-16 DRS-P by EQ-5D-5L Usual Activities with merged minimal and moderate categories

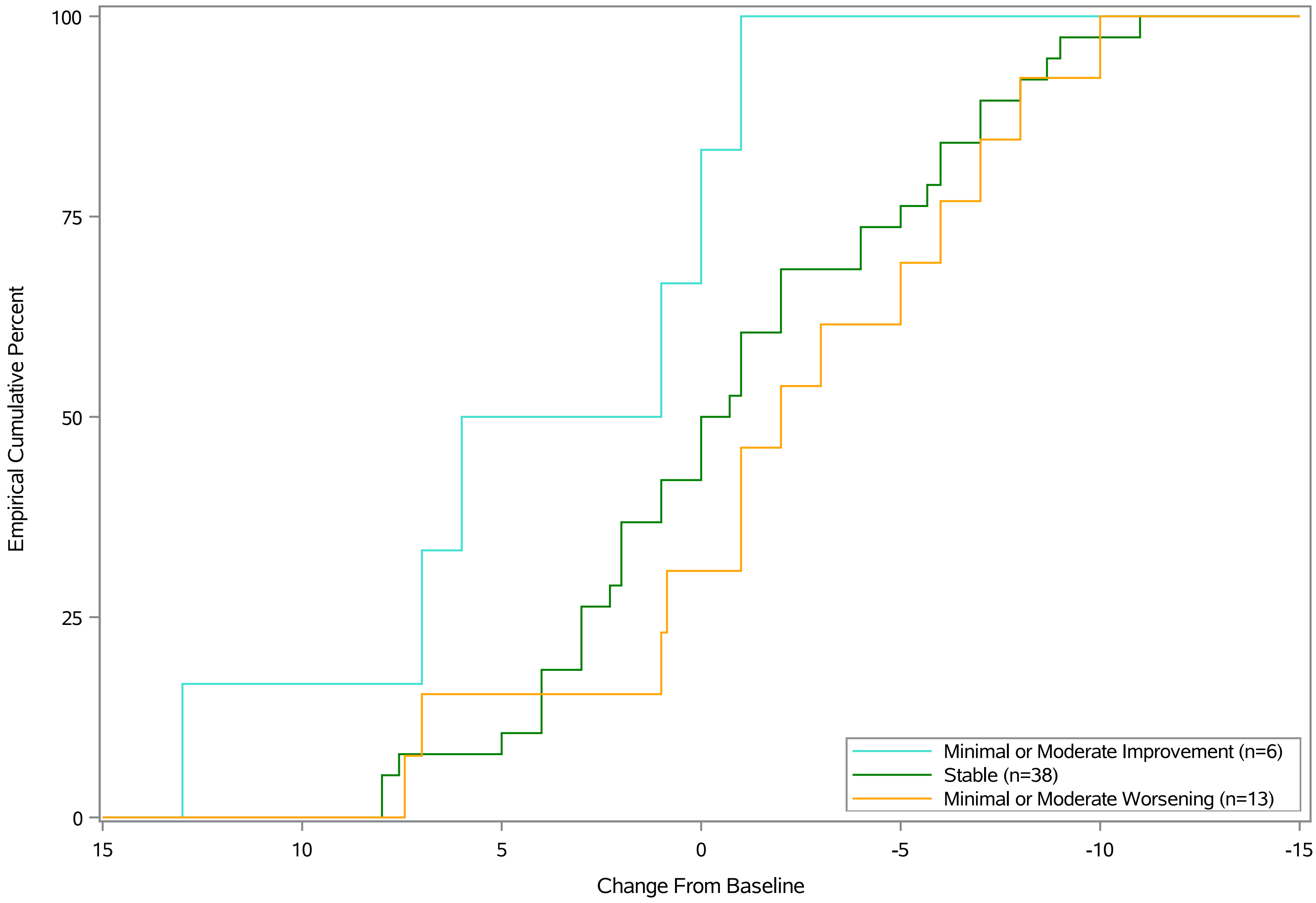

NFBSI: National Comprehensive Cancer Network Functional Assessment Of Cancer Therapy-Breast Cancer Symptom Index; DRSP: Disease-Related Symptoms Physical; EQ-5D-5L Usual Activities Anchor

Figure 1.2.2.1: Empirical Cumulative Distribution Function of Change from Baseline to Cycle 7 in NFBSI-16 DRS-P by EQ-5D-5L Pain with merged minimal and moderate categories

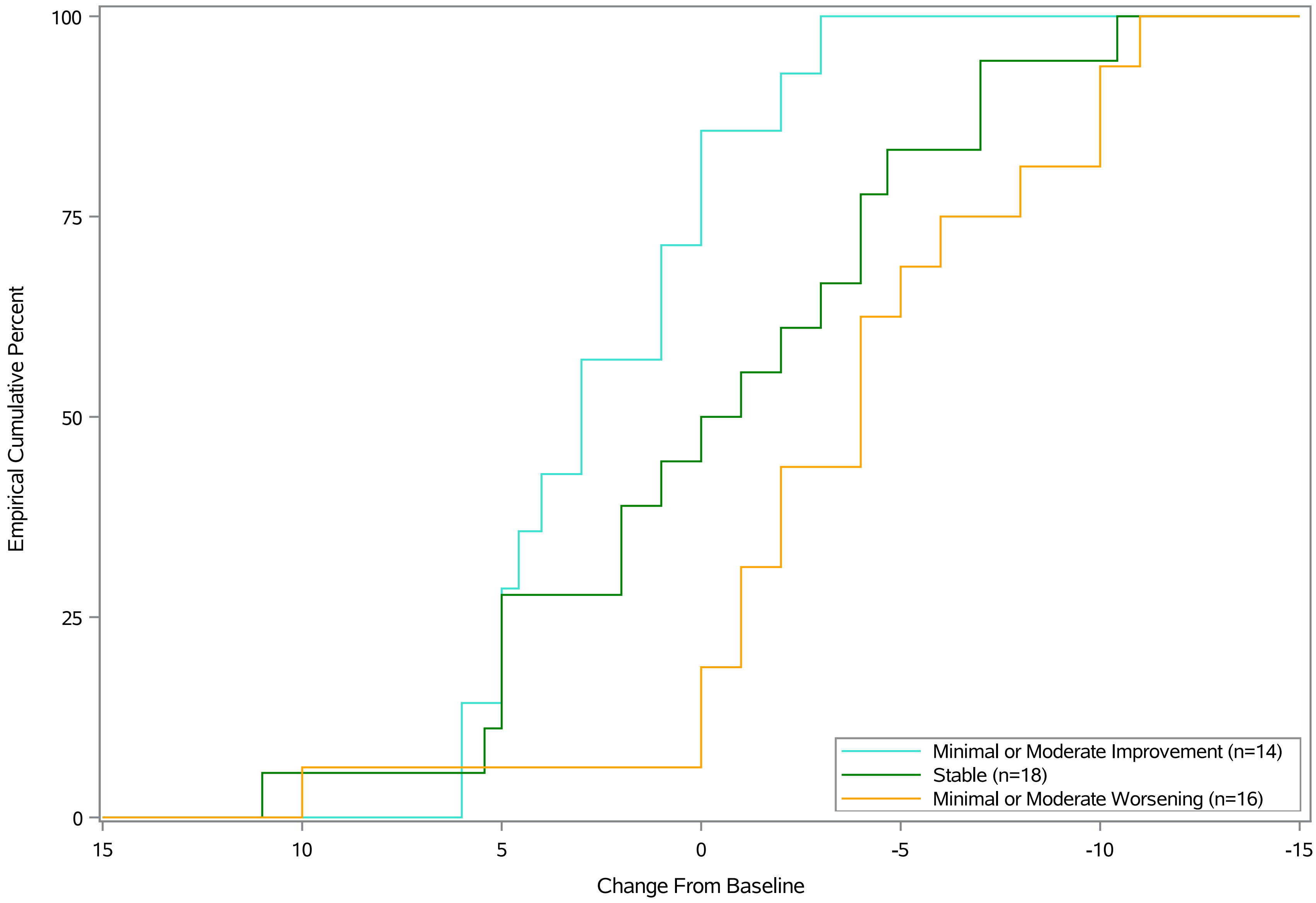

NFBSI: National Comprehensive Cancer Network Functional Assessment Of Cancer Therapy-Breast Cancer Symptom Index; DRSP: Disease-Related Symptoms Physical; EQ-5D-5L Pain Anchor

Figure 1.2.2.2: Empirical Cumulative Distribution Function of Change from Baseline to Cycle 7 in NFBSI-16 DRS-P by EQ-5D-5L Usual Activities with merged minimal and moderate categories

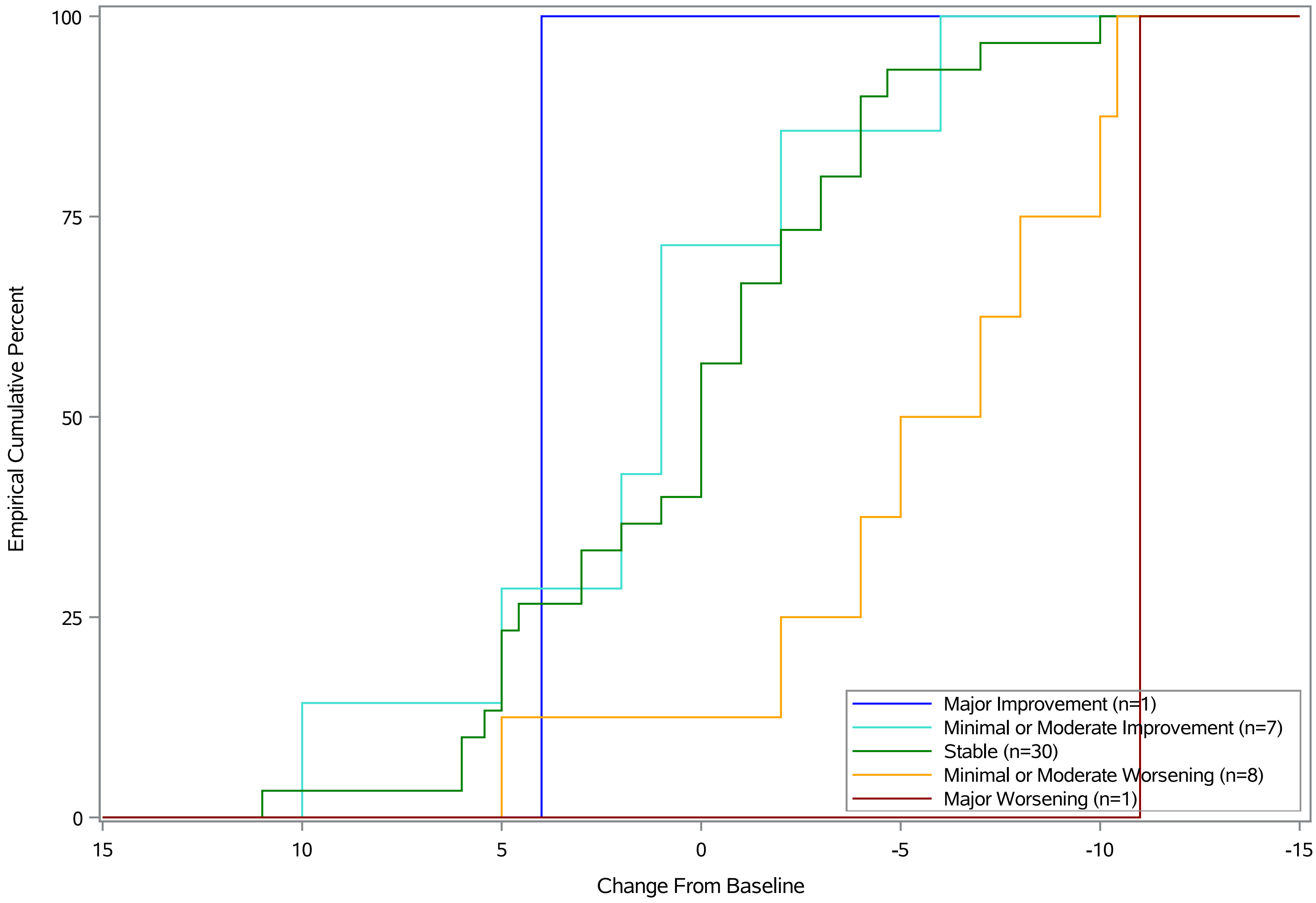

NFBSI: National Comprehensive Cancer Network Functional Assessment Of Cancer Therapy-Breast Cancer Symptom Index; DRSP: Disease-Related Symptoms Physical; EQ-5D-5L Usual Activities Anchor

Figure 1.2.3.1: Empirical Cumulative Distribution Function of Change from Baseline to Cycle 9 in NFBSI-16 DRS-P by EQ-5D-5L Pain with merged minimal and moderate categories

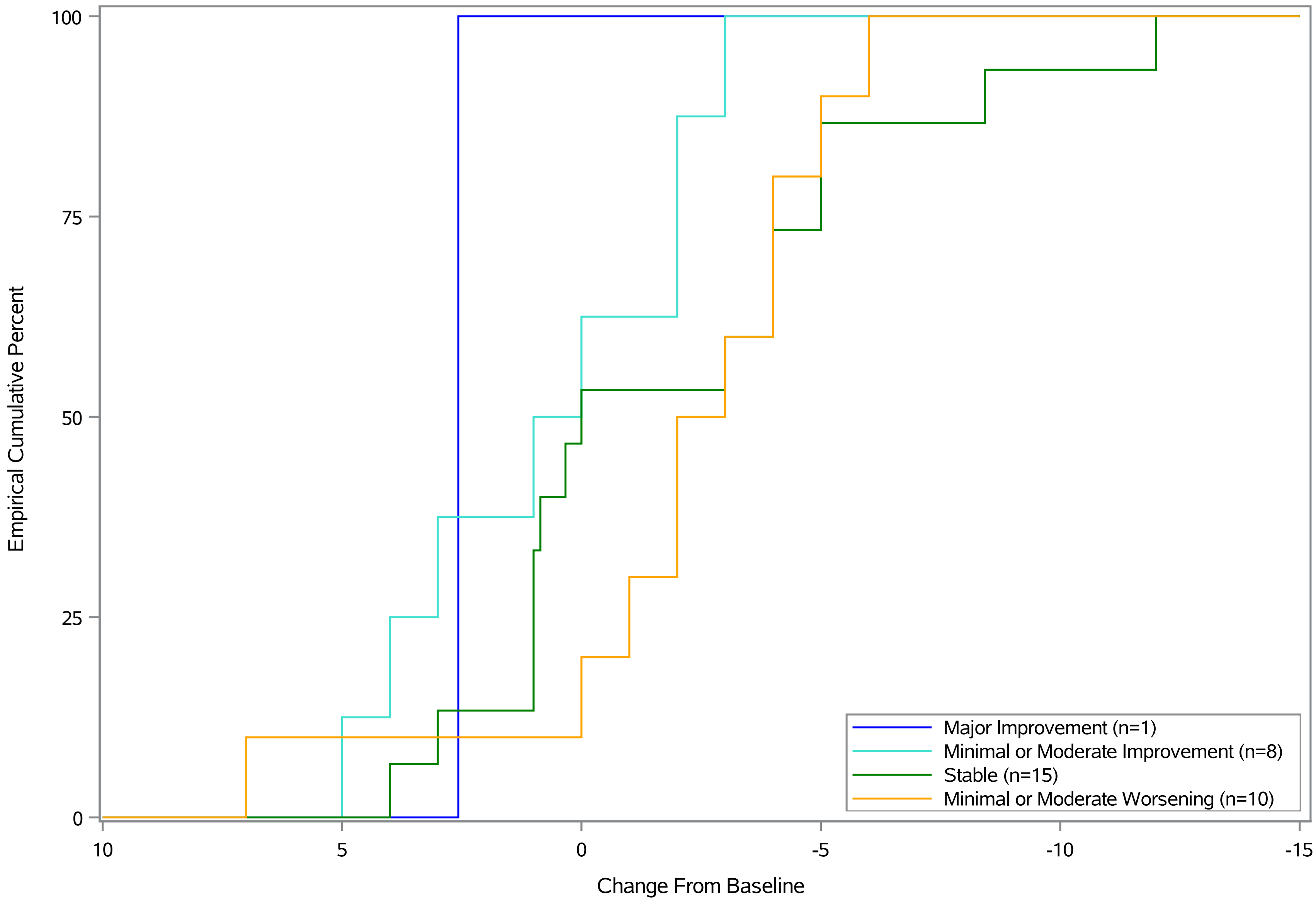

NFBSI: National Comprehensive Cancer Network Functional Assessment Of Cancer Therapy-Breast Cancer Symptom Index; DRSP: Disease-Related Symptoms Physical; EQ-5D-5L Pain Anchor

Figure 1.2.3.2: Empirical Cumulative Distribution Function of Change from Baseline to Cycle 9 in NFBSI-16 DRS-P by EQ-5D-5L Usual Activities with merged minimal and moderate categories

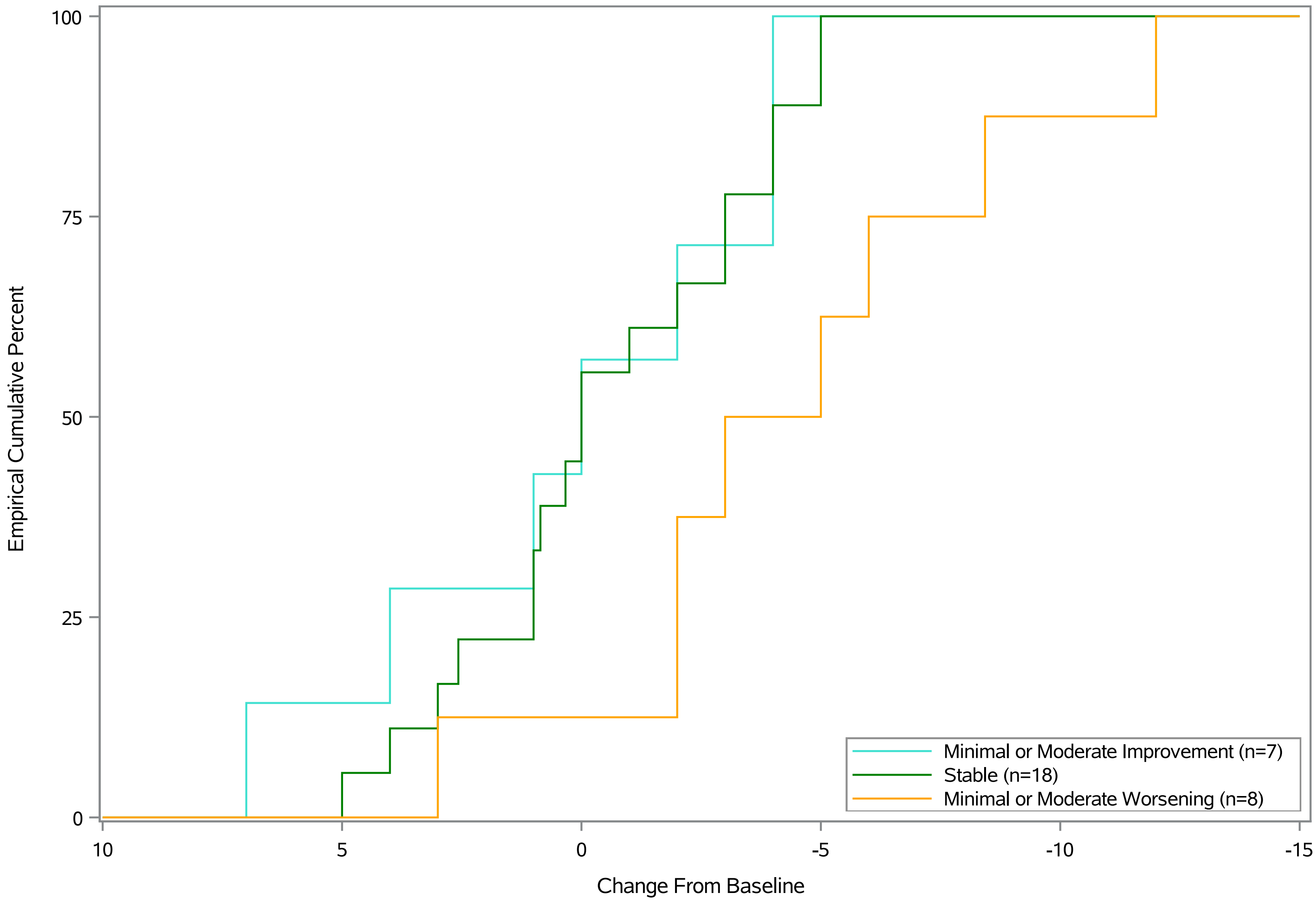

NFBSI: National Comprehensive Cancer Network Functional Assessment Of Cancer Therapy-Breast Cancer Symptom Index; DRSP: Disease-Related Symptoms Physical; EQ-5D-5L Usual Activities Anchor

Figure 1.3.1.2: Empirical Cumulative Distribution Function of Change from Baseline to Cycle 5 in NFBSI-16 DRS-E by EQ-5D-5L VAS with merged minimal and moderate categories

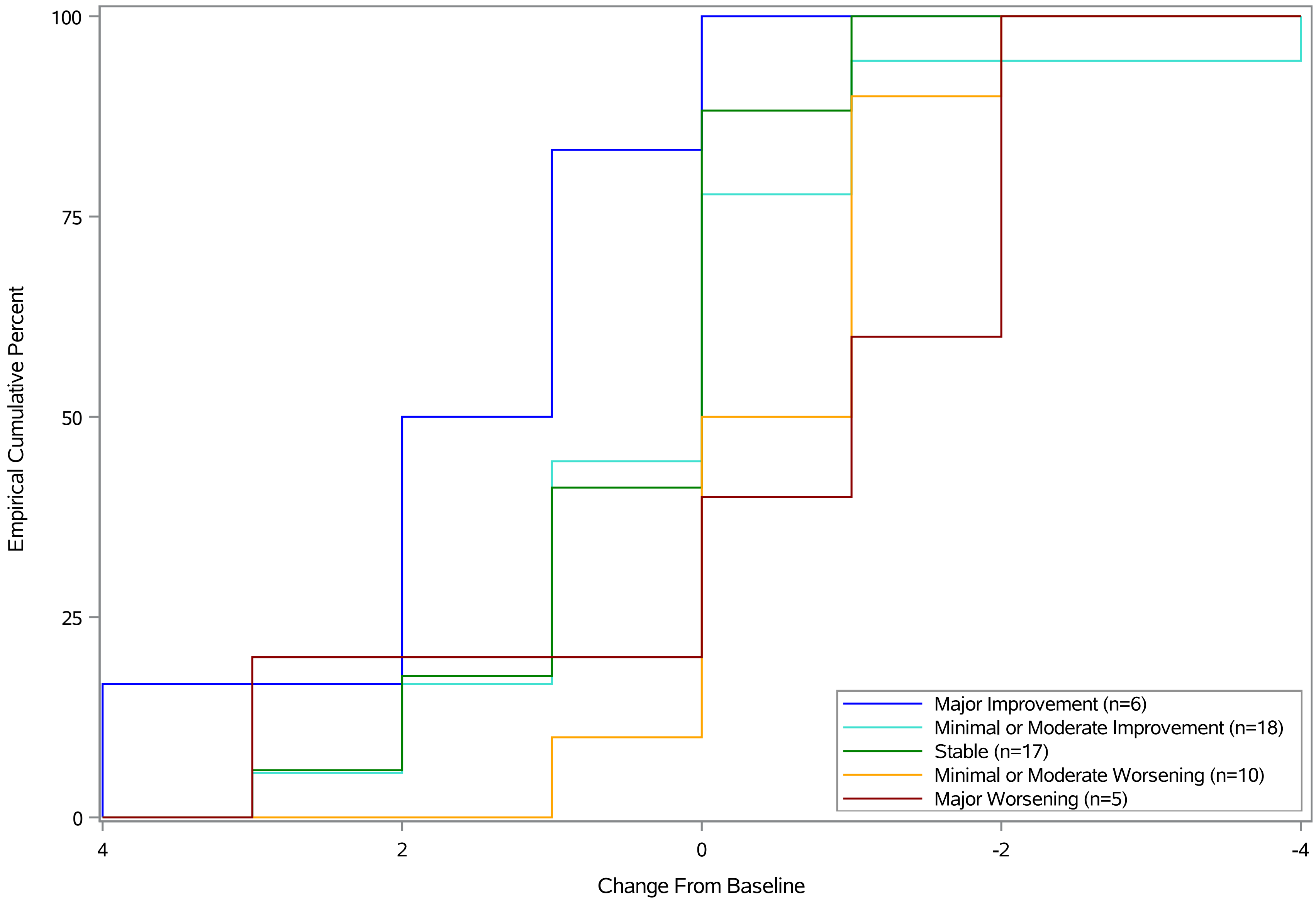

NFBSI: National Comprehensive Cancer Network Functional Assessment Of Cancer Therapy-Breast Cancer Symptom Index; DRSE: Disease-Related Symptoms Emotional; EQ-5D-5L VAS Anchor

Figure 1.3.2.2: Empirical Cumulative Distribution Function of Change from Baseline to Cycle 7 in NFBSI-16 DRS-E by EQ-5D-5L VAS with merged minimal and moderate categories

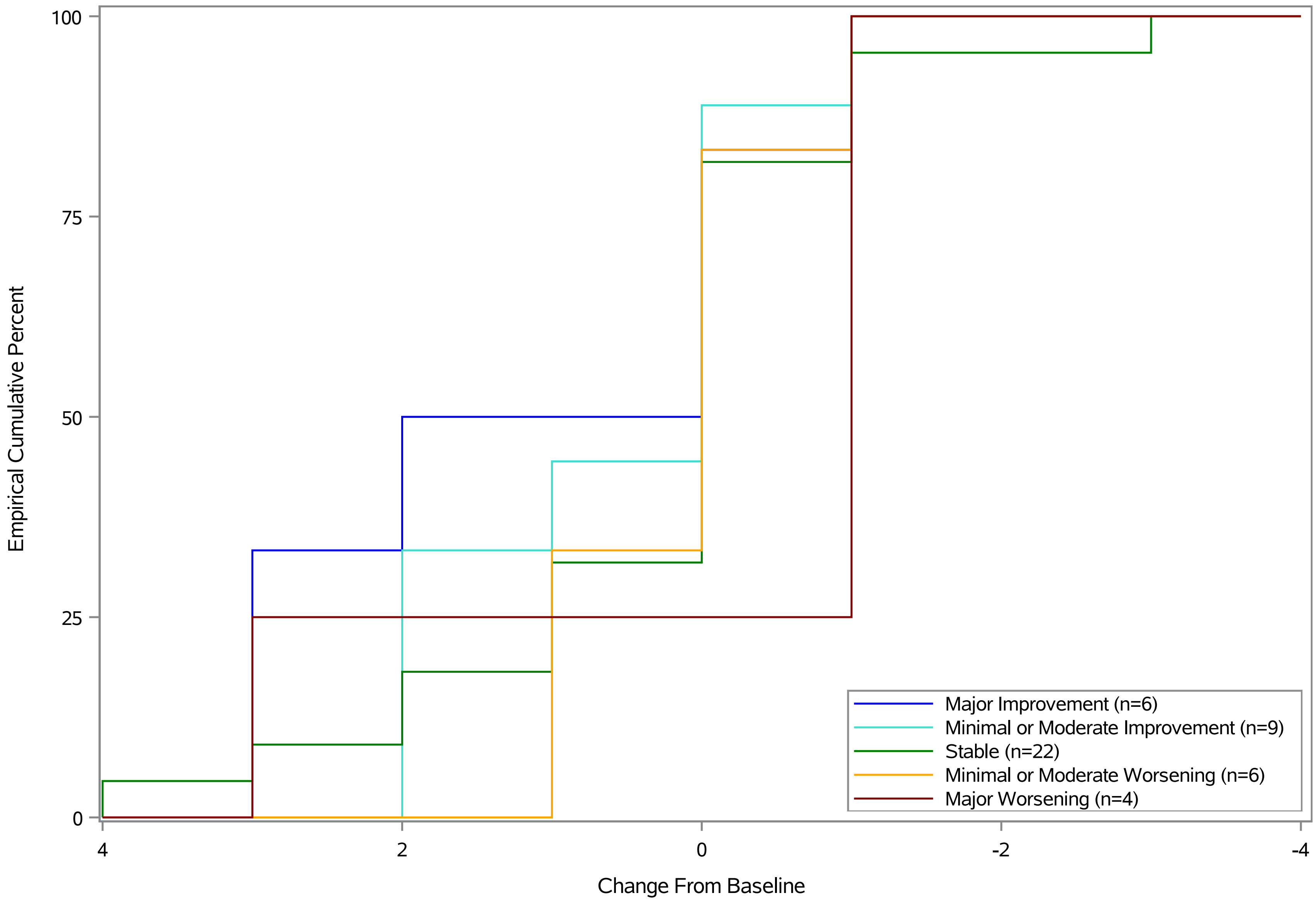

NFBSI: National Comprehensive Cancer Network Functional Assessment Of Cancer Therapy-Breast Cancer Symptom Index; DRSE: Disease-Related Symptoms Emotional; EQ-5D-5L VAS Anchor

Figure 1.3.3.2: Empirical Cumulative Distribution Function of Change from Baseline to Cycle 9 in NFBSI-16 DRS-E by EQ-5D-5L VAS with merged minimal and moderate categories

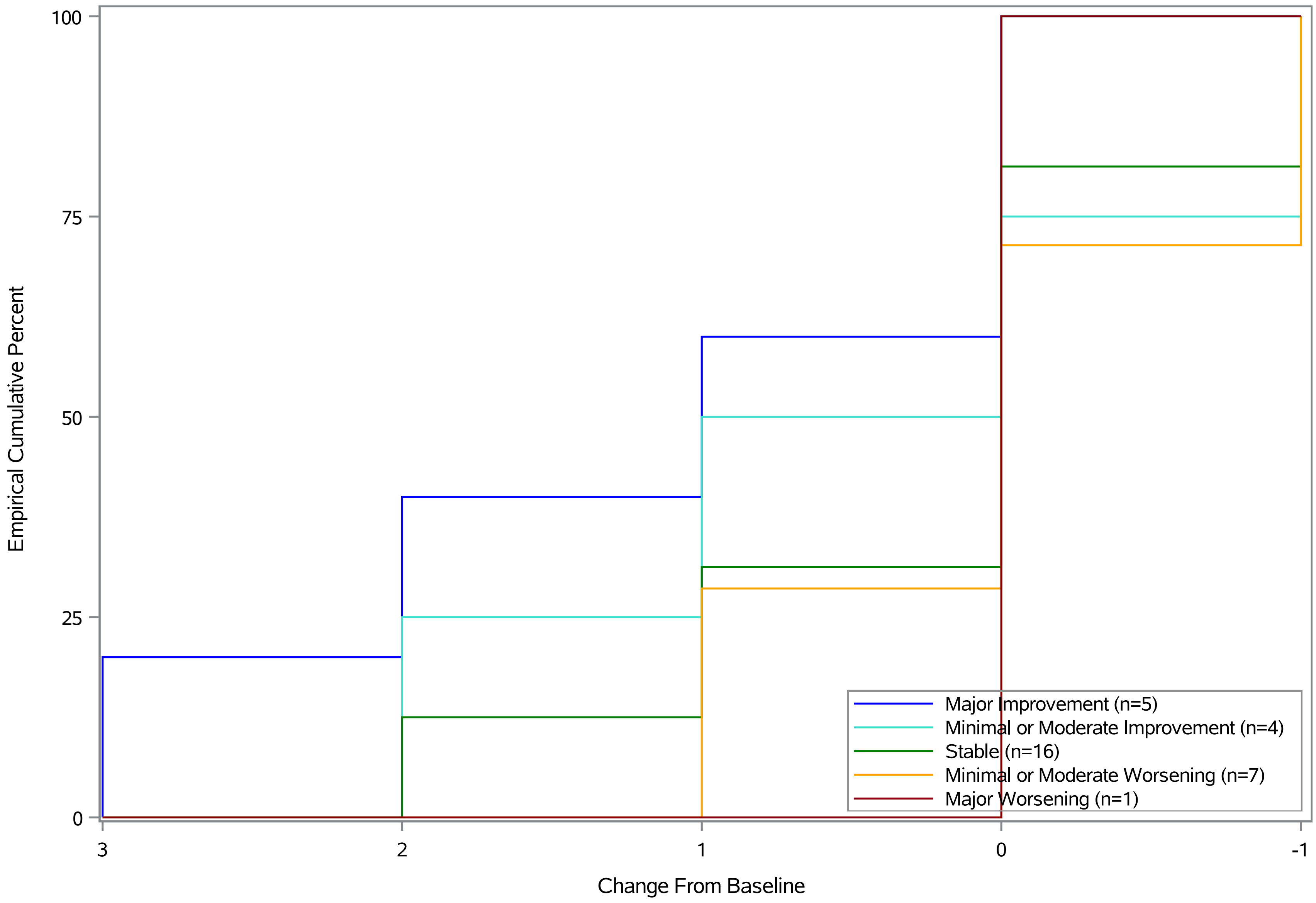

NFBSI: National Comprehensive Cancer Network Functional Assessment Of Cancer Therapy-Breast Cancer Symptom Index; DRSE: Disease-Related Symptoms Emotional; EQ-5D-5L VAS Anchor

Figure 2.1.1.1: ROC curve for NFBSI-16 Total score, Improved versus Stable, according to EQ-5D-5L VAS from Baseline to Cycle 5

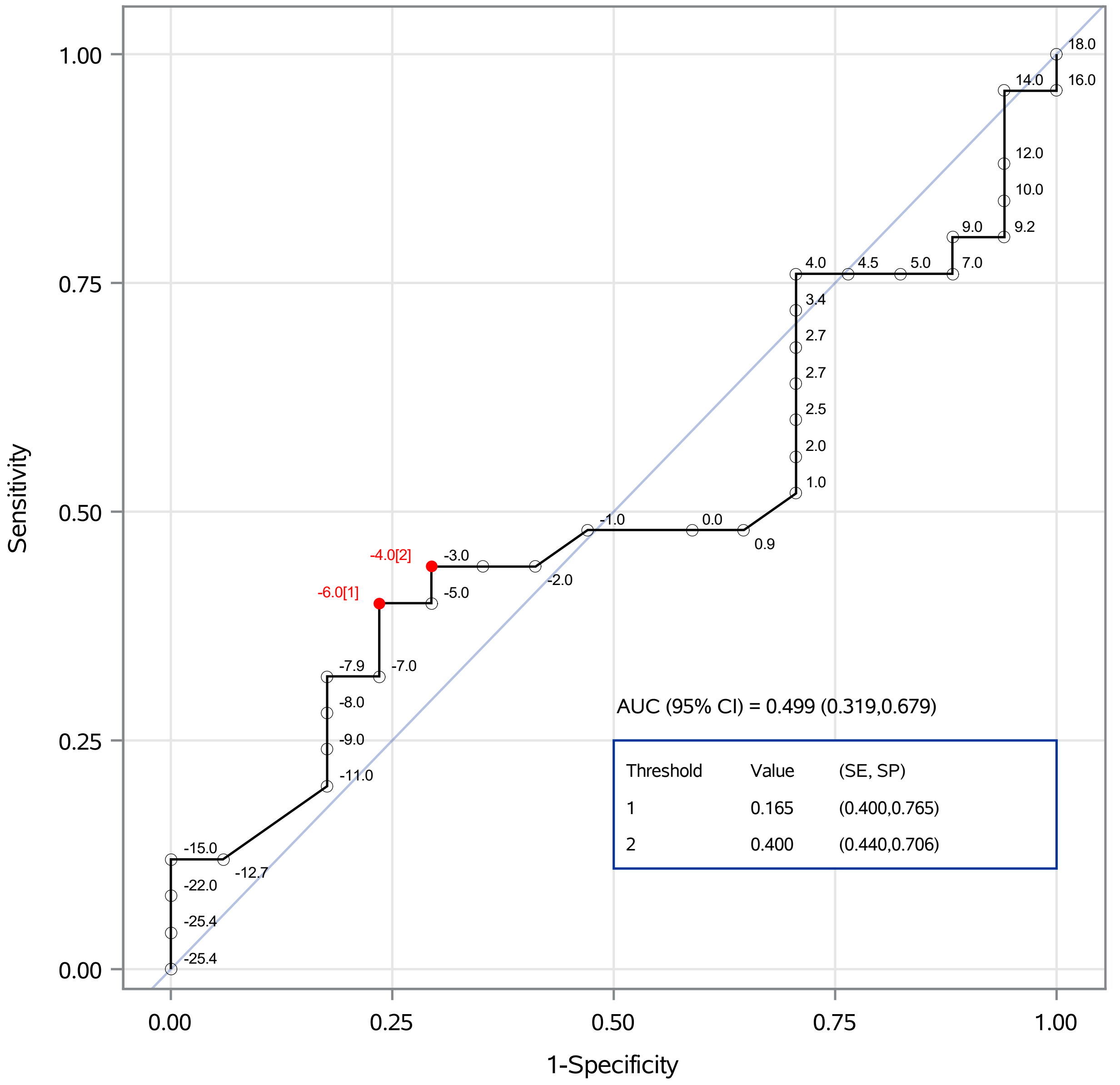

NFBSI: National Comprehensive Cancer Network Functional Assessment Of Cancer Therapy-Breast Cancer Symptom Index; IMPSTAB: 'Minimal/Moderate/Major Improvement' versus 'Stable' ; EQ-5D-5L VAS Anchor

Figure 2.1.2.1: ROC curve for NFBSI-16 Total score, Improved versus Stable, according to EQ-5D-5L VAS from Baseline to Cycle 7

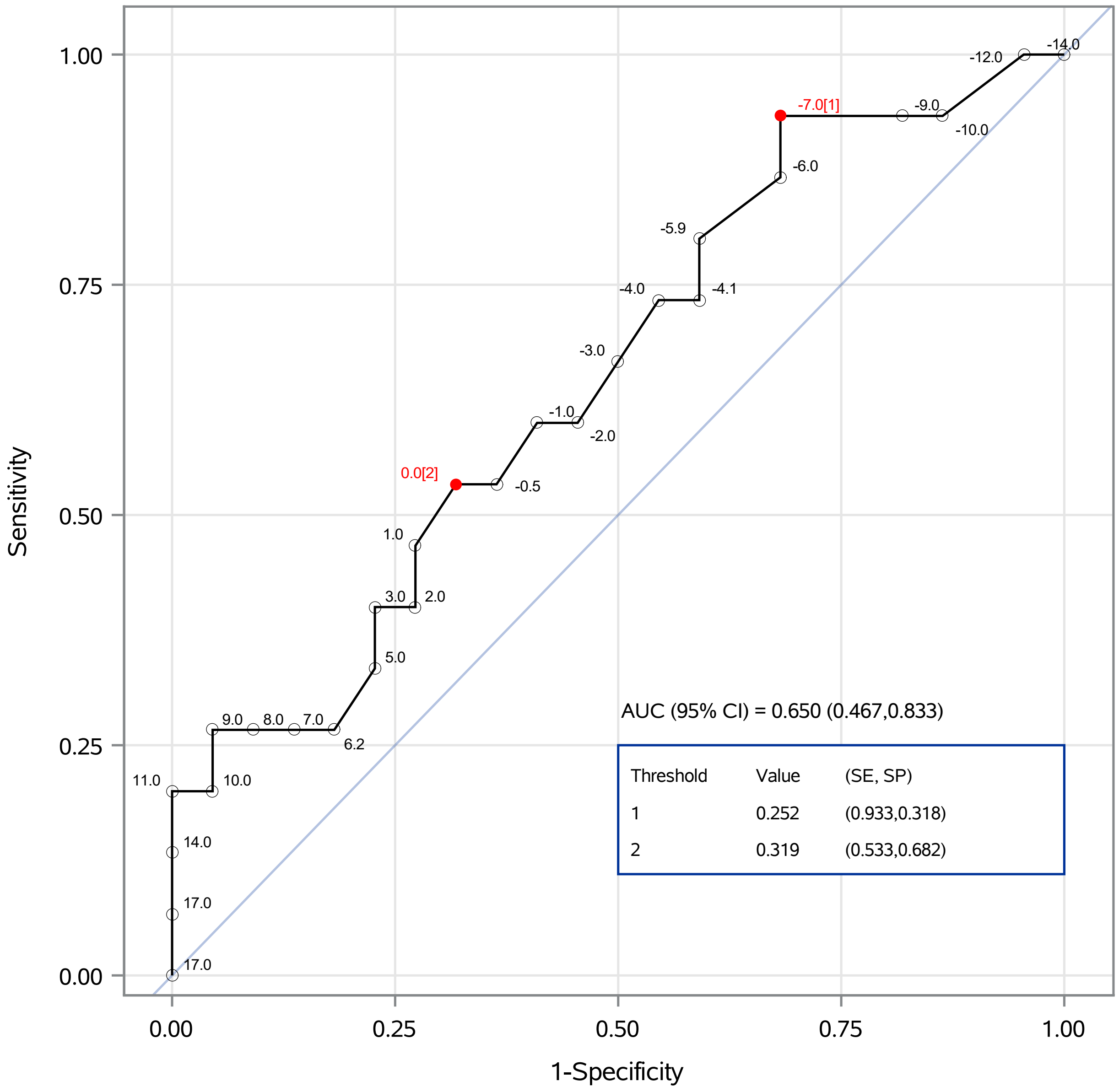

NFBSI: National Comprehensive Cancer Network Functional Assessment Of Cancer Therapy-Breast Cancer Symptom Index; IMPSTAB: 'Minimal/Moderate/Major Improvement' versus 'Stable' ; EQ-5D-5L VAS Anchor

Figure 2.1.3.1: ROC curve for NFBSI-16 Total score, Improved versus Stable, according to EQ-5D-5L VAS from Baseline to Cycle 9

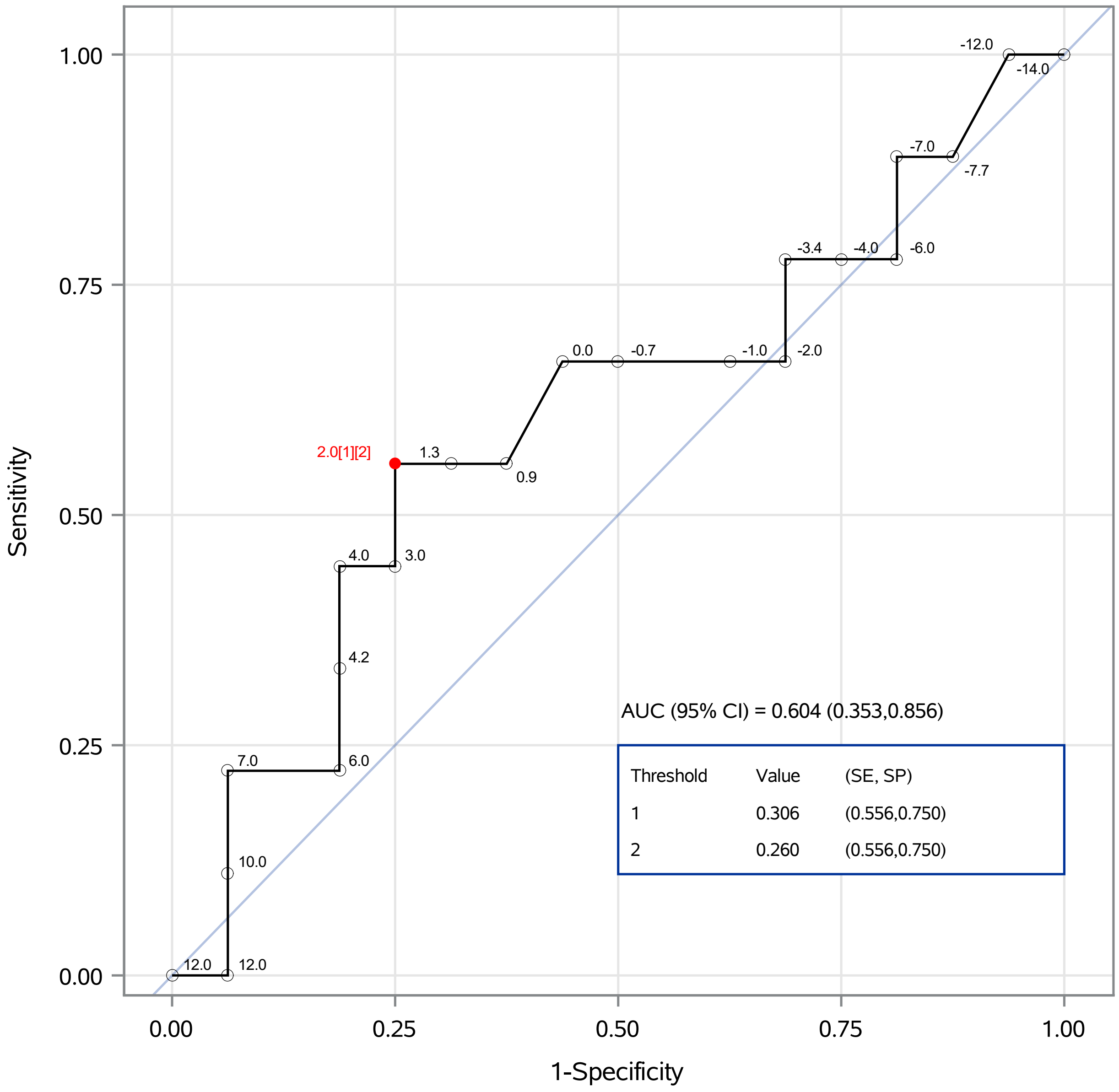

NFBSI: National Comprehensive Cancer Network Functional Assessment Of Cancer Therapy-Breast Cancer Symptom Index; IMPSTAB: 'Minimal/Moderate/Major Improvement' versus 'Stable' ; EQ-5D-5L VAS Anchor

Figure 2.2.1.1: ROC curve for NFBSI-16 DRS-P, Improved versus Stable, according to EQ-5D-5L Pain from Baseline to Cycle 5

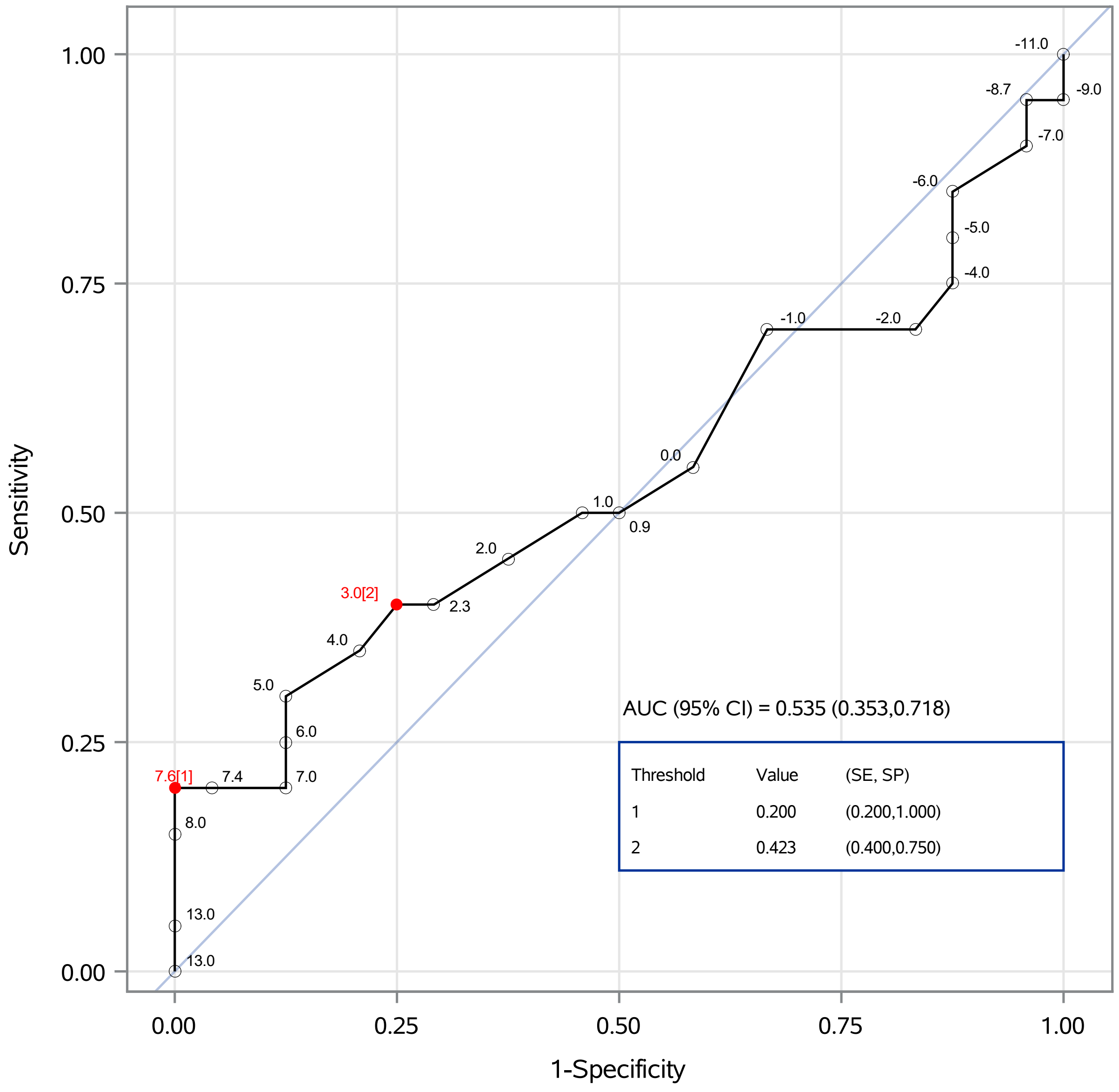

NFBSI: National Comprehensive Cancer Network Functional Assessment Of Cancer Therapy-Breast Cancer Symptom Index; DRSP: Disease-Related Symptoms Physical; IMPSTAB: 'Minimal/Moderate/Major Improvement' versus 'Stable' ; EQ-5D-5L Pain Anchor

Figure 2.2.1.2: ROC curve for NFBSI-16 DRS-P, Improved versus Stable, according to EQ-5D-5L Usual Activities from Baseline to Cycle 5

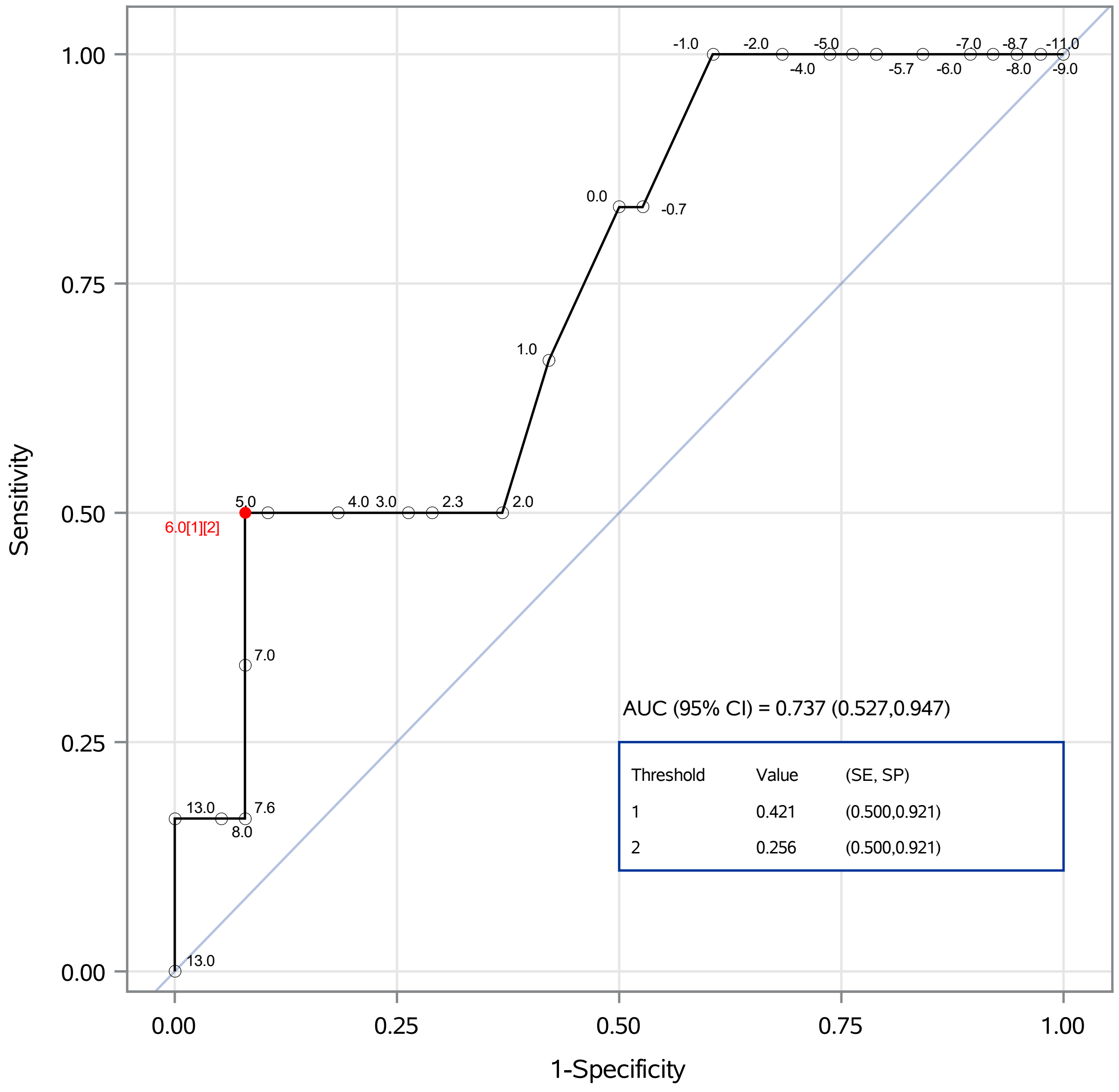

Threshold	Value	(SE, SP)
1	0.421	(0.500,0.921)
2	0.256	(0.500,0.921)

NFBSI: National Comprehensive Cancer Network Functional Assessment Of Cancer Therapy-Breast Cancer Symptom Index; DRSP: Disease-Related Symptoms Physical; IMPSTAB: 'Minimal/Moderate/Major Improvement' versus 'Stable' ; EQ-5D-5L Usual Activities Anchor

Figure 2.2.2.1: ROC curve for NFBSI-16 DRS-P, Improved versus Stable, according to EQ-5D-5L Pain from Baseline to Cycle 7

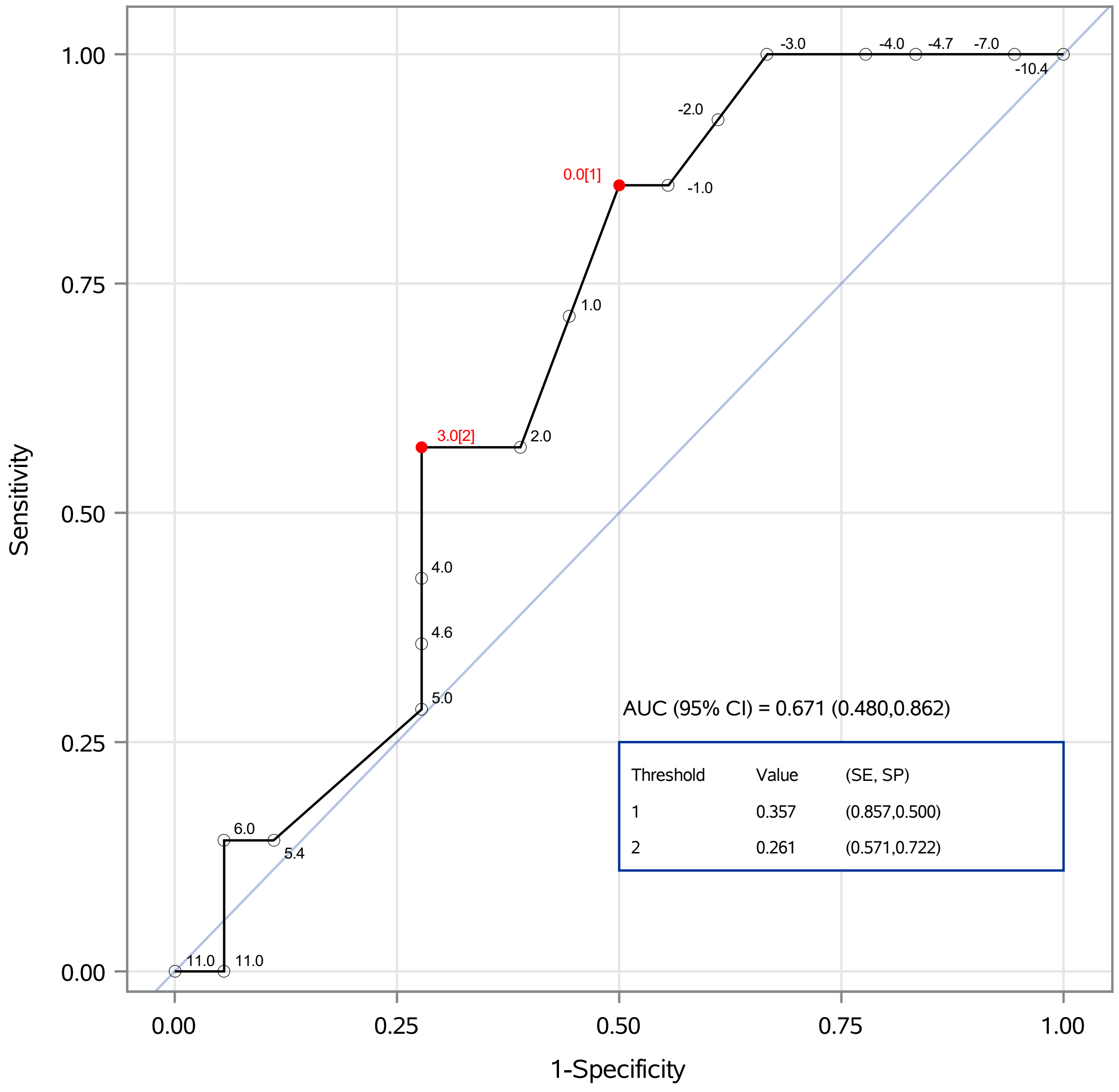

NFBSI: National Comprehensive Cancer Network Functional Assessment Of Cancer Therapy-Breast Cancer Symptom Index; DRSP: Disease-Related Symptoms Physical; IMPSTAB: 'Minimal/Moderate/Major Improvement' versus 'Stable' ; EQ-5D-5L Pain Anchor

Figure 2.2.2.2: ROC curve for NFBSI-16 DRS-P, Improved versus Stable, according to EQ-5D-5L Usual Activities from Baseline to Cycle 7

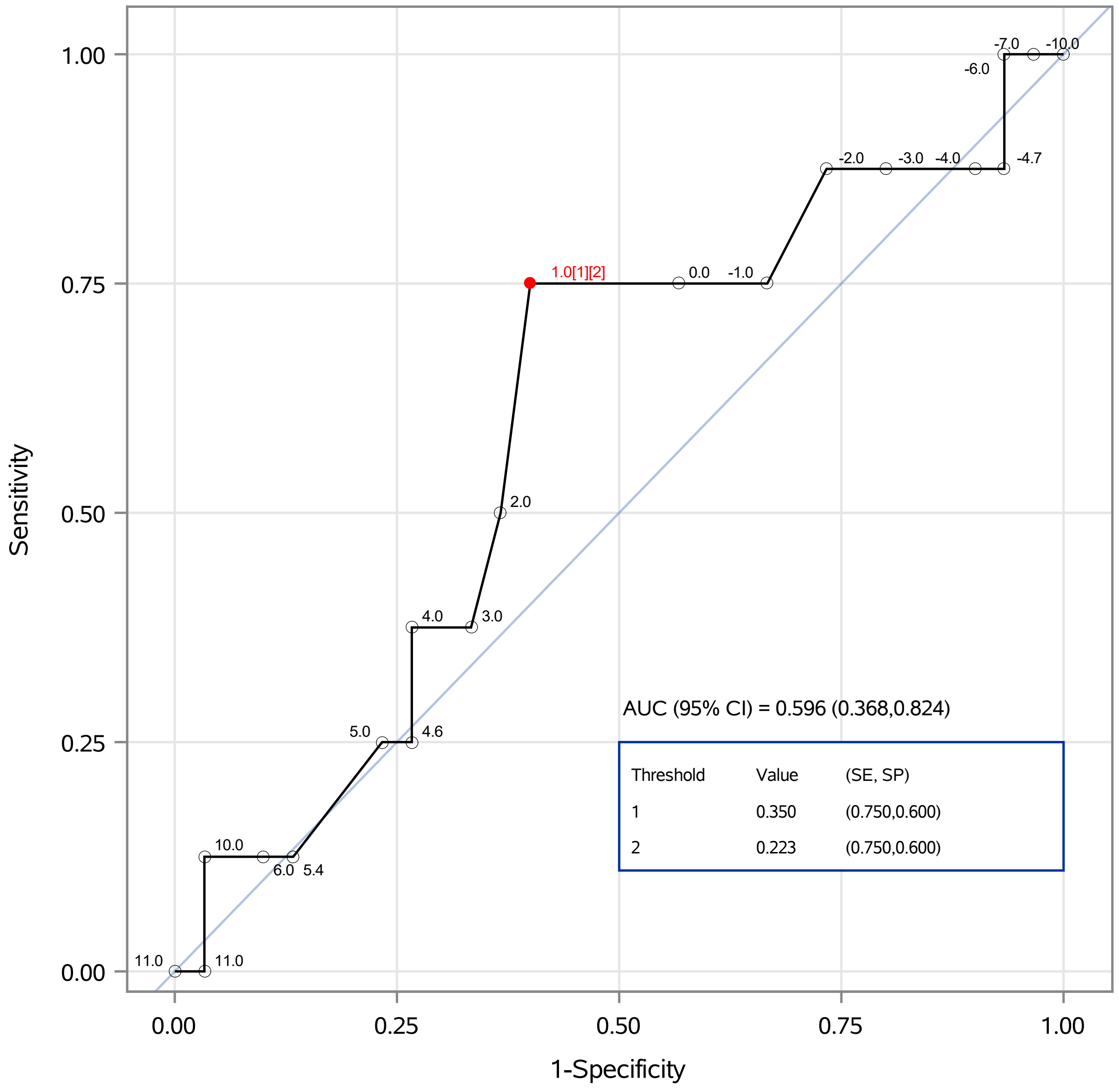

NFBSI: National Comprehensive Cancer Network Functional Assessment Of Cancer Therapy-Breast Cancer Symptom Index; DRSP: Disease-Related Symptoms Physical; IMPSTAB: 'Minimal/Moderate/Major Improvement' versus 'Stable' ; EQ-5D-5L Usual Activities Anchor

Figure 2.2.3.1: ROC curve for NFBSI-16 DRS-P, Improved versus Stable, according to EQ-5D-5L Pain from Baseline to Cycle 9

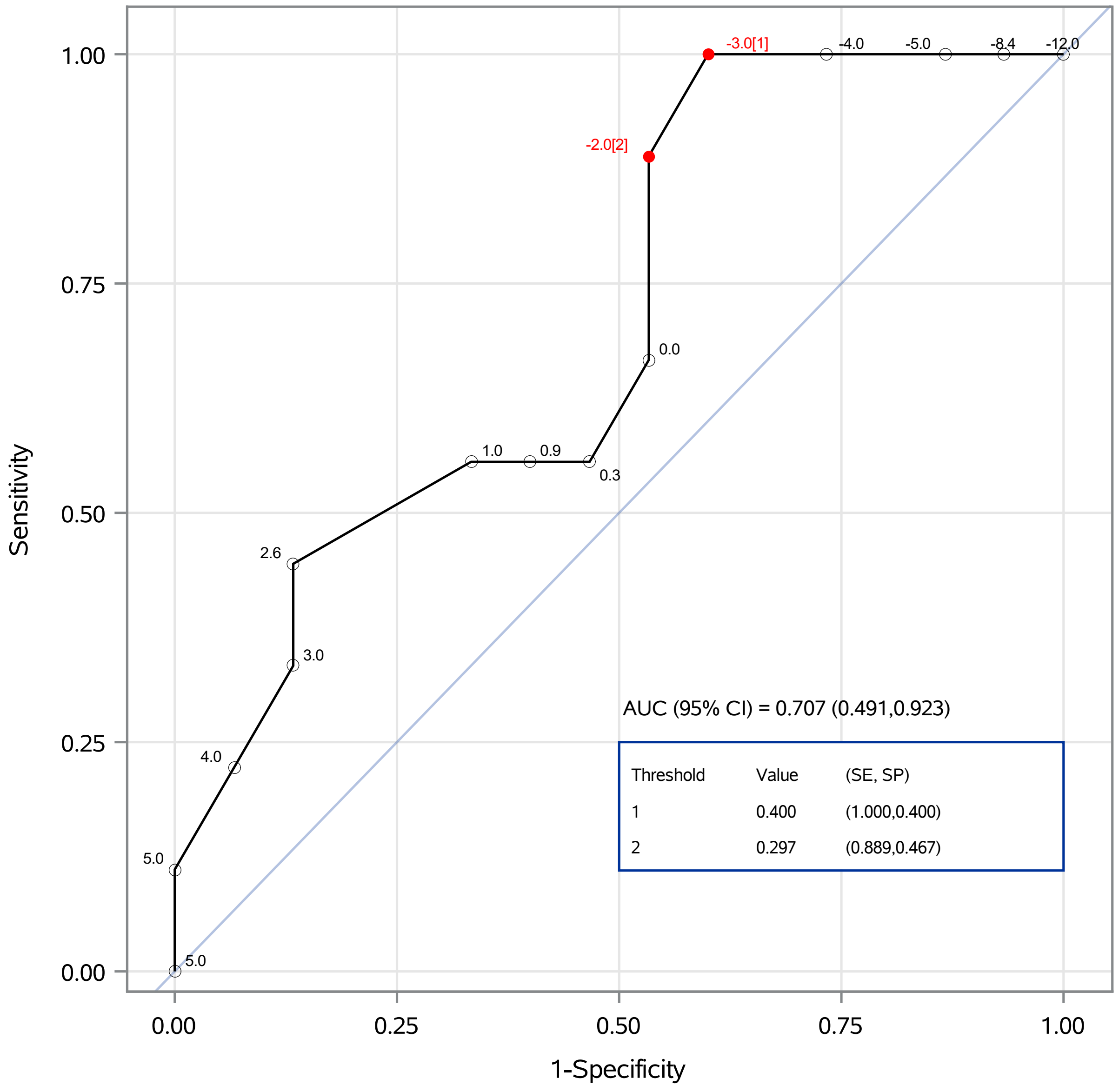

NFBSI: National Comprehensive Cancer Network Functional Assessment Of Cancer Therapy-Breast Cancer Symptom Index; DRSP: Disease-Related Symptoms Physical; IMPSTAB: 'Minimal/Moderate/Major Improvement' versus 'Stable' ; EQ-5D-5L Pain Anchor

Figure 2.2.3.2: ROC curve for NFBSI-16 DRS-P, Improved versus Stable, according to EQ-5D-5L Usual Activities from Baseline to Cycle 9

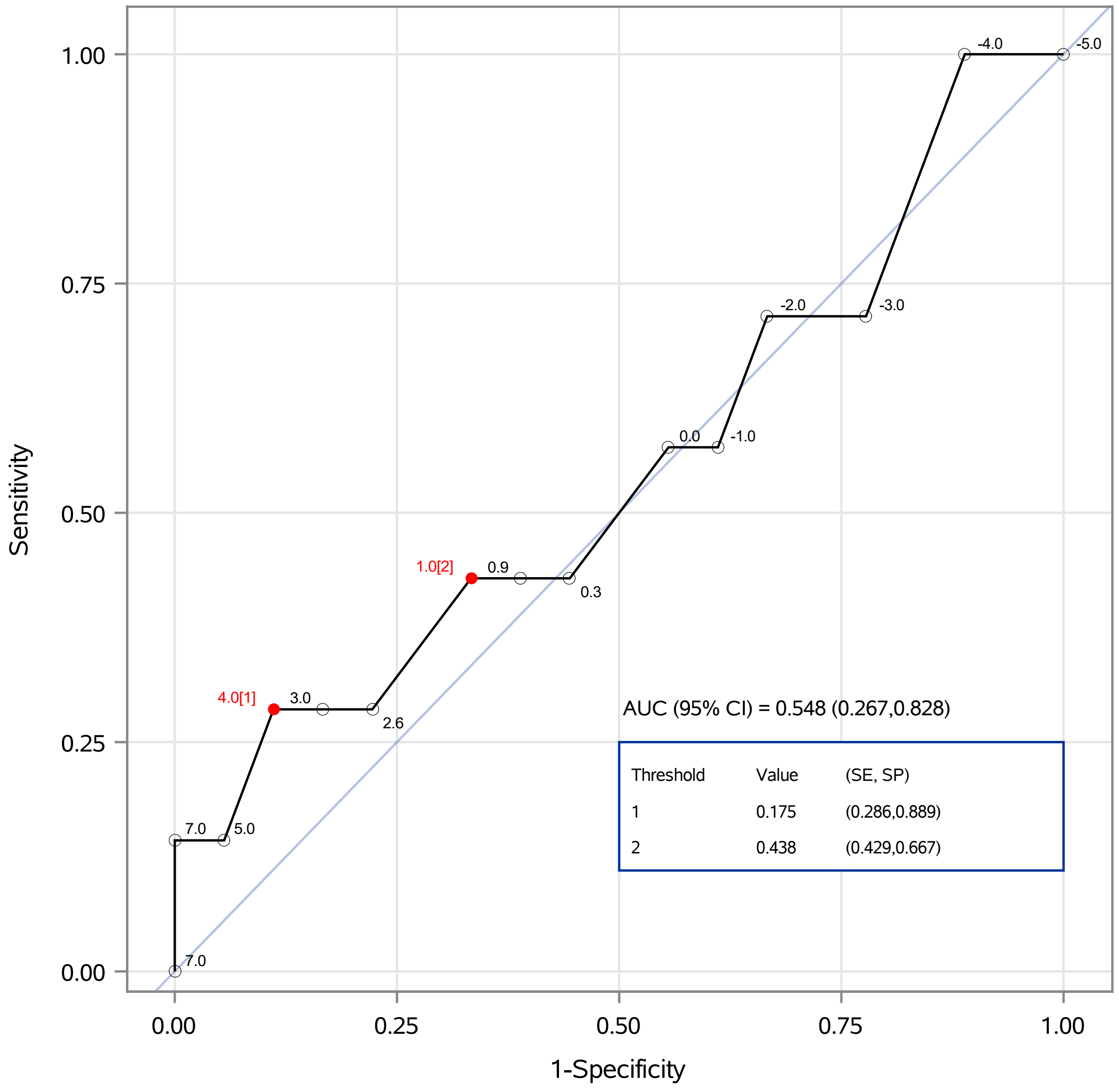

Threshold	Value	(SE, SP)
1	0.175	(0.286,0.889)
2	0.438	(0.429,0.667)

NFBSI: National Comprehensive Cancer Network Functional Assessment Of Cancer Therapy-Breast Cancer Symptom Index; DRSP: Disease-Related Symptoms Physical; IMPSTAB: 'Minimal/Moderate/Major Improvement' versus 'Stable' ; EQ-5D-5L Usual Activities Anchor

Figure 2.3.1.2: ROC curve for NFBSI-16 DRS-E, Improved versus Stable, according to EQ-5D-5L VAS from Baseline to Cycle 5

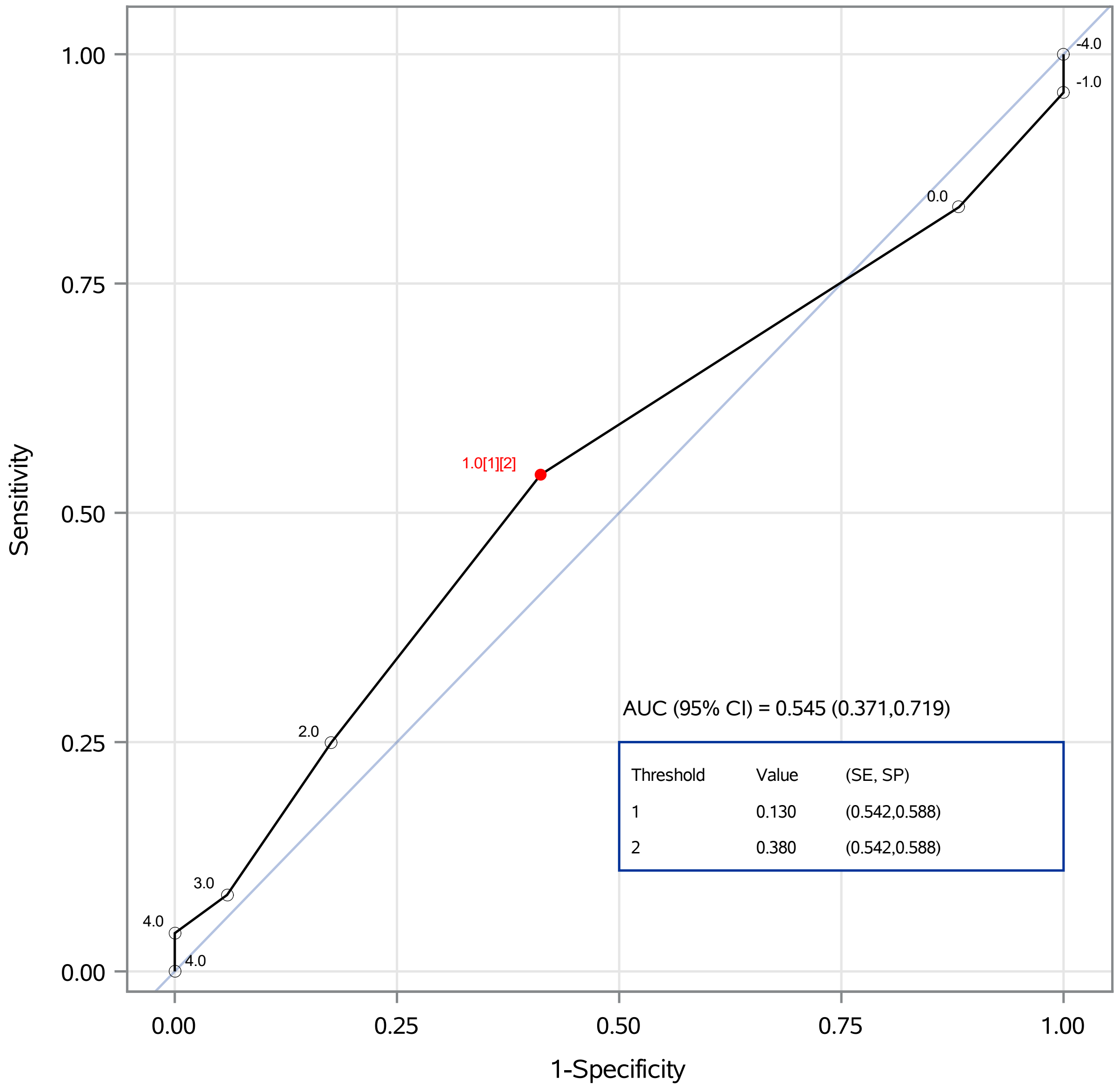

NFBSI: National Comprehensive Cancer Network Functional Assessment Of Cancer Therapy-Breast Cancer Symptom Index; DRSE: Disease-Related Symptoms Emotional; IMPSTAB: 'Minimal/Moderate/Major Improvement' versus 'Stable' ; EQ-5D-5L VAS Anchor

Figure 2.3.2.2: ROC curve for NFBSI-16 DRS-E, Improved versus Stable, according to EQ-5D-5L VAS from Baseline to Cycle 7

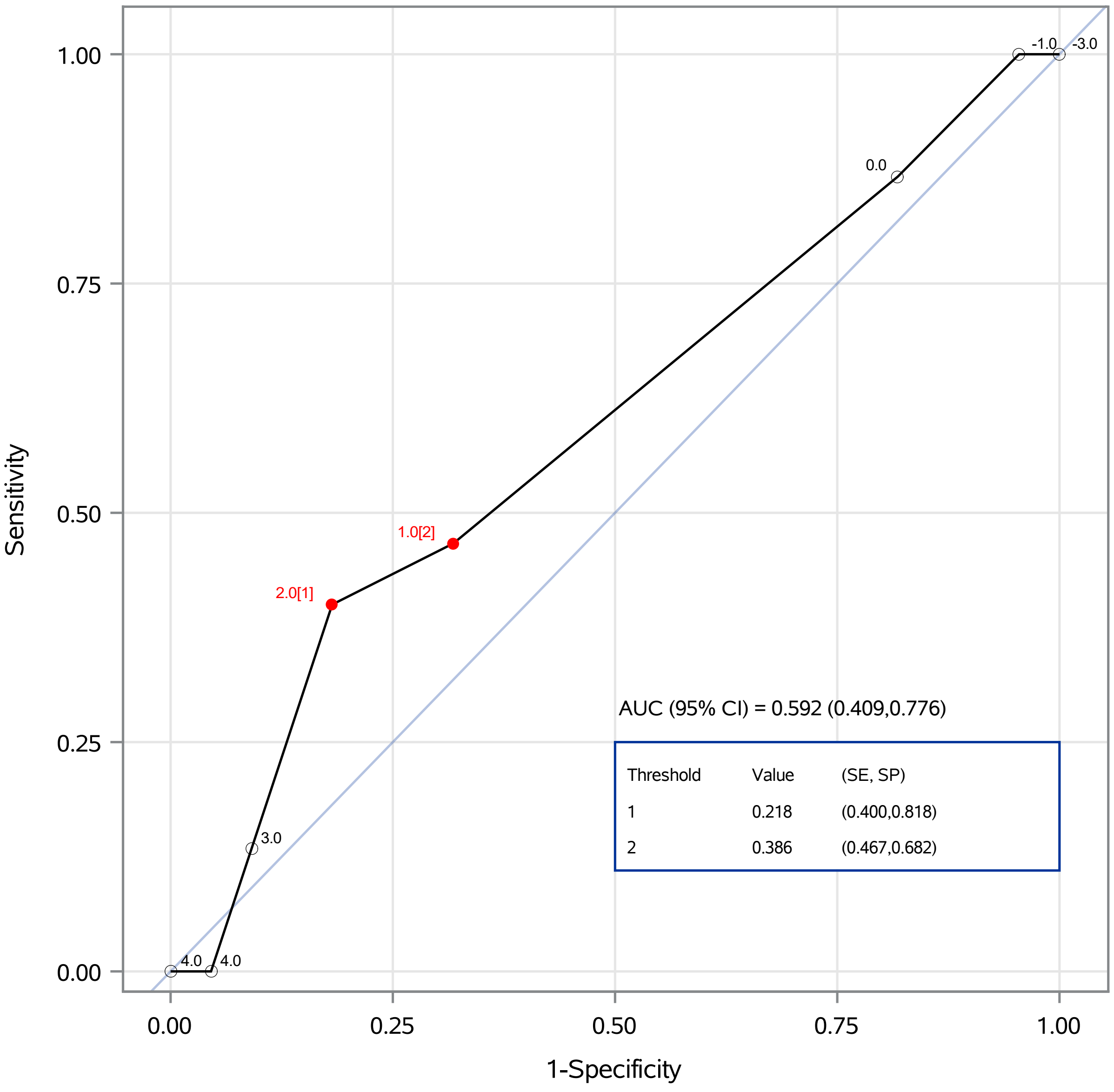

Threshold	Value	(SE, SP)
1	0.218	(0.400,0.818)
2	0.386	(0.467,0.682)

NFBSI: National Comprehensive Cancer Network Functional Assessment Of Cancer Therapy-Breast Cancer Symptom Index; DRSE: Disease-Related Symptoms Emotional; IMPSTAB: 'Minimal/Moderate/Major Improvement' versus 'Stable' ; EQ-5D-5L VAS Anchor

Figure 2.3.3.2: ROC curve for NFBSI-16 DRS-E, Improved versus Stable, according to EQ-5D-5L VAS from Baseline to Cycle 9

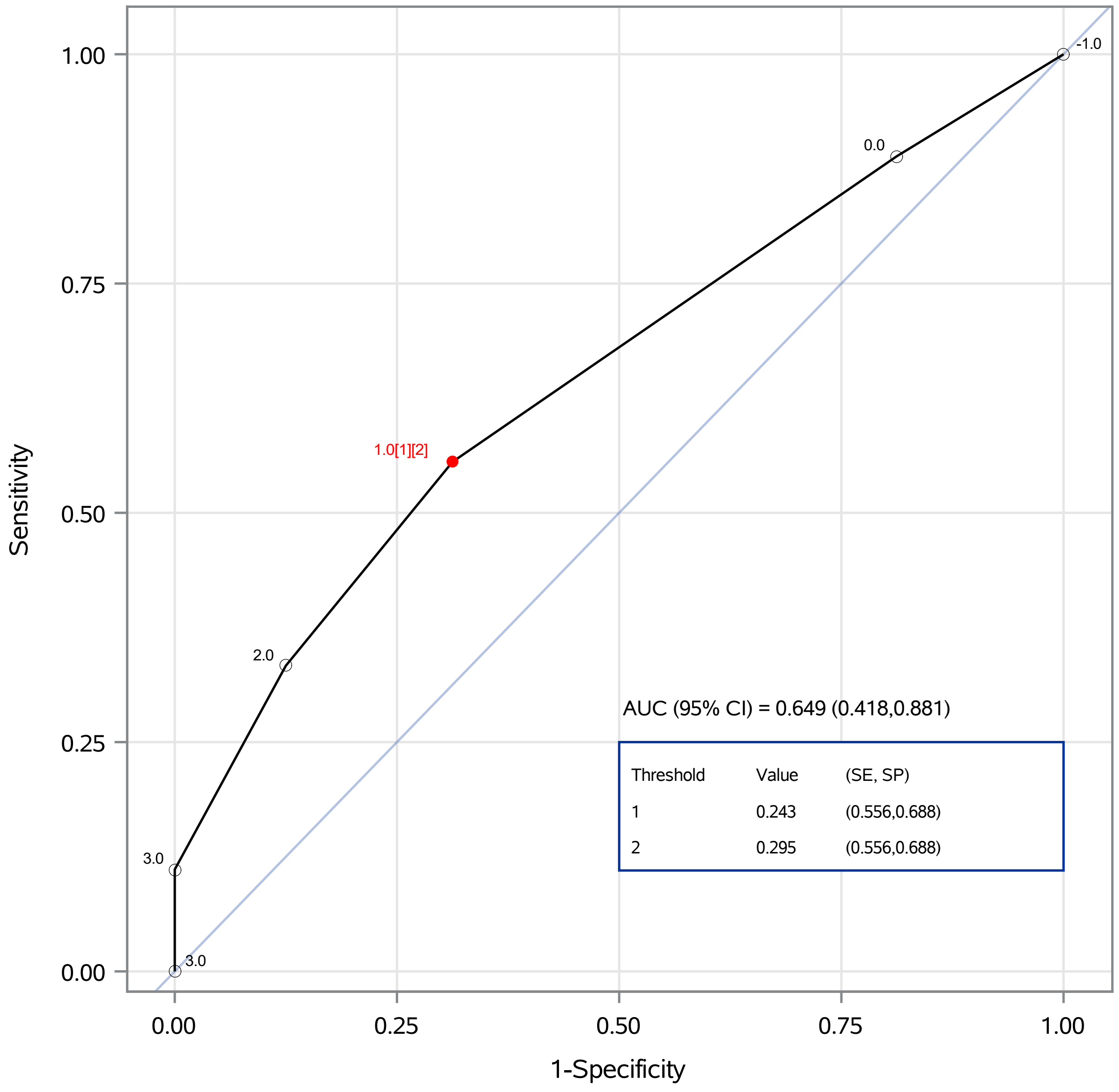

Threshold	Value	(SE, SP)
1	0.243	(0.556,0.688)
2	0.295	(0.556,0.688)

NFBSI: National Comprehensive Cancer Network Functional Assessment Of Cancer Therapy-Breast Cancer Symptom Index; DRSE: Disease-Related Symptoms Emotional; IMPSTAB: 'Minimal/Moderate/Major Improvement' versus 'Stable' ; EQ-5D-5L VAS Anchor

Figure 3.1.1.1: ROC curve for NFBSI-16 Total score, Worsened versus Stable, according to EQ-5D-5L VAS from Baseline to Cycle 5

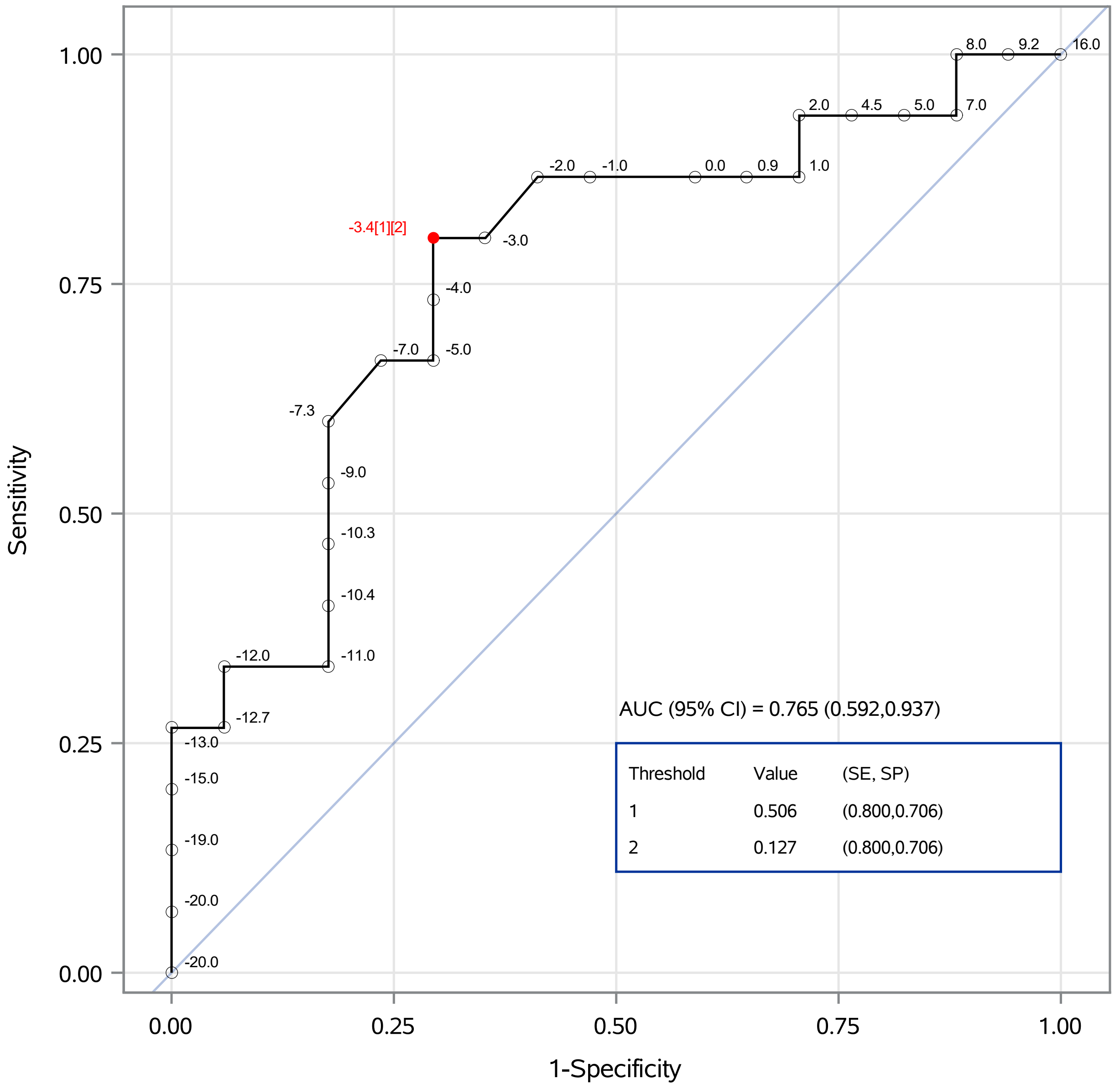

Threshold	Value	(SE, SP)
1	0.506	(0.800,0.706)
2	0.127	(0.800,0.706)

NFBSI: National Comprehensive Cancer Network Functional Assessment Of Cancer Therapy-Breast Cancer Symptom Index; WORSTAB: 'Minimal/Moderate/Major Worsening' versus 'Stable'; EQ-5D-5L VAS Anchor

Figure 3.1.2.1: ROC curve for NFBSI-16 Total score, Worsened versus Stable, according to EQ-5D-5L VAS from Baseline to Cycle 7

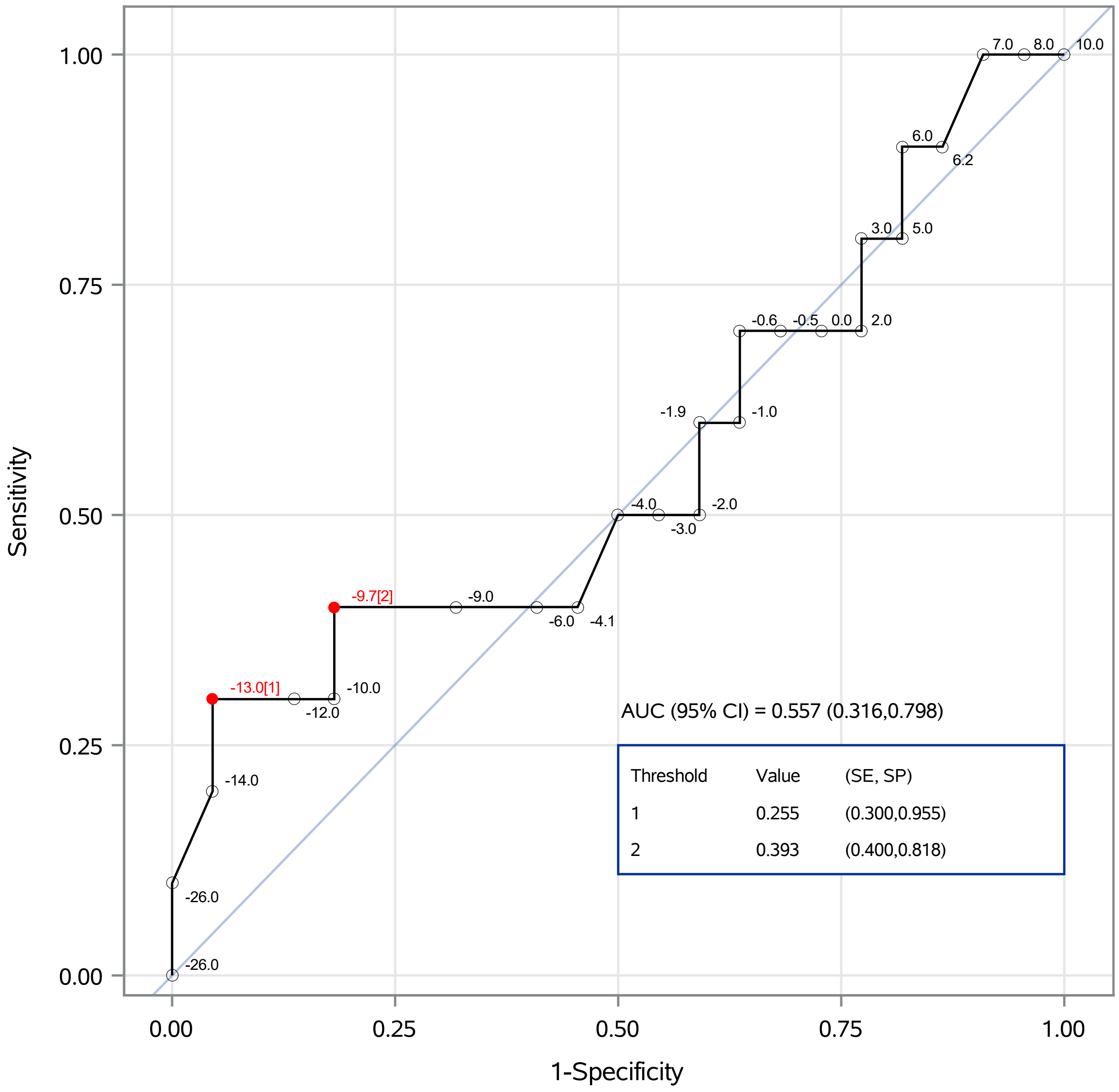

Threshold	Value	(SE, SP)
1	0.255	(0.300,0.955)
2	0.393	(0.400,0.818)

NFBSI: National Comprehensive Cancer Network Functional Assessment Of Cancer Therapy-Breast Cancer Symptom Index; WORSTAB: 'Minimal/Moderate/Major Worsening' versus 'Stable'; EQ-5D-5L VAS Anchor

Figure 3.1.3.1: ROC curve for NFBSI-16 Total score, Worsened versus Stable, according to EQ-5D-5L VAS from Baseline to Cycle 9

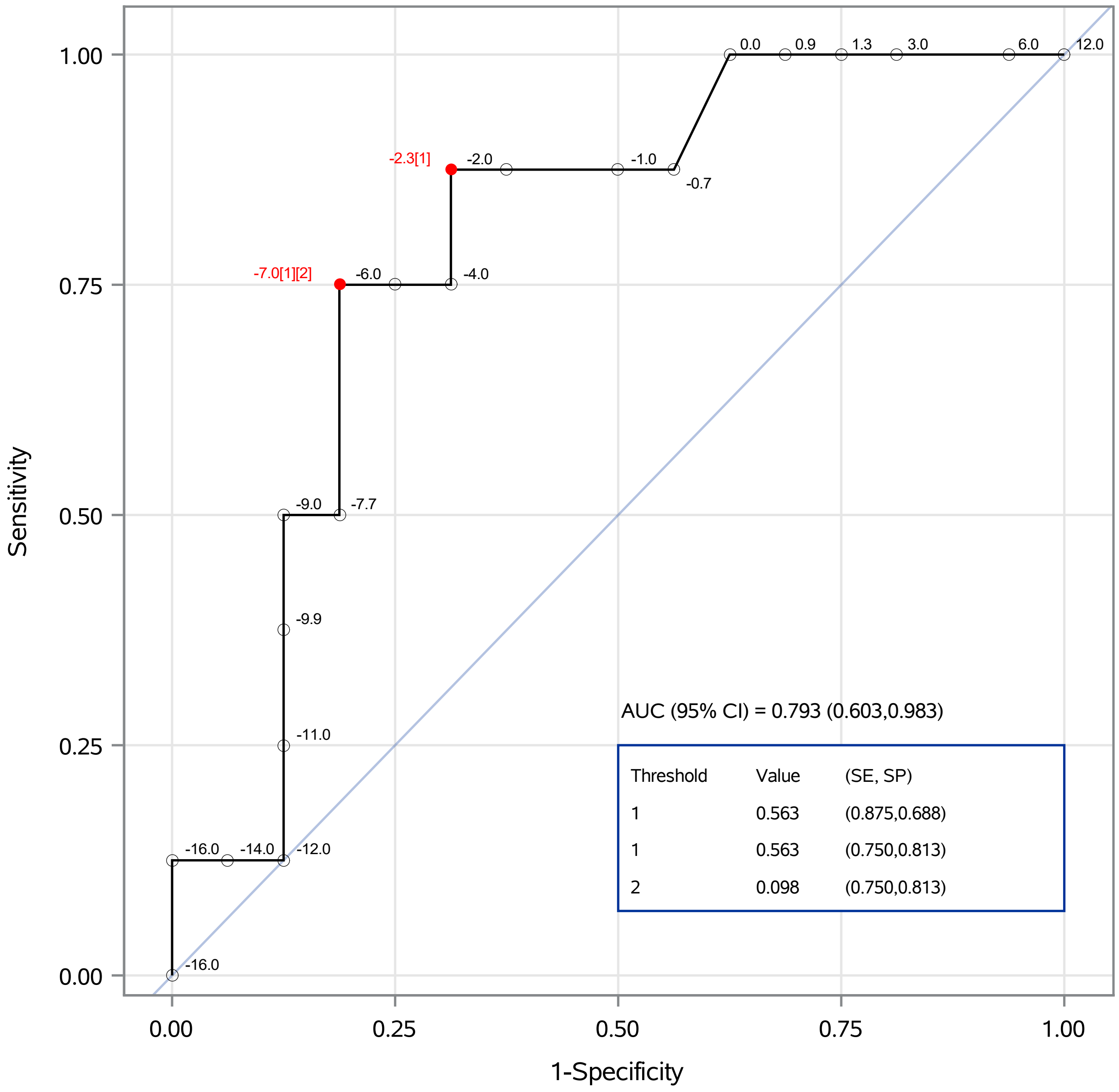

NFBSI: National Comprehensive Cancer Network Functional Assessment Of Cancer Therapy-Breast Cancer Symptom Index; WORSTAB: 'Minimal/Moderate/Major Worsening' versus 'Stable'; EQ-5D-5L VAS Anchor

Figure 3.2.1.1: ROC curve for NFBSI-16 DRS-P, Worsened versus Stable, according to EQ-5D-5L Pain from Baseline to Cycle 5

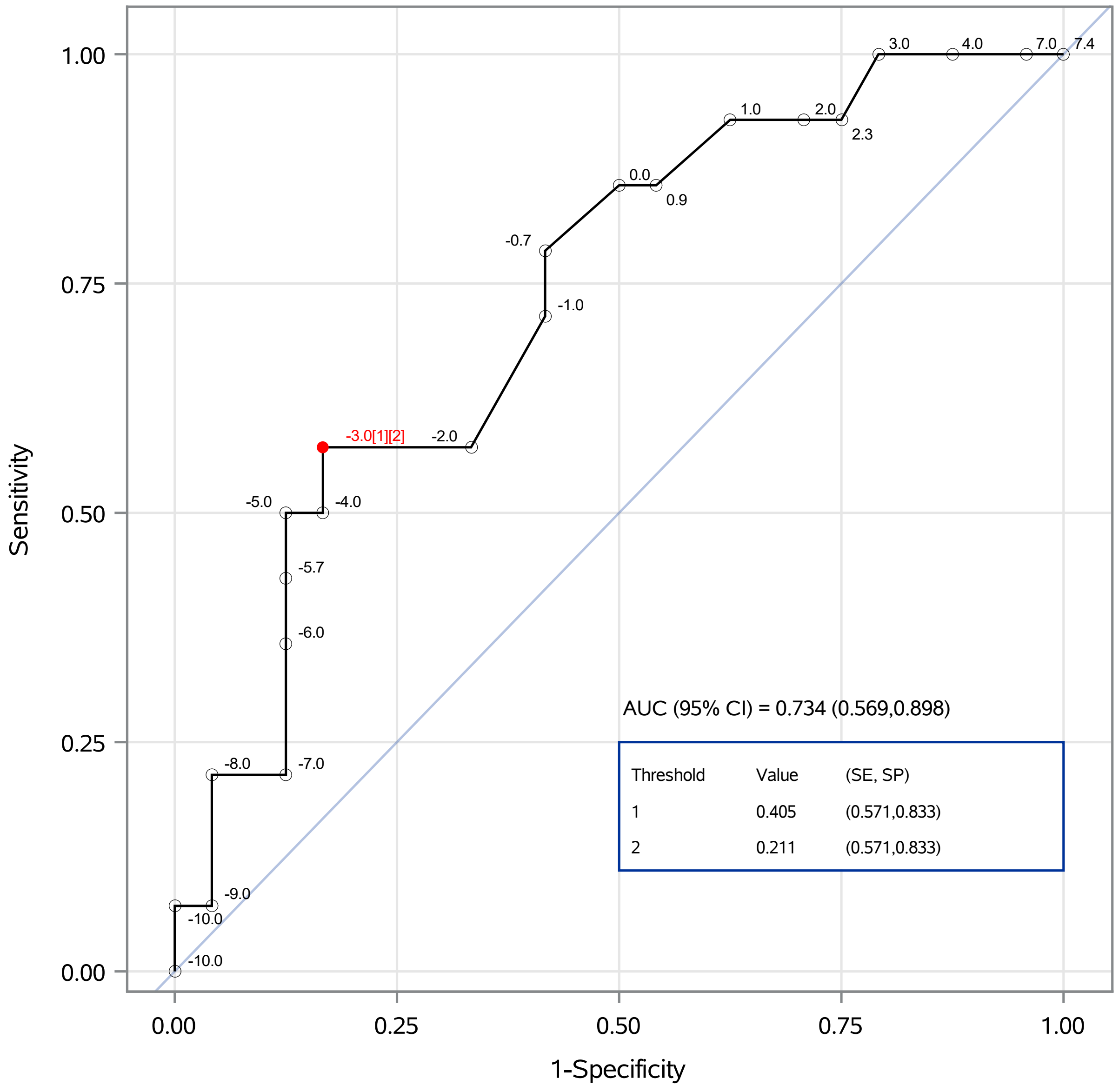

NFBSI: National Comprehensive Cancer Network Functional Assessment Of Cancer Therapy-Breast Cancer Symptom Index; DRSP: Disease-Related Symptoms Physical; WORSTAB: 'Minimal/Moderate/Major Worsening' versus 'Stable'; EQ-5D-5L Pain Anchor

Figure 3.2.1.2: ROC curve for NFBSI-16 DRS-P, Worsened versus Stable, according to EQ-5D-5L Usual Activities from Baseline to Cycle 5

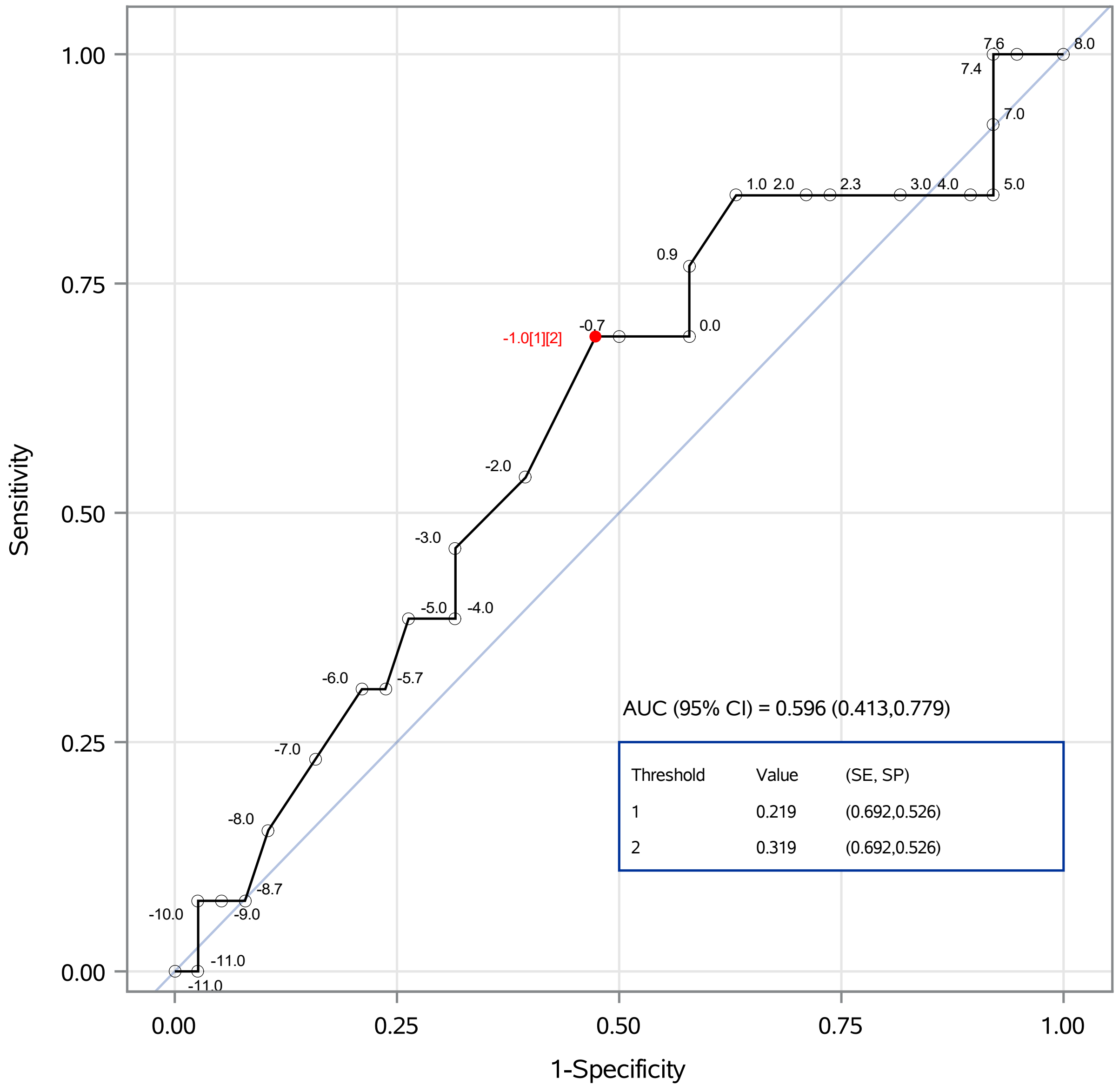

NFBSI: National Comprehensive Cancer Network Functional Assessment Of Cancer Therapy-Breast Cancer Symptom Index; DRSP: Disease-Related Symptoms Physical; WORSTAB: 'Minimal/Moderate/Major Worsening' versus 'Stable'; EQ-5D-5L Usual Activities Anchor

Figure 3.2.2.1: ROC curve for NFBSI-16 DRS-P, Woresened versus Stable, according to EQ-5D-5L Pain from Baseline to Cycle 7

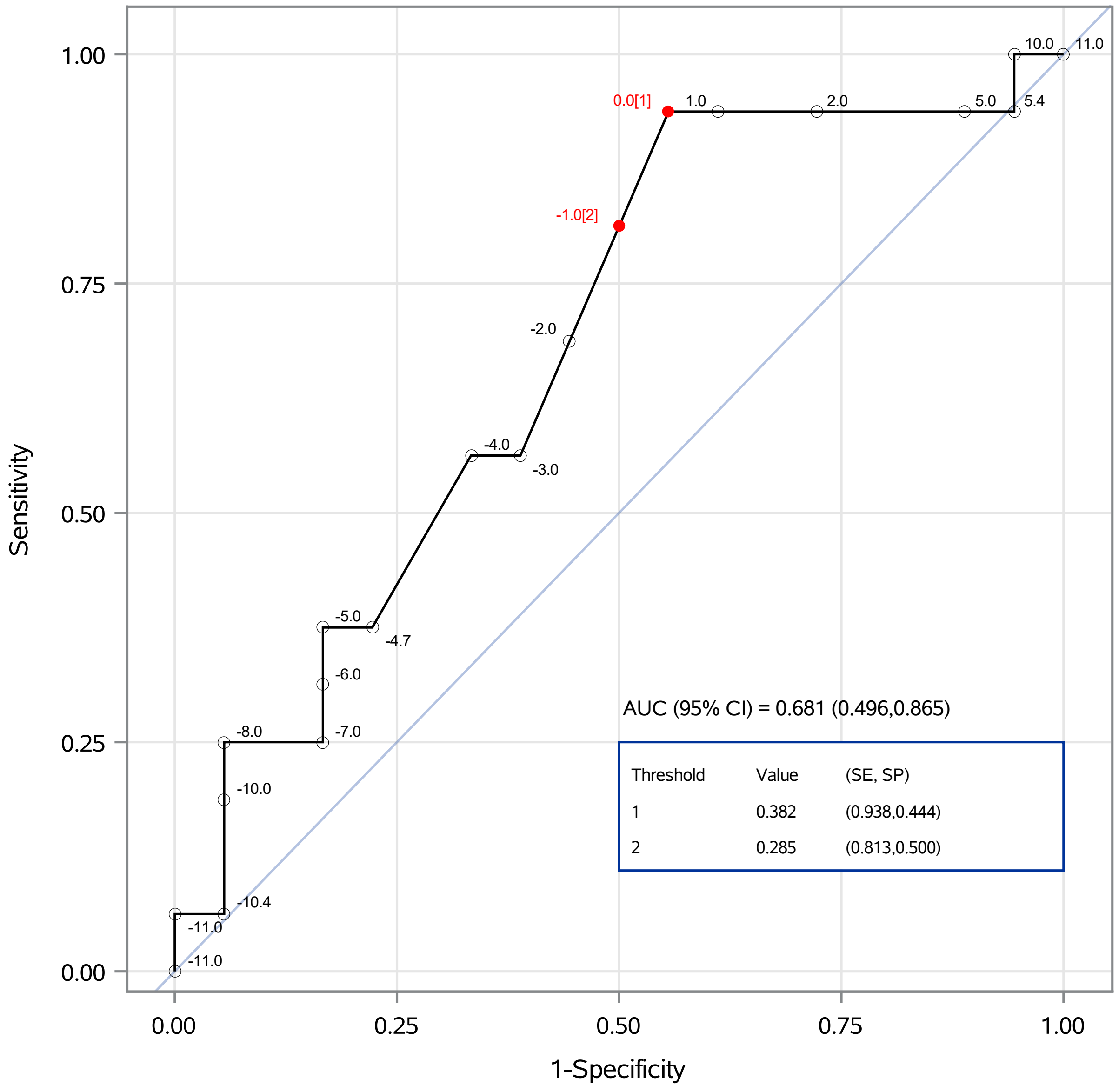

NFBSI: National Comprehensive Cancer Network Functional Assessment Of Cancer Therapy-Breast Cancer Symptom Index; DRSP: Disease-Related Symptoms Physical; WORSTAB: 'Minimal/Moderate/Major Worsening' versus 'Stable'; EQ-5D-5L Pain Anchor

Figure 3.2.2.2: ROC curve for NFBSI-16 DRS-P, Worsened versus Stable, according to EQ-5D-5L Usual Activities from Baseline to Cycle 7

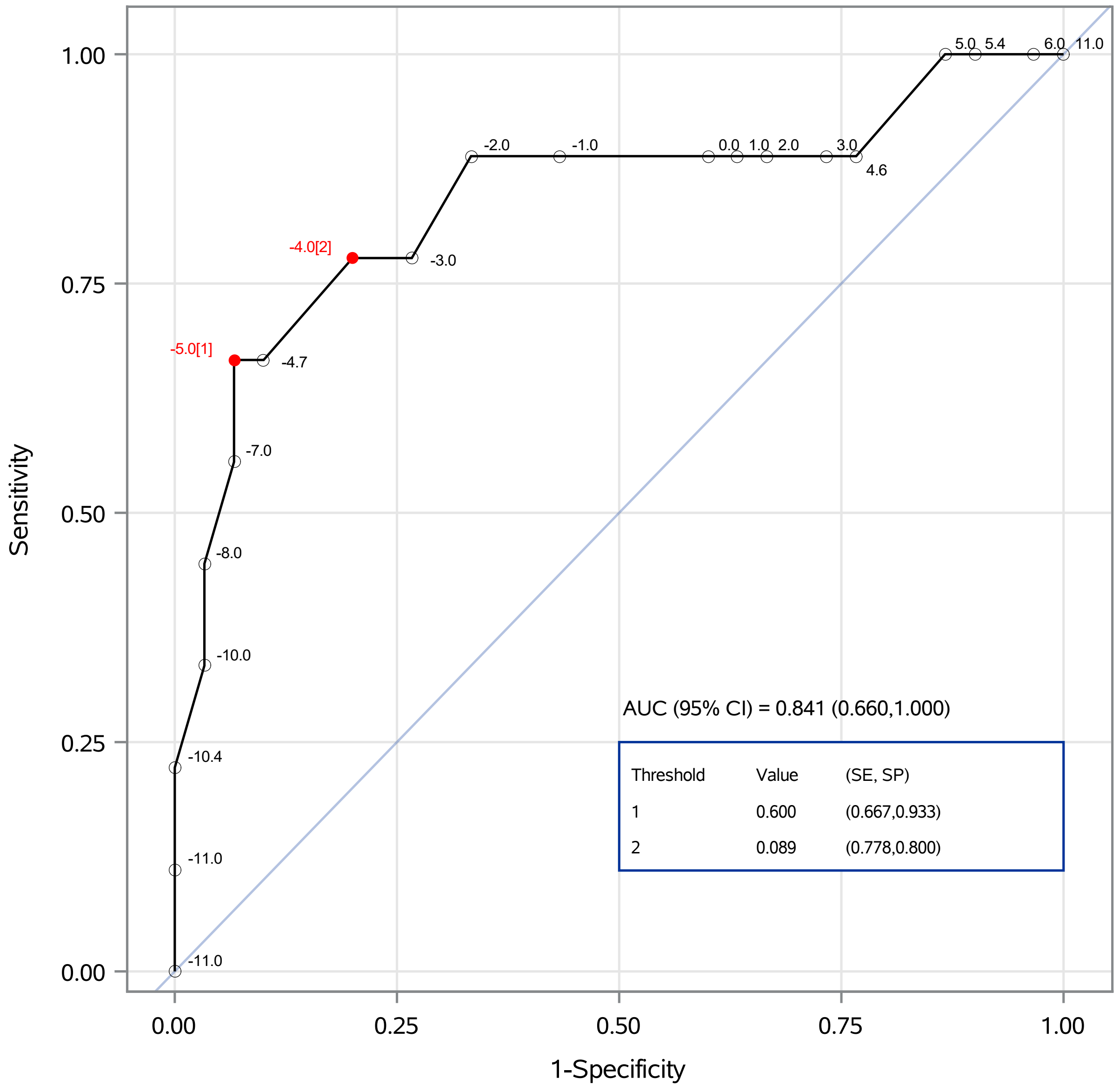

NFBSI: National Comprehensive Cancer Network Functional Assessment Of Cancer Therapy-Breast Cancer Symptom Index; DRSP: Disease-Related Symptoms Physical; WORSTAB: 'Minimal/Moderate/Major Worsening' versus 'Stable'; EQ-5D-5L Usual Activities Anchor

Figure 3.2.3.1: ROC curve for NFBSI-16 DRS-P, Woresened versus Stable, according to EQ-5D-5L Pain from Baseline to Cycle 9

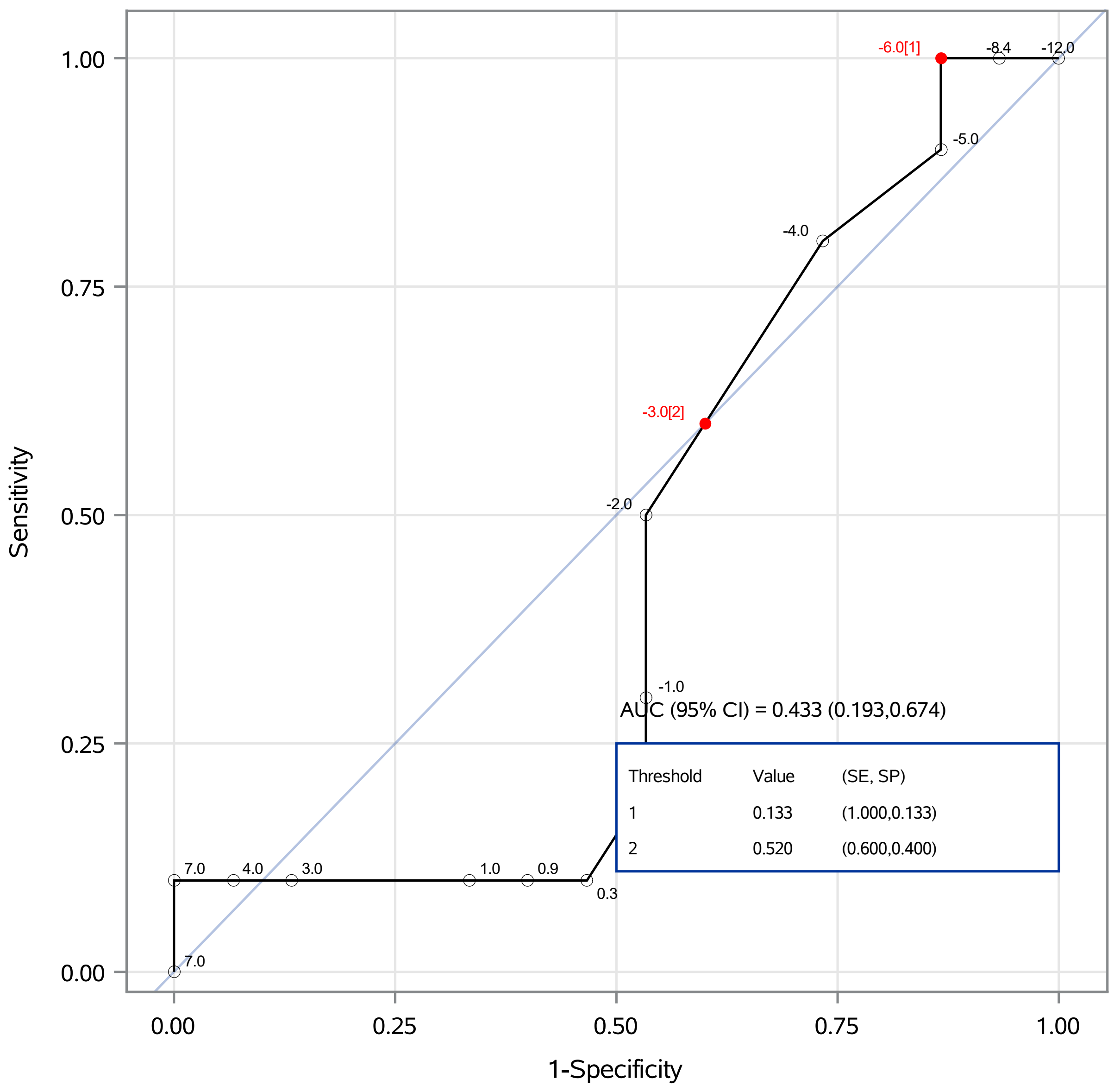

NFBSI: National Comprehensive Cancer Network Functional Assessment Of Cancer Therapy-Breast Cancer Symptom Index; DRSP: Disease-Related Symptoms Physical; WORSTAB: 'Minimal/Moderate/Major Worsening' versus 'Stable'; EQ-5D-5L Pain Anchor

Figure 3.2.3.2: ROC curve for NFBSI-16 DRS-P, Worsened versus Stable, according to EQ-5D-5L Usual Activities from Baseline to Cycle 9

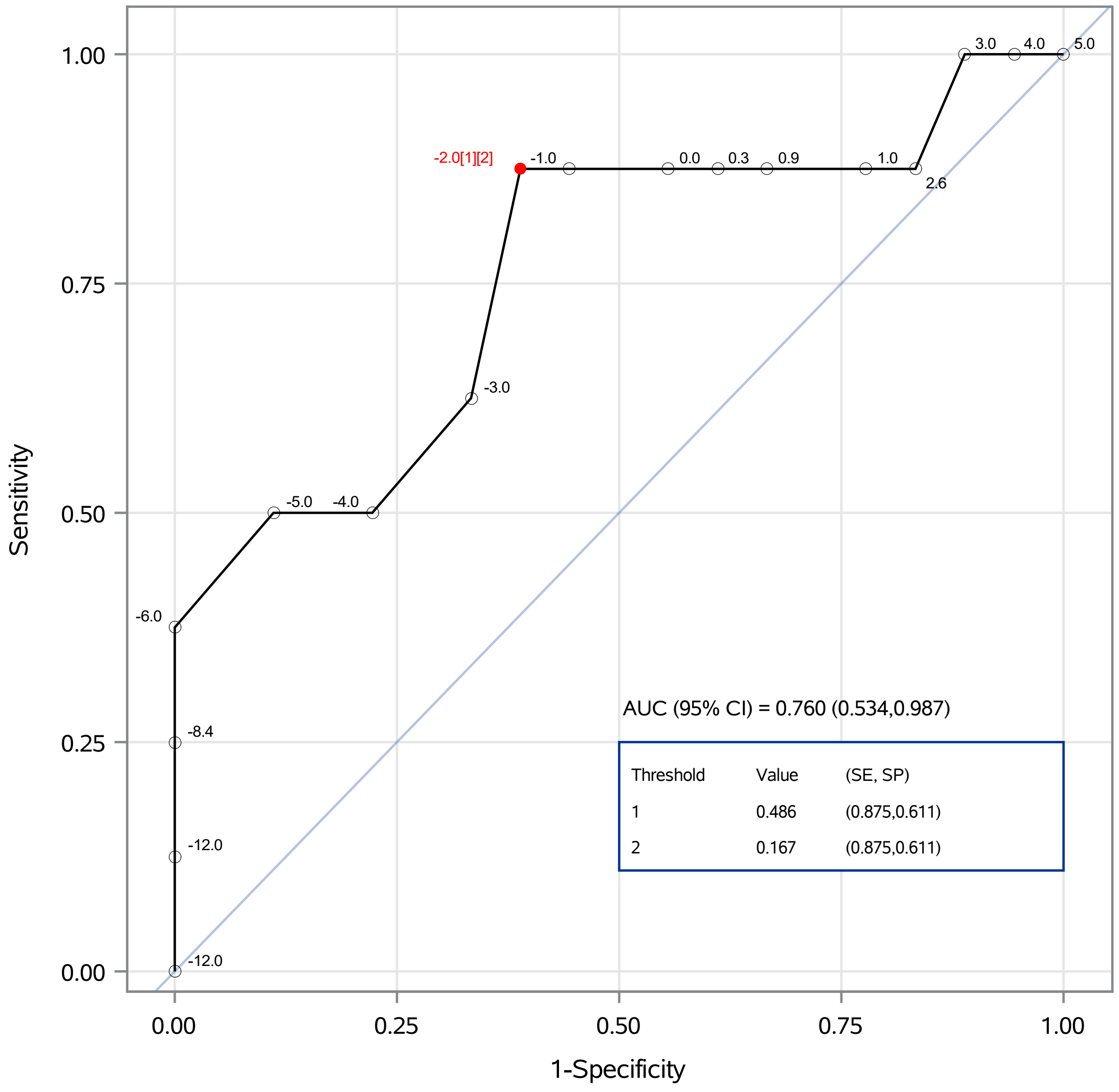

NFBSI: National Comprehensive Cancer Network Functional Assessment Of Cancer Therapy-Breast Cancer Symptom Index; DRSP: Disease-Related Symptoms Physical; WORSTAB: 'Minimal/Moderate/Major Worsening' versus 'Stable'; EQ-5D-5L Usual Activities Anchor

Figure 3.3.1.2: ROC curve for NFBSI-16 DRS-E, Worsened versus Stable, according to EQ-5D-5L VAS from Baseline to Cycle 5

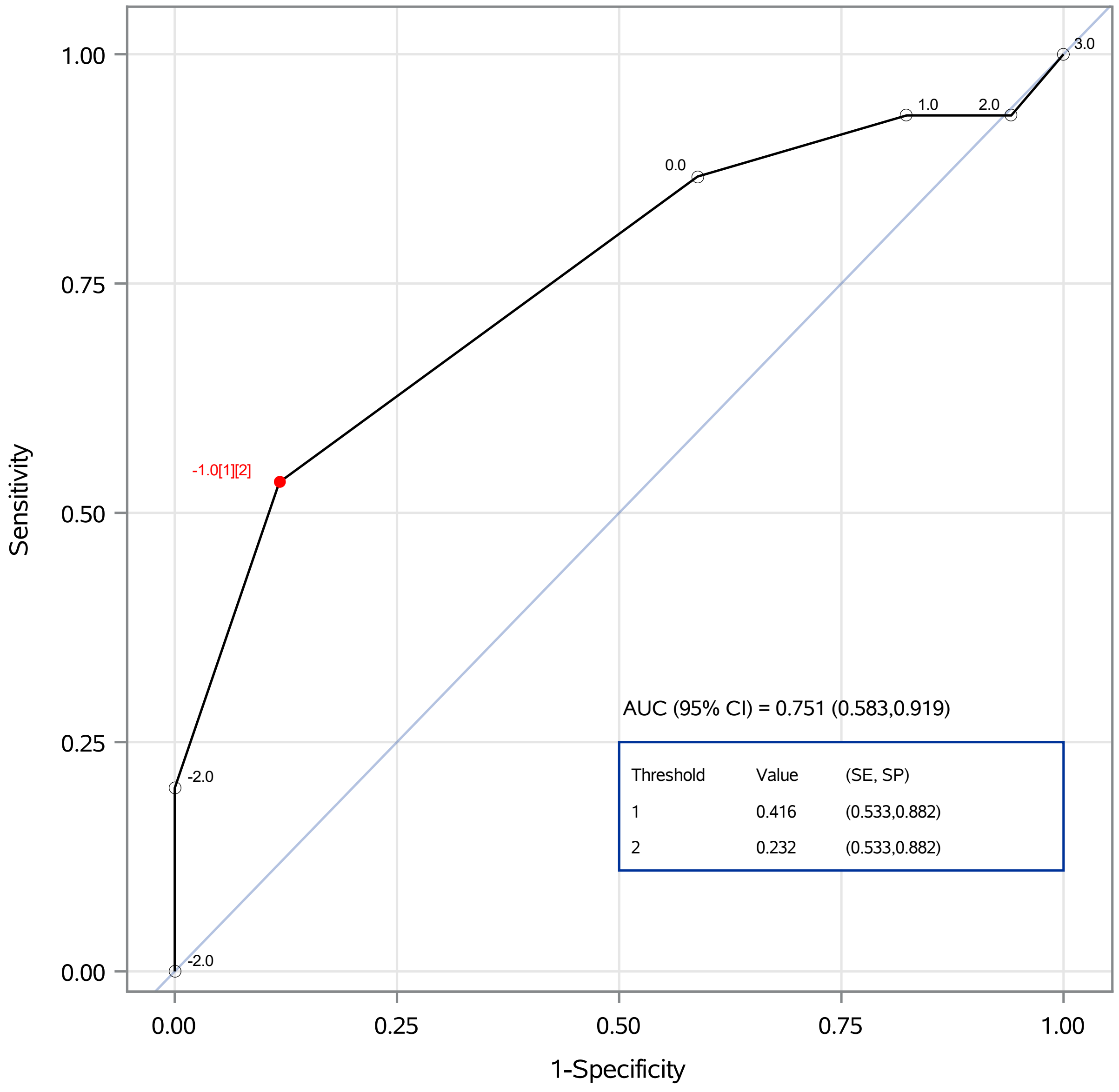

NFBSI: National Comprehensive Cancer Network Functional Assessment Of Cancer Therapy-Breast Cancer Symptom Index; DRSE: Disease-Related Symptoms Emotional; WORSTAB: 'Minimal/Moderate/Major Worsening' versus 'Stable'; EQ-5D-5L VAS Anchor

Figure 3.3.2.2: ROC curve for NFBSI-16 DRS-E, Worsened versus Stable, according to EQ-5D-5L VAS from Baseline to Cycle 7

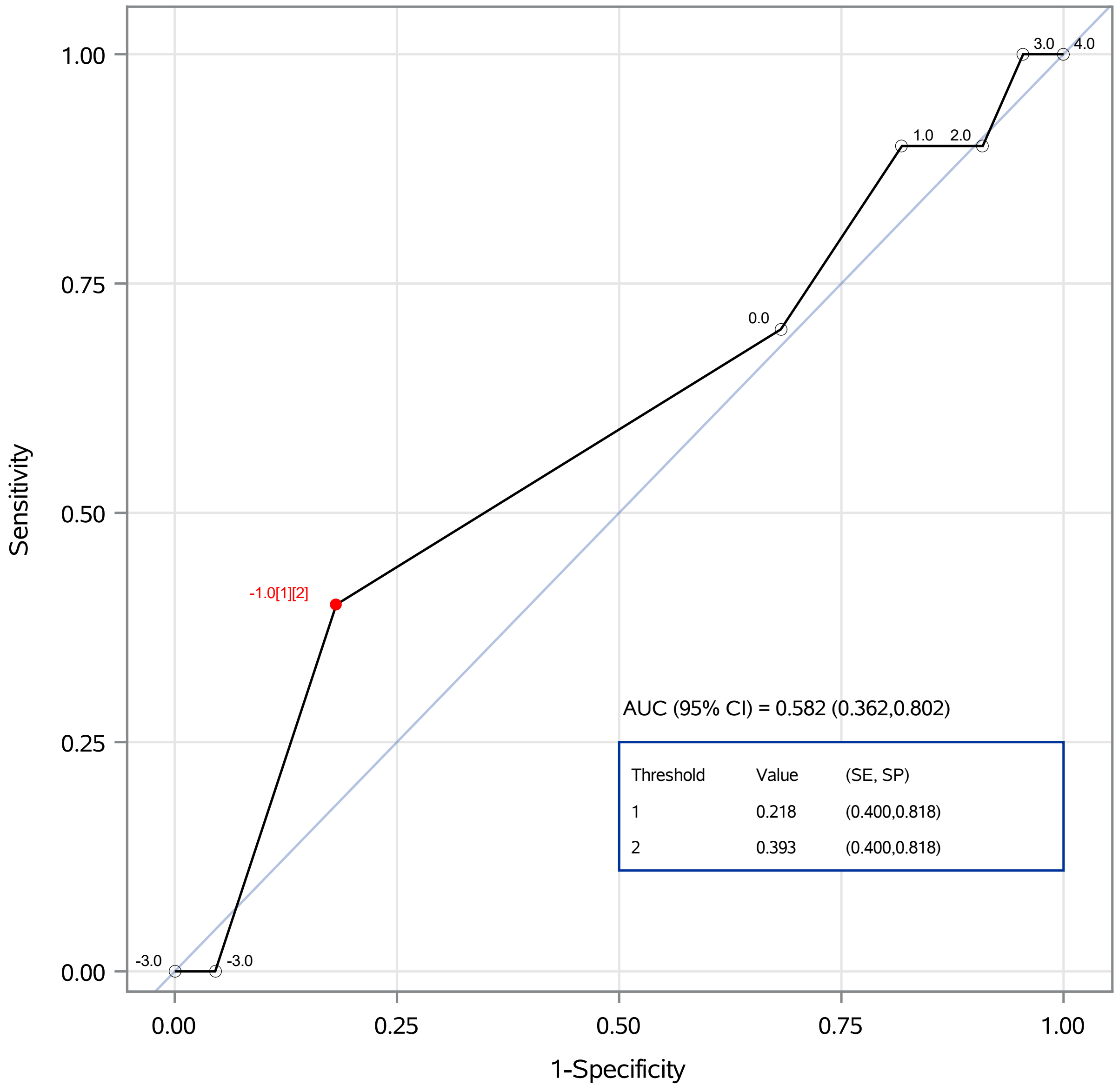

NFBSI: National Comprehensive Cancer Network Functional Assessment Of Cancer Therapy-Breast Cancer Symptom Index; DRSE: Disease-Related Symptoms Emotional; WORSTAB: 'Minimal/Moderate/Major Worsening' versus 'Stable'; EQ-5D-5L VAS Anchor

Figure 3.3.3.2: ROC curve for NFBSI-16 DRS-E, Worsened versus Stable, according to EQ-5D-5L VAS from Baseline to Cycle 9

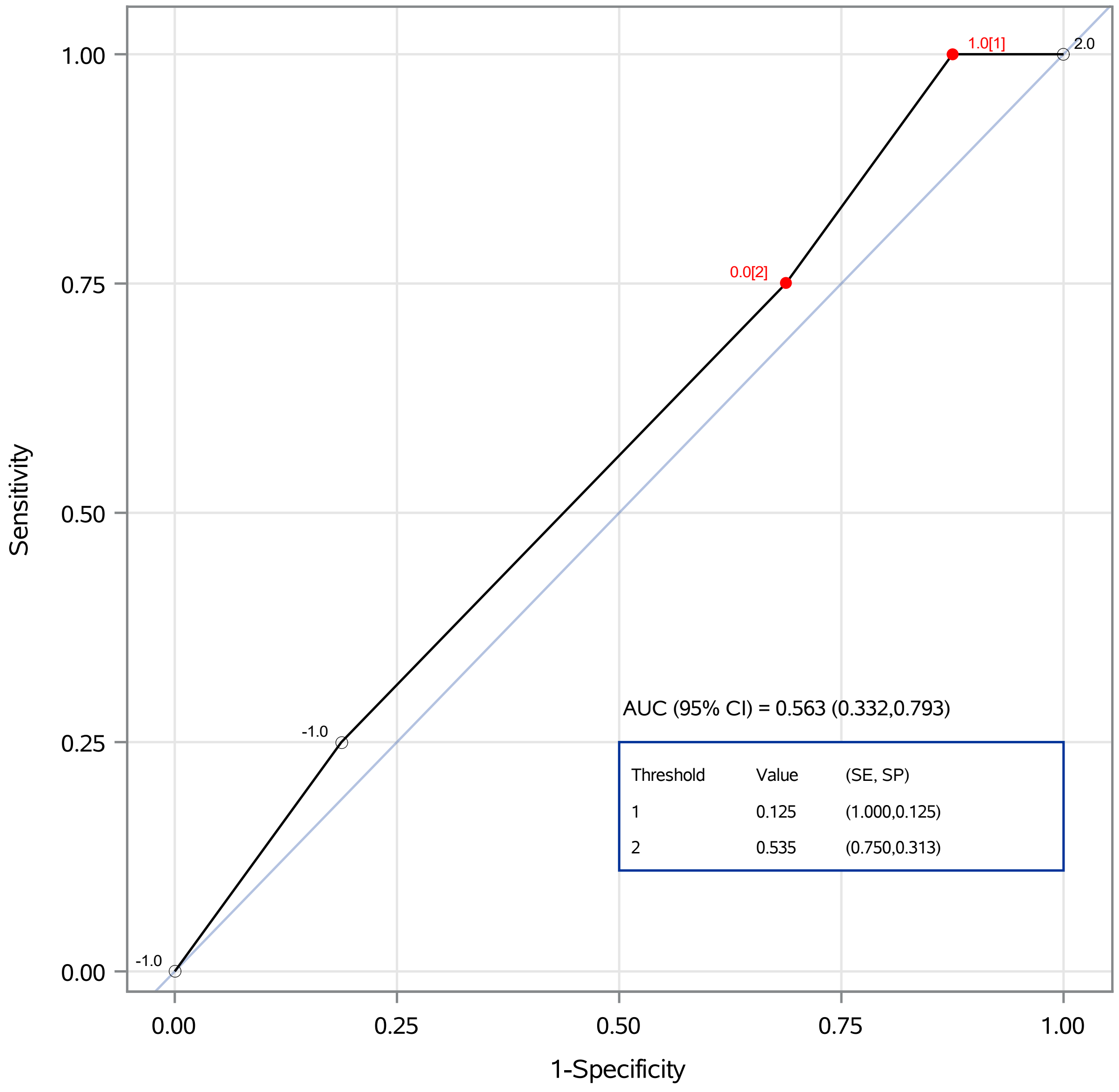

Threshold	Value	(SE, SP)
1	0.125	(1.000,0.125)
2	0.535	(0.750,0.313)

NFBSI: National Comprehensive Cancer Network Functional Assessment Of Cancer Therapy-Breast Cancer Symptom Index; DRSE: Disease-Related Symptoms Emotional; WORSTAB: 'Minimal/Moderate/Major Worsening' versus 'Stable'; EQ-5D-5L VAS Anchor
