# Supplementary material for: Validity, reliability, responsiveness, and clinically meaningful change threshold estimates of the National Comprehensive Cancer Network-Functional Assessment of Cancer Therapy-Breast Cancer Symptom Index (NFBSI-16)
Source: J Patient Rep Outcomes. 2024 Aug 15;8:97. doi: 10.1186/s41687-024-00776-y (PMC11327234; doi:10.1186/s41687-024-00776-y)
Supplement: Supplementary file 2 — Supplementary Material 2 [file 41687_2024_776_MOESM2_ESM.docx]

Supplementary Table 1: Descriptions of the anchor measures and improvement/worsening thresholds used for meaningful change analyses

| **Anchor #** | **Anchor Measure** | **Definition** | **Relevant scores** |
| --- | --- | --- | --- |
| **1** | EQ-5D-5L Pain | - Major improvement: ≤-3 point change - Moderate improvement: -2 point change - Minimal improvement: -1 point change - Stable: no change - Minimal worsening: 1 point change - Moderate worsening: 2 point change - Major worsening: ≥3 point change | - DRS-P |
| **2** | EQ-5D-5L Anxiety/ Depression | - Major improvement: ≤-3 point change - Moderate improvement: -2 point change - Minimal improvement: -1 point change - Stable: no change - Minimal worsening: 1 point change - Moderate worsening: 2 point change - Major worsening: ≥3 point change | - DRS-E |
| **3** | EQ-5D-5L Usual Activities | - Major improvement: ≤-3 point change - Moderate improvement: -2 point change - Minimal improvement: -1 point change - Stable: no change - Minimal worsening: 1 point change - Moderate worsening: 2 point change - Major worsening: ≥3 point change | - DRS-P |
| **4** | EQ-5D-5L Mobility | - Major improvement: ≤-3 point change - Moderate improvement: -2 point change - Minimal improvement: -1 point change - Stable: no change - Minimal worsening: 1 point change - Moderate worsening: 2 point change - Major worsening: ≥3 point change | - DRS-P |
| **5** | EQ-5D-5L VAS | - Major improvement: ≥ 21 point change - Moderate improvement: ≥14 to <21 point change - Minimal improvement: ≥7 to <14 point change - Stable: change less than 7 points (negative and positive) - Minimal worsening: ≤-7 to >-14 point change - Moderate worsening: ≤-14 to >-21 point change - Major worsening: ≤-21 point change | - NFBSI Total - DRS-P - DRS-E |
| **6** | ECOG | - Minimal improvement: -1 point change - Stable: 0 point change - Minimal worsening: 1 point change - Moderate to major worsening: ≥2 point change | - NFBSI Total - DRS-P |

Minimal and Moderate improvement/worsening groups were merged due to low sample size to form a Minimal-to-Moderate Improvement group and Minimal-to-Moderate Worsening group

**Supplementary Table 2.1: NFBSI-16 item-total correlations at Cycle 2**

|  | **Correlation with domain** | | | |
| --- | --- | --- | --- | --- |
|  | **NFBSI-16 Total (N=93)** | **NFBSI-16 DRS-P (N=93)** | **NFBSI-16 FWB (N=93)** |  |
|  | | | | |
| GP1: I have a lack of energy | 0.785* | 0.783* | 0.437 |  |
| GP4: I have pain | 0.612* | 0.627* | 0.289 |  |
| GP6: I feel ill | 0.716* | 0.665* | 0.505 |  |
| B1: I have been short of breath | 0.362* | 0.448* | 0.231 |  |
| GP3: I have trouble meeting family needs | 0.747* | 0.721* | 0.600 |  |
| BP1: I have bone pain | 0.668* | 0.729* | 0.400 |  |
| HI7: I feel fatigued | 0.608* | 0.624* | 0.229 |  |
| GF5: I am sleeping well | 0.340* | 0.324* | 0.418 |  |
| GE6: I worry condition will get worse | 0.330* | 0.328 | 0.107 |  |
| GP2: I have nausea | 0.558* | 0.573 | 0.264 |  |
| N6: I have mouth sores | 0.231* | 0.166 | 0.027 |  |
| GP5: I am bothered by treatment side effects | 0.576* | 0.506 | 0.302 |  |
| B5: I am bothered by hair loss | 0.266* | 0.230 | -0.048 |  |
| GF1: I am able to work | 0.498* | 0.517 | 0.628* |  |
| GF3: I am able to enjoy life | 0.480* | 0.408 | 0.790* |  |
| GF7: I am content with quality of my life right now | 0.589* | 0.513 | 0.787* |  |
|  | | | | |

*Table footnote: Abbreviations: NFBSI: National Comprehensive Cancer Network Functional Assessment Of Cancer Therapy-Breast Cancer Symptom Index; DRS-P: Disease-Related Symptoms Physical; FWB: Functional Wellbeing*

*Polychoric correlation coefficients on the day of the visit. Item-scale correlation corrected for overlap (relevant item removed from its scale for correlation) when the item belongs to the domain in question, as signified by *.*

**Supplementary Table 2.2: NFBSI-16 item-total correlations at Cycle 5**

|  | **Correlation with domain** | | | |
| --- | --- | --- | --- | --- |
|  | **NFBSI-16 Total (N=62)** | **NFBSI-16 DRS-P (N=62)** | **NFBSI-16 FWB (N=61)** |  |
|  | | | | |
| GP1: I have a lack of energy | 0.853* | 0.816* | 0.609 |  |
| GP4: I have pain | 0.585* | 0.672* | 0.138 |  |
| GP6: I feel ill | 0.815* | 0.731* | 0.483 |  |
| B1: I have been short of breath | 0.347* | 0.446* | -0.014 |  |
| GP3: I have trouble meeting family needs | 0.636* | 0.634* | 0.444 |  |
| BP1: I have bone pain | 0.747* | 0.757* | 0.489 |  |
| HI7: I feel fatigued | 0.716* | 0.772* | 0.222 |  |
| GF5: I am sleeping well | 0.257* | 0.191* | 0.308 |  |
| GE6: I worry condition will get worse | 0.508* | 0.435 | 0.415 |  |
| GP2: I have nausea | 0.524* | 0.562 | 0.343 |  |
| N6: I have mouth sores | 0.386* | 0.291 | 0.380 |  |
| GP5: I am bothered by treatment side effects | 0.549* | 0.554 | 0.331 |  |
| B5: I am bothered by hair loss | 0.315* | 0.292 | 0.089 |  |
| GF1: I am able to work | 0.516* | 0.397 | 0.695* |  |
| GF3: I am able to enjoy life | 0.578* | 0.452 | 0.778* |  |
| GF7: I am content with quality of my life right now | 0.588* | 0.467 | 0.696* |  |
|  | | | | |

*Table footnote: Abbreviations: NFBSI: National Comprehensive Cancer Network Functional Assessment Of Cancer Therapy-Breast Cancer Symptom Index; DRSP: Disease-Related Symptoms Physical; FWB: Functional Wellbeing*

*Polychoric correlation coefficients on the day of the visit. Item-scale correlation corrected for overlap (relevant item removed from its scale for correlation) when the item belongs to the domain in question, as signified by *.*

**Supplementary Table 3: NFBSI-16 correlations with anchor measures change from baseline**

| NFBSI-19 Score/Anchor | Cycle 5 | | Cycle 7 | | Cycle 9 | |
| --- | --- | --- | --- | --- | --- | --- |
|  | N | Correlation Coefficient | N | Correlation Coefficient | N | Correlation Coefficient |
| NFBSI-16 Total Score* |  |  |  |  |  |  |
| EQ-5D-5L VAS | 57 | **-0.354** | 47 | **-0.332** | 33 | **-0.521** |
| ECOG | 60 | 0.127 | 48 | -0.081 | 34 | -0.085 |
|  |  |  |  |  |  |  |
| DRS-P* |  |  |  |  |  |  |
| EQ-5D-5L Pain | 58 | **-0.396** | 48 | **-0.437** | 34 | **-0.319** |
| EQ-5D-5L Usual Activities | 57 | **-0.332** | 47 | **-0.508** | 33 | **-0.488** |
| EQ-5D-5L Mobility | 56 | -0.255 | 47 | -0.172 | 33 | -0.267 |
| EQ-5D-5L VAS | 57 | -0.289 | 47 | -0.273 | 33 | **-0.571** |
| ECOG | 60 | 0.151 | 48 | 0.020 | 34 | -0.086 |
|  |  |  |  |  |  |  |
| DRS-E** |  |  |  |  |  |  |
| EQ-5D-5L Anxiety/Depression | 56 | -0.138 | 47 | -0.186 | 34 | 0.100 |
| EQ-5D-5L VAS | 56 | **-0.390** | 47 | -0.276 | 33 | **-0.361** |

*Polyserial correlation coefficients with anchors

**Polychoric correlation coefficients with anchors

Abbreviations: NFBSI: National Comprehensive Cancer Network Functional Assessment Of Cancer Therapy-Breast Cancer Symptom Index; VAS: Visual Analogue Scale; DRSP: Disease-Related Symptoms Physical; DRSE: Disease-Related Symptoms Emotional

Supplementary Table 4.1.1: Within and Between Groups Anchor-based Changes for the NFBSI-16 Total Score between Baseline and Cycle 5

| Anchor (ANC) | N (%) | Median | 10th Percentile | 25th Percentile | 75th Percentile | 90th Percentile | Within-group Mean Change Score (95% CI) | Mean Between-Group Change Score Difference vs Stable Group (95% CI) |
| --- | --- | --- | --- | --- | --- | --- | --- | --- |
| EQ-5D-5L VAS |  |  |  |  |  |  |  |  |
| Major improvement: >= 21 point increase | 6 (10.5%) | 3.37 | -15.00 | 1.00 | 12.00 | 14.00 | 3.12 (-7.69, 13.94) | 3.66 (-4.56, 11.88) |
| Minimal to moderate improvement: >=7 to <21 point increase | 19 (33.3%) | -4.00 | -22.00 | -9.00 | 3.43 | 14.00 | -2.61 (-8.03, 2.80) | -2.08 (-8.65, 4.50) |
| Stable: change less than 7 points (negative and positive) | 17 (29.8%) | 0.00 | -11.00 | -5.00 | 4.53 | 9.20 | -0.54 (-4.44, 3.37) |  |
| Minimal to moderate worsening: <=-7 to >-21 point decrease | 10 (17.5%) | -8.00 | -16.50 | -10.44 | -3.43 | 0.00 | -7.71 (-12.20, -3.23) | -7.18 (-13.04, -1.31) |
| Major worsening: <=-21 point decrease | 5 (8.8%) | -12.00 | -19.00 | -15.00 | -7.27 | 8.00 | -9.05 (-22.03, 3.92) | -8.52 (-17.27, 0.24) |

Abbreviations: NFBSI: National Comprehensive Cancer Network Functional Assessment Of Cancer Therapy-Breast Cancer Symptom Index; VAS: Visual Analogue Scale; DRSP: Disease-Related Symptoms Physical; DRSE: Disease-Related Symptoms Emotional

Supplementary Table 4.1.2: Within and Between Groups Anchor-based Changes for the NFBSI-16 Total Score between Baseline and Cycle 7

| Anchor (ANC) | N (%) | Median | 10th Percentile | 25th Percentile | 75th Percentile | 90th Percentile | Within-group Mean Change Score (95% CI) | Mean Between-Group Change Score Difference vs Stable Group (95% CI) |
| --- | --- | --- | --- | --- | --- | --- | --- | --- |
| EQ-5D-5L VAS |  |  |  |  |  |  |  |  |
| Major improvement: >= 21 point increase | 6 (12.8%) | 5.00 | -6.00 | -5.86 | 11.00 | 14.00 | 3.86 (-5.29, 13.01) | 6.74 (-0.26, 13.75) |
| Minimal to moderate improvement: >=7 to <21 point increase | 9 (19.1%) | -1.00 | -12.00 | -4.00 | 3.00 | 17.00 | -0.22 (-6.55, 6.10) | 2.66 (-3.32, 8.65) |
| Stable: change less than 7 points (negative and positive) | 22 (46.8%) | -3.50 | -12.00 | -9.00 | 2.00 | 7.00 | -2.89 (-6.01, 0.24) |  |
| Minimal to moderate worsening: <=-7 to >-21 point decrease | 6 (12.8%) | -6.79 | -26.00 | -14.00 | 3.00 | 6.00 | -7.43 (-20.33, 5.47) | -4.54 (-12.42, 3.33) |
| Major worsening: <=-21 point decrease | 4 (8.5%) | -2.93 | -9.67 | -6.83 | 2.57 | 7.00 | -2.13 (-13.15, 8.88) | 0.75 (-7.14, 8.64) |

Abbreviations: NFBSI: National Comprehensive Cancer Network Functional Assessment Of Cancer Therapy-Breast Cancer Symptom Index; VAS: Visual Analogue Scale; DRSP: Disease-Related Symptoms Physical; DRSE: Disease-Related Symptoms Emotional

Supplementary Table 4.1.3: Within and Between Groups Anchor-based Changes for the NFBSI-16 Total Score between Baseline and Cycle 9

| Anchor (ANC) | N (%) | Median | 10th Percentile | 25th Percentile | 75th Percentile | 90th Percentile | Within-group Mean Change Score (95% CI) | Mean Between-Group Change Score Difference vs Stable Group (95% CI) |
| --- | --- | --- | --- | --- | --- | --- | --- | --- |
| EQ-5D-5L VAS |  |  |  |  |  |  |  |  |
| Major improvement: >= 21 point increase | 5 (15.2%) | 4.20 | -3.43 | 2.00 | 7.00 | 10.00 | 3.95 (-2.38, 10.29) | 5.16 (-1.65, 11.97) |
| Minimal to moderate improvement: >=7 to <21 point increase | 4 (12.1%) | -3.50 | -12.00 | -9.50 | 2.00 | 4.00 | -3.75 (-15.10, 7.60) | -2.55 (-10.45, 5.35) |
| Stable: change less than 7 points (negative and positive) | 16 (48.5%) | -0.87 | -12.00 | -5.00 | 2.14 | 6.00 | -1.20 (-4.74, 2.34) |  |
| Minimal to moderate worsening: <=-7 to >-21 point decrease | 7 (21.2%) | -7.00 | -16.00 | -11.00 | -2.27 | 0.00 | -7.59 (-12.57, -2.61) | -6.39 (-12.33, -0.44) |
| Major worsening: <=-21 point decrease | 1 (3.0%) | -9.00 | -9.00 | -9.00 | -9.00 | -9.00 | -9.00 (-, -) | -7.80 (-22.39, 6.79) |

Abbreviations: NFBSI: National Comprehensive Cancer Network Functional Assessment Of Cancer Therapy-Breast Cancer Symptom Index; VAS: Visual Analogue Scale; DRSP: Disease-Related Symptoms Physical; DRSE: Disease-Related Symptoms Emotional

Supplementary Table 4.2.1: Within and Between Groups Anchor-based Changes for the NFBSI-16 DRS-P between Baseline and Cycle 5

| Anchor (ANC) | N (%) | Median | 10th Percentile | 25th Percentile | 75th Percentile | 90th Percentile | Within-group Mean Change Score (95% CI) | Mean Between-Group Change Score Difference vs Stable Group (95% CI) |
| --- | --- | --- | --- | --- | --- | --- | --- | --- |
| EQ-5D-5L Pain |  |  |  |  |  |  |  |  |
| Major improvement: <=-3 point change | 0 (0.0%) |  |  |  |  |  |  |  |
| Minimal to moderate improvement: -1 or -2 point change | 20 (34.5%) | 0.50 | -7.83 | -4.50 | 5.50 | 8.00 | 0.65 (-2.32, 3.61) | 0.45 (-2.79, 3.70) |
| Stable: 0 point change | 24 (41.4%) | 0.43 | -7.00 | -2.00 | 2.64 | 7.00 | 0.19 (-1.62, 2.00) |  |
| Minimal to moderate worsening: 1 or 2 point change | 14 (24.1%) | -4.00 | -8.00 | -6.00 | -0.71 | 1.00 | -3.60 (-5.85, -1.35) | -3.79 (-6.62, -0.96) |
| Major worsening: >=3 point change | 0 (0.0%) |  |  |  |  |  |  |  |
|  |  |  |  |  |  |  |  |  |
| EQ-5D-5L Usual Activities |  |  |  |  |  |  |  |  |
| Major improvement: <=-3 point change | 0 (0.0%) |  |  |  |  |  |  |  |
| Minimal to moderate improvement: -1 or -2 point change | 6 (10.5%) | 3.50 | -1.00 | 0.00 | 7.00 | 13.00 | 4.33 (-1.29, 9.95) | 5.15 (0.75, 9.56) |
| Stable: 0 point change | 38 (66.7%) | -0.36 | -8.00 | -5.00 | 3.00 | 5.00 | -0.82 (-2.44, 0.80) |  |
| Minimal to moderate worsening: 1 or 2 point change | 13 (22.8%) | -2.00 | -8.00 | -6.00 | 0.86 | 7.00 | -2.05 (-5.27, 1.16) | -1.23 (-4.48, 2.01) |
| Major worsening: >=3 point change | 0 (0.0%) |  |  |  |  |  |  |  |

Abbreviations: NFBSI: National Comprehensive Cancer Network Functional Assessment Of Cancer Therapy-Breast Cancer Symptom Index; VAS: Visual Analogue Scale; DRSP: Disease-Related Symptoms Physical; DRSE: Disease-Related Symptoms Emotional

Supplementary Table 4.2.2: Within and Between Groups Anchor-based Changes for the NFBSI-16 DRS-P between Baseline and Cycle 7

| Anchor (ANC) | N (%) | Median | 10th Percentile | 25th Percentile | 75th Percentile | 90th Percentile | Within-group Mean Change Score (95% CI) | Mean Between-Group Change Score Difference vs Stable Group (95% CI) |
| --- | --- | --- | --- | --- | --- | --- | --- | --- |
| EQ-5D-5L Pain |  |  |  |  |  |  |  |  |
| Major improvement: <=-3 point change | 0 (0.0%) |  |  |  |  |  |  |  |
| Minimal to moderate improvement: -1 or -2 point change | 14 (29.2%) | 3.00 | -2.00 | 0.00 | 5.00 | 6.00 | 2.40 (0.71, 4.09) | 2.77 (-0.53, 6.07) |
| Stable: 0 point change | 18 (37.5%) | -0.50 | -7.00 | -4.00 | 5.00 | 5.43 | -0.37 (-3.08, 2.34) |  |
| Minimal to moderate worsening: 1 or 2 point change | 16 (33.3%) | -4.00 | -10.00 | -7.00 | -1.00 | 0.00 | -3.63 (-6.36, -0.89) | -3.25 (-6.97, 0.46) |
| Major worsening: >=3 point change | 0 (0.0%) |  |  |  |  |  |  |  |
|  |  |  |  |  |  |  |  |  |
| EQ-5D-5L Usual Activities |  |  |  |  |  |  |  |  |
| Major improvement: <=-3 point change | 1 (2.1%) | 4.00 | 4.00 | 4.00 | 4.00 | 4.00 | 4.00 (-, -) | 3.66 (-5.69, 13.00) |
| Minimal to moderate improvement: -1 or -2 point change | 7 (14.9%) | 1.00 | -6.00 | -2.00 | 5.00 | 10.00 | 1.57 (-3.11, 6.25) | 1.23 (-2.69, 5.14) |
| Stable: 0 point change | 30 (63.8%) | 0.00 | -4.33 | -3.00 | 4.57 | 5.71 | 0.34 (-1.33, 2.02) |  |
| Minimal to moderate worsening: 1 or 2 point change | 8 (17.0%) | -6.00 | -10.43 | -9.00 | -3.00 | 5.00 | -5.18 (-9.39, -0.97) | -5.52 (-9.24, -1.81) |
| Major worsening: >=3 point change | 1 (2.1%) | -11.00 | -11.00 | -11.00 | -11.00 | -11.00 | -11.00 (-, -) | -11.34 (-20.69, -2.00) |

Abbreviations: NFBSI: National Comprehensive Cancer Network Functional Assessment Of Cancer Therapy-Breast Cancer Symptom Index; VAS: Visual Analogue Scale; DRSP: Disease-Related Symptoms Physical; DRSE: Disease-Related Symptoms Emotional

Supplementary Table 4.2.3: Within and Between Groups Anchor-based Changes for the NFBSI-16 DRS-P between Baseline and Cycle 9

| Anchor (ANC) | N (%) | Median | 10th Percentile | 25th Percentile | 75th Percentile | 90th Percentile | Within-group Mean Change Score (95% CI) | Mean Between-Group Change Score Difference vs Stable Group (95% CI) |
| --- | --- | --- | --- | --- | --- | --- | --- | --- |
| EQ-5D-5L Pain |  |  |  |  |  |  |  |  |
| Major improvement: <=-3 point change | 1 (2.9%) | 2.57 | 2.57 | 2.57 | 2.57 | 2.57 | 2.57 (-, -) | 4.59 (-5.21, 14.39) |
| Minimal to moderate improvement: -1 or -2 point change | 8 (23.5%) | 0.50 | -3.00 | -2.00 | 3.50 | 5.00 | 0.75 (-1.77, 3.27) | 2.77 (-0.88, 6.42) |
| Stable: 0 point change | 15 (44.1%) | 0.00 | -8.43 | -5.00 | 1.00 | 3.00 | -2.02 (-4.47, 0.43) |  |
| Minimal to moderate worsening: 1 or 2 point change | 10 (29.4%) | -2.50 | -5.50 | -4.00 | -1.00 | 3.50 | -2.00 (-4.61, 0.61) | 0.02 (-3.48, 3.51) |
| Major worsening: >=3 point change | 0 (0.0%) |  |  |  |  |  |  |  |
|  |  |  |  |  |  |  |  |  |
| EQ-5D-5L Usual Activities |  |  |  |  |  |  |  |  |
| Major improvement: <=-3 point change | 0 (0.0%) |  |  |  |  |  |  |  |
| Minimal to moderate improvement: -1 or -2 point change | 7 (21.2%) | 0.00 | -4.00 | -4.00 | 4.00 | 7.00 | 0.29 (-3.52, 4.09) | 0.80 (-2.31, 3.90) |
| Stable: 0 point change | 18 (54.5%) | 0.00 | -5.00 | -3.00 | 1.00 | 4.00 | -0.51 (-2.04, 1.01) |  |
| Minimal to moderate worsening: 1 or 2 point change | 8 (24.2%) | -4.00 | -12.00 | -7.21 | -2.00 | 3.00 | -4.43 (-8.24, -0.62) | -3.92 (-7.04, -0.79) |
| Major worsening: >=3 point change | 0 (0.0%) |  |  |  |  |  |  |  |

Abbreviations: NFBSI: National Comprehensive Cancer Network Functional Assessment Of Cancer Therapy-Breast Cancer Symptom Index; VAS: Visual Analogue Scale; DRSP: Disease-Related Symptoms Physical; DRSE: Disease-Related Symptoms Emotional

Supplementary Table 4.3.1: Within and Between Groups Anchor-based Changes for the NFBSI-16 DRS-E between Baseline and Cycle 5

| Anchor (ANC) | N (%) | Median | 10th Percentile | 25th Percentile | 75th Percentile | 90th Percentile | Within-group Mean Change Score (95% CI) | Mean Between-Group Change Score Difference vs Stable Group (95% CI) |
| --- | --- | --- | --- | --- | --- | --- | --- | --- |
| EQ-5D-5L VAS |  |  |  |  |  |  |  |  |
| Major improvement: >= 21 point increase | 6 (10.7%) | 1.50 | 0.00 | 1.00 | 2.00 | 4.00 | 1.67 (0.23, 3.10) | 1.14 (0.01, 2.27) |
| Minimal to moderate improvement: >=7 to <21 point increase | 18 (32.1%) | 0.00 | -1.00 | 0.00 | 1.00 | 2.00 | 0.28 (-0.48, 1.04) | -0.25 (-1.16, 0.66) |
| Stable: change less than 7 points (negative and positive) | 17 (30.4%) | 0.00 | -1.00 | 0.00 | 1.00 | 2.00 | 0.53 (-0.02, 1.08) |  |
| Minimal to moderate worsening: <=-7 to >-21 point decrease | 10 (17.9%) | -0.50 | -1.50 | -1.00 | 0.00 | 0.50 | -0.50 (-1.11, 0.11) | -1.03 (-1.85, -0.21) |
| Major worsening: <=-21 point decrease | 5 (8.9%) | -1.00 | -2.00 | -2.00 | 0.00 | 3.00 | -0.40 (-2.97, 2.17) | -0.93 (-2.34, 0.48) |

Abbreviations: NFBSI: National Comprehensive Cancer Network Functional Assessment Of Cancer Therapy-Breast Cancer Symptom Index; VAS: Visual Analogue Scale; DRSP: Disease-Related Symptoms Physical; DRSE: Disease-Related Symptoms Emotional

Supplementary Table 4.3.2: Within and Between Groups Anchor-based Changes for the NFBSI-16 DRS-E between Baseline and Cycle 7

| Anchor (ANC) | N (%) | Median | 10th Percentile | 25th Percentile | 75th Percentile | 90th Percentile | Within-group Mean Change Score (95% CI) | Mean Between-Group Change Score Difference vs Stable Group (95% CI) |
| --- | --- | --- | --- | --- | --- | --- | --- | --- |
| EQ-5D-5L VAS |  |  |  |  |  |  |  |  |
| Major improvement: >= 21 point increase | 6 (12.8%) | 1.00 | -1.00 | 0.00 | 3.00 | 3.00 | 1.17 (-0.64, 2.97) | 0.80 (-0.63, 2.24) |
| Minimal to moderate improvement: >=7 to <21 point increase | 9 (19.1%) | 0.00 | -1.00 | 0.00 | 2.00 | 2.00 | 0.67 (-0.19, 1.53) | 0.30 (-0.81, 1.42) |
| Stable: change less than 7 points (negative and positive) | 22 (46.8%) | 0.00 | -1.00 | 0.00 | 1.00 | 2.00 | 0.36 (-0.29, 1.01) |  |
| Minimal to moderate worsening: <=-7 to >-21 point decrease | 6 (12.8%) | 0.00 | -1.00 | 0.00 | 1.00 | 1.00 | 0.17 (-0.62, 0.96) | -0.20 (-1.48, 1.09) |
| Major worsening: <=-21 point decrease | 4 (8.5%) | -1.00 | -1.00 | -1.00 | 1.00 | 3.00 | 0.00 (-3.18, 3.18) | -0.36 (-2.09, 1.37) |

Abbreviations: NFBSI: National Comprehensive Cancer Network Functional Assessment Of Cancer Therapy-Breast Cancer Symptom Index; VAS: Visual Analogue Scale; DRSP: Disease-Related Symptoms Physical; DRSE: Disease-Related Symptoms Emotional

Supplementary Table 4.3.3: Within and Between Groups Anchor-based Changes for the NFBSI-16 DRS-E between Baseline and Cycle 9

| Anchor (ANC) | N (%) | Median | 10th Percentile | 25th Percentile | 75th Percentile | 90th Percentile | Within-group Mean Change Score (95% CI) | Mean Between-Group Change Score Difference vs Stable Group (95% CI) |
| --- | --- | --- | --- | --- | --- | --- | --- | --- |
| EQ-5D-5L VAS |  |  |  |  |  |  |  |  |
| Major improvement: >= 21 point increase | 5 (15.2%) | 1.00 | 0.00 | 0.00 | 2.00 | 3.00 | 1.20 (-0.42, 2.82) | 0.95 (-0.14, 2.04) |
| Minimal to moderate improvement: >=7 to <21 point increase | 4 (12.1%) | 0.50 | -1.00 | -0.50 | 1.50 | 2.00 | 0.50 (-1.55, 2.55) | 0.25 (-0.92, 1.42) |
| Stable: change less than 7 points (negative and positive) | 16 (48.5%) | 0.00 | -1.00 | 0.00 | 1.00 | 2.00 | 0.25 (-0.25, 0.75) |  |
| Minimal to moderate worsening: <=-7 to >-21 point decrease | 7 (21.2%) | 0.00 | -1.00 | -1.00 | 1.00 | 1.00 | 0.00 (-0.76, 0.76) | -0.25 (-1.10, 0.60) |
| Major worsening: <=-21 point decrease | 1 (3.0%) | 0.00 | 0.00 | 0.00 | 0.00 | 0.00 | 0.00 (-, -) | -0.25 (-2.30, 1.80) |

Abbreviations: NFBSI: National Comprehensive Cancer Network Functional Assessment Of Cancer Therapy-Breast Cancer Symptom Index; VAS: Visual Analogue Scale; DRSP: Disease-Related Symptoms Physical; DRSE: Disease-Related Symptoms Emotional

Supplementary Table 5: Distribution based change estimates: NFBSI-16 and GP5 at baseline

| Subscale | n | ½ SD | SEM (lower, upper) |
| --- | --- | --- | --- |
| NFBSI-16 Total | 97 | 5.097 | 3.210, 4.055 |
| NFBSI-16 DRS-P | 97 | 3.313 | 2.035, 3.786 |
| NFBSI-16 DRS-E | 96 | 0.673 | 0.743, 0.812 |
| NFBSI-16 TSE | 97 | 1.071 | 1.211, 1.484 |
| NFBSI-16 FWB | 97 | 1.609 | 1.690, 1.760 |
| GP5: I am bothered by treatment side effects | 89 | 0.446 | 0.554, 0.627 |
